# Identification of Potential Biomarkers of Platelet RNA in Glioblastoma by Bioinformatics Analysis

**DOI:** 10.1155/2022/2488139

**Published:** 2022-08-12

**Authors:** Xianjun Chen, Qianxia Lin, Yong'an Jiang, Changfeng Wang, Feixiang Min, Yue Ou, Yinghua Xia, Gui Yu, Ru'en Liu

**Affiliations:** ^1^Medical College of Nanchang University, Nanchang, Jiangxi 330006, China; ^2^Department of Neurosurgery, Jiangxi Provincial People's Hospital, Nanchang, Jiangxi 330006, China; ^3^Vascular Breast Surgery, Jiangxi Provincial People's Hospital, Nanchang, Jiangxi 330006, China; ^4^Jiangxi University of Traditional Chinese Medicine, Nanchang, Jiangxi 330006, China; ^5^Department of Neurosurgery, The Second Affiliated Hospital of Nanchang University, Nanchang, Jiangxi 330006, China; ^6^The First Clinical Medical College, Gannan Medical University, Ganzhou, Jiangxi 341000, China; ^7^Department of Neurosurgery, Peking University People's Hospital, 11th Xizhimen South St. Beijing 100044, China

## Abstract

**Objective:**

Glioblastoma is one of the most common and fatal malignancies in adults. Current treatment is still not optimistic. Glioblastoma (GBM) transports RNA to platelets in the blood system via microvesicles, suggesting that platelet RNA can be a potential diagnostic and therapeutic target. The roles of specific platelet RNAs in treatment of GBM are not well understood.

**Methods:**

Platelet RNA profiling of 8 GBM and 12 normal samples were downloaded from the GEO database. Differentially expressed genes (DEGs) were identified between tumors and normal samples. Gene Ontology (GO) and Kyoto Encyclopedia of Genes and Genomes (KEGG) analyses were performed to elucidate the functions of up- and downregulated genes. miRNA was predicted by miRTarBase, TargetScan, and miRDB databases. circBase and circBank were used for circRNA prediction. ceRNA (circRNA-mRNA-miRNA) network was constructed to investigate the potential interactions.

**Results:**

22 genes were upregulated and 9 genes were downregulated. There are only two genes (*CCR7* and *FAM102A*) that connect to miRNAs (hsa-let-7a-5p, hsa-miR-1-3p). We assessed the overall survival rates by Kaplan-Meier plotter, and relative expression of GBM and subtypes for overlapped mRNA (*CCR7* and *FAM102A*) were evaluated, and further, we obtained circRNAs (has-circ-0015164, hsa-circ-0003243) by circBank and circBase and bind sites through the CSCD database. Finally, a ceRNA network (circRNA-mRNA-miRNA) was constructed based on 2 miRNAs, 2 mRNAs, and 2 circRNAs by Cytoscape. This study focused on potential mRNA and ceRNA biomarkers to targeted treatment of GBM and provided ideas for clinical treatment through the combination of hematology and oncology.

**Conclusion:**

The findings of this study contribute to better understand the relationship between GBM and the blood system (platelets) and might lay a solid foundation for improving GBM molecule and gene diagnosis and prognosis.

## 1. Introduction

Glioblastoma (GBM) is the most common malignant tumor in the central system [1]. The existing clinical treatment options are mainly surgical treatment, drug treatment, and radiotherapy and chemotherapy [2]. Targeted tumor therapy and personalized drug therapy can greatly improve the survival rate of patients, mainly depending on the disease spectrum and the development of concomitant diagnosis. The detection of tumor nucleic acid is highly predictive for the targeted treatment of tumors [3]. In the 2016 WHO classification of gliomas, a large part of the classification of diffuse gliomas was based on IDH mutations [1]. Therefore, as powerful means for the treatment of personalized patients, nucleic acid detection of glioblastoma will be utilized in future clinical practice. However, acquisition of high-quality nucleic acids is still an obstacle to our progress [4, 5]. There are usually a large number of platelets in the blood, up to 150,000-350,000 [6, 7]. Platelets in the body provide multiple sources of biomarkers for clinical and scientific research. It has been reported that platelets absorb membrane vesicles secreted by tumors and may contain tumor-associated RNA [8, 9]. Therefore, exploration of tumor platelet nucleic acids provides potential sources of biomarkers for targeted therapy of cancer and cancer diagnosis.

MicroRNAs (miRNAs) are endogenously expressed noncoding RNAs that participate in biological processes such as proliferation and apoptosis [10] and can be used as biomolecules that promote or inhibit tumors [11]. At present, miRNA has been used as a clinical biomarker for patient stratification and prognosis and as an emerging biotherapy target or drug for clinical selection [12, 13], inhibition, or activation of microRNA-146 B on the expression of PTP1B in gastric cancer cells [14]. Circular RNA is a new type of endogenous noncoding RNA, which is characterized by its covalent closed loop structure without 5′cap or 3′poly A tail. At the same time, it has higher tolerance to exonuclease, and circRNA is involved in the progress of tumors. circRNA is a covalently biomolecule with tissue-specific and cell-specific expression patterns in eukaryotes [15, 16]. Unlike the structure of linear RNA [17], circRNA is resistant to exonuclease and is more stable than linear RNA [18]. Continuous research has found that circular RNA plays an important role in the regulation of cancer growth. circPTN inhibits miR-145-5p and miR-330-5p through sponge formation and promotes glioma proliferation and stemness [19]. circNT5E acts as a sponge against miR422a and regulates the proliferation, migration, and invasion of glioblastoma [20]. circTTBK2 (but nonlinear TTBK2) acts as a miR-217 sponge in a sequence-specific manner. circTTBK2 promotes the malignant progression of gliomas by regulating the miR-217/HNF1*β*/Derlin-1 pathway [21]. Circular RNA circSCAF11 promotes tumor progression through the miR-421/SP1/VEGFA pathway. Therefore, ceRNA (miRNA and circRNA) can be used as a new choice for molecular diagnosis and treatment of accelerated tumors (GBM).

Our research is dedicated to obtaining GBM and normal human mRNA sequencing results from GEO and identifying important mRNAs involved in GBM through bioinformatics analysis. In addition, the GO and KEGG results for important targets (upregulated and downregulated mRNA) revealed the potential biological function of target genes. Finally, in order to construct a regulatory network, we established an important circRNA-mRNA-miRNA loop. This helps to determine the important role played by FAM102A and CCR7 and the corresponding ceRNA.

## 2. Materials and Methods

### 2.1. The mRNA Microarray Data Acquisition and Processing

**Figure 1:**
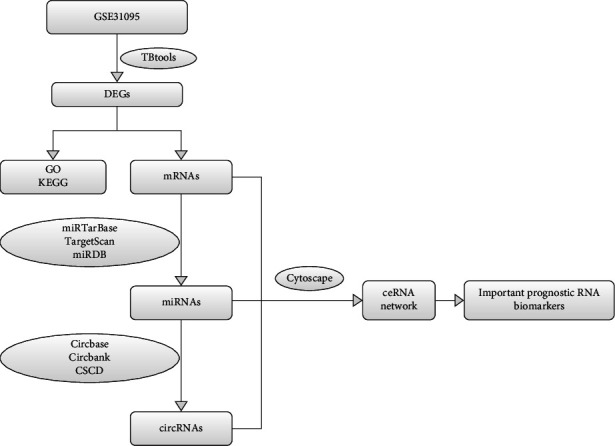
Flowchart for bioinformatics analysis of publicly available data from GEO database. Download GSE31095 to identify mRNAs, miRNAs, and circRNAs related to prognosis for the construction of ceRNA regulatory network. GO and KEGG enrichment analysis was established. The relationship between the expression level of hub genes and survival prognosis was determined by Kaplan-Meier survival analysis. GO: Gene Ontology; KEGG: Kyoto Encyclopedia of Genes and Genomes; CSCD: cancer-specific circRNA database; mRNA: messenger RNA; miRNA: microRNA; circRNA: circular RNA; ceRNA: competing endogenous RNA.

We obtained GSE31095 from the GEO database (https://www.ncbi.nlm.nih.gov/geo/), an accessible public functional genomics data repository. The GSE31095 dataset contained platelet mRNAs from 12 healthy people and 8 GBM patients. The profiling was normalized and log transformed. DEGs were identified with log2FC > 2 and FDR < 0.05, and these DEGs were visualized using a toolkit for biologists integrating various biological data-handling tools (TBtools; https://github.com/CJ-Chen/TBtools/releases). The flowchart of the study is shown in Figure 1.

### 2.2. GO and KEGG Pathway Analyses of Selected DEGs

We analyzed the potential functions of these DEGs including upregulated genes and downregulated genes with the Functional Enrichment Analysis Tool (Funrich; https://www.funrich.org), a stand-alone software tool used mainly for functional enrichment and interaction network analysis of genes and proteins. Gene Ontology categories included cellular component (CC), molecular function (MF), biological process (BP), and biological pathways of these DEGs referenced from Gene Ontology(GO) and Kyoto Encyclopedia of Genes and Genomes (KEGG), and *P* < 0.05 was considered to be statistically significant.

### 2.3. MicroRNA and circRNA Prediction and ceRNA Construction

We used TargetScan (http://www.targetscan.org/vert_71/), miRTarBase (http://mirtarbase.mbc.nctu.edu.tw/php/index.php), and miRDB (http://mirdb.org/) to predict the relationships between the overlapped mRNAs and miRNAs. circBase (http://www.circbase.org/) and circBank (http://www.circbank.cn/help.html) were employed to explore the selected miRNAs and corresponding circRNAs for further analysis. We explored the function of circRNA through the cancer-specific circRNA database (CSCD), a database developed for cancer-specific circRNAs, and collected available RNA sequencing datasets from 87 cancer cell line samples, to build the basic structure of circular RNA. Finally, Cytoscape version 3.7.2 was performed to construct and visualize a circRNA-mRNA-miRNA regulatory network.

### 2.4. Gene Expression and Targeted Gene Survival Analysis

GEPIA (http://gepia.cancer-pku.cn/) was used to calculate the expression and prognosis of key mRNAs in GBM. The criteria for significance were |log2 (fold change [FC])| > 1 and adjusted *P* < 0.01. For gene overall survival analysis, we used the Kaplan-Meier curves and log-rank test, with *P* < 0.05 as the best threshold for significance difference.

### 2.5. Statistical Analysis

The expression levels of circRNAs were analyzed by SPSS 23.0 (IBM Corp., Armonk, NY). Figures were constructed using GraphPad Prism 7 (GraphPad Software, La Jolla, California), Office Visio (Microsoft, Washington), and Adobe Illustrator CS6 (Adobe, California). *P* < 0.05 is defined as statistically significant differences.

## 3. Results

### 3.1. Identification of DEGs between Normal and GBM Samples

**Figure 2:**
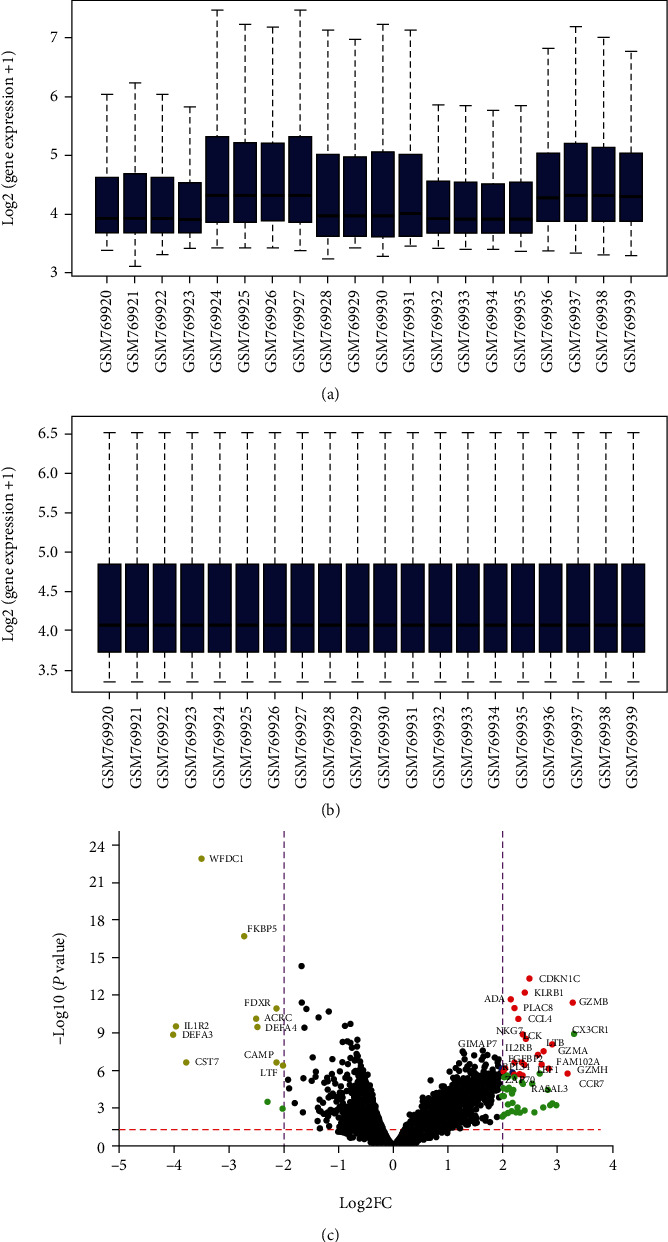
Visualization and standardization of gene expression and differential expression of data. (a) Visualizing gene expression as boxplot. (b) Standardizing gene expression of GSE31095 data. (c) Red points represent the upregulated RNAs based on screening |fold change| > 2.0, corrected *P* value < 0.05, and FDR < 0.05; gray points represent downregulated RNAs, based on |fold change| > 2.0, corrected *P* value < 0.05, and FDR < 0.05. The green points indicate |fold change| > 2.0, corrected *P* value < 0.05, but FDR > 0.05. Blue points represent noncorresponding gene symbol, and black points represent RNAs with no significant difference. FC: fold change.

**Figure 3:**
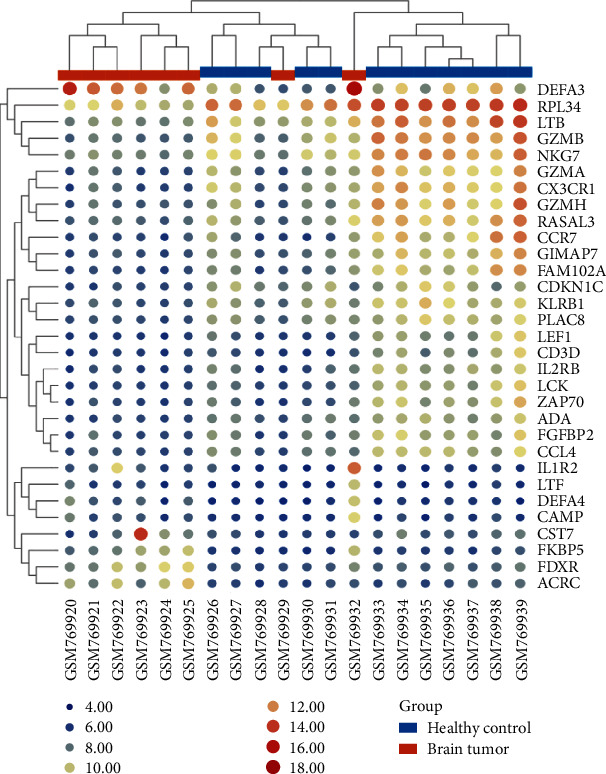
Heat map of up- and downregulated DEGs based on |fold change| > 2.0, corrected *P* value < 0.05, and FDR < 0.05. (a) GSE31095 data, red represents that the expression of RNAs is relatively upregulated genes, blue represents that the expression of RNAs is downregulated, and white represents no significant changes.

We downloaded the GSE31095 glioma raw data from the GEO database, including the basic information of the mRNA of 12 healthy people and 8 glioma patients, which is shown in Supplement Files: Table S1. Further, we standardized the information of 20 samples as shown in Figures 2(a) and 2(b). We used software to screen the GSE31095 data (correct *P* < 0.05, log2FC > 2, FDR < 0.05) and obtained 31 DEGs (Figure 2(c) and Supplement Files: Table S2). 22 upregulated mRNAs were identified, 9 downregulated mRNAs are shown in Supplement Files: Table S3, and Figure 3 shows the expression of 22 upregulated and 9 downregulated mRNAs in 20 microarray datasets (normal and glioma) by TBtools [22].

### 3.2. GO and KEGG Analyses of DEGs

**Figure 4:**
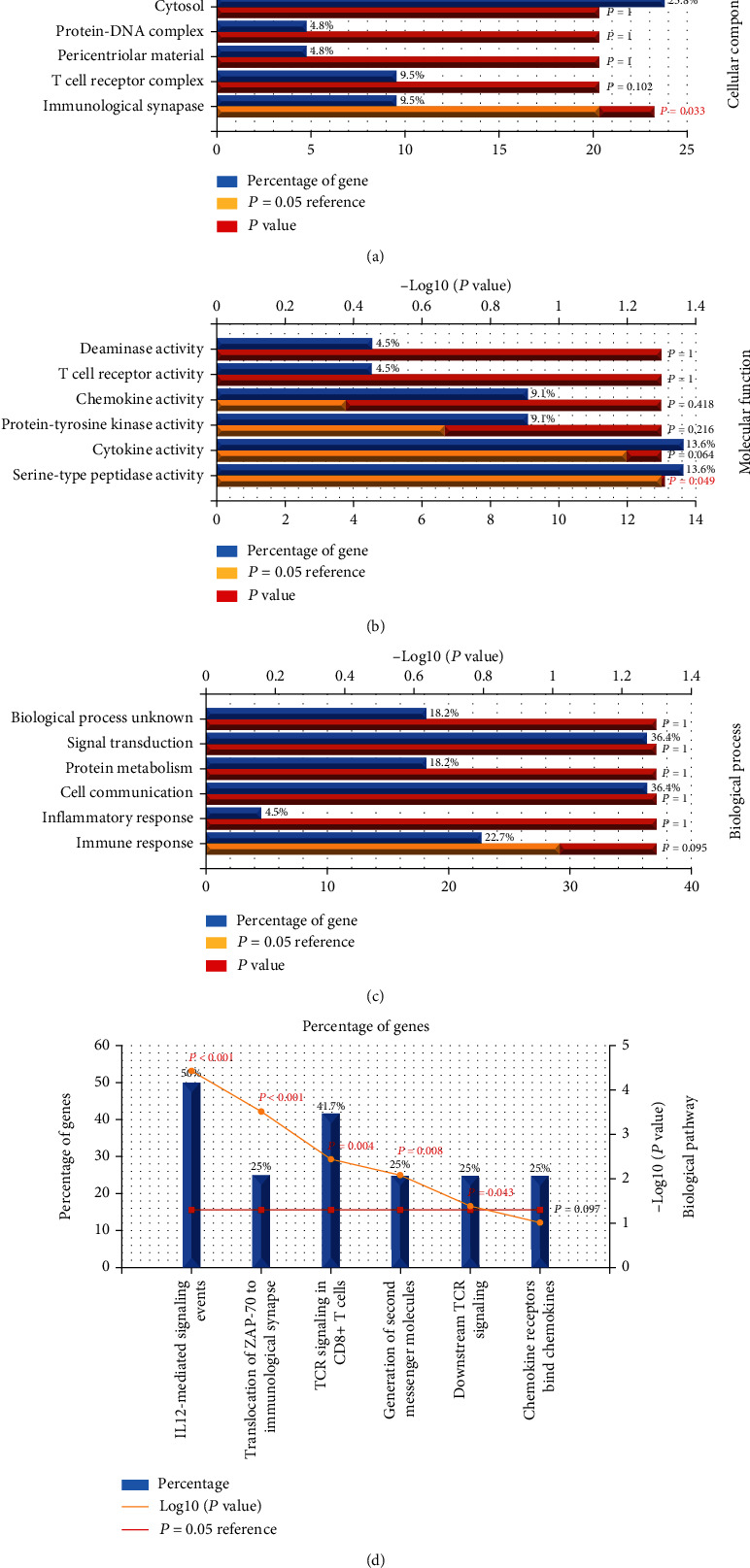
GO and KEGG analyses of 22 upregulated DEGs. (a–d) GO analysis of 3 functional groups: cellular component, molecular function, biological process. KEGG analysis of biological pathways. KEGG analysis of upregulated DEGs. Corrected *P* < 0.05.

**Figure 5:**
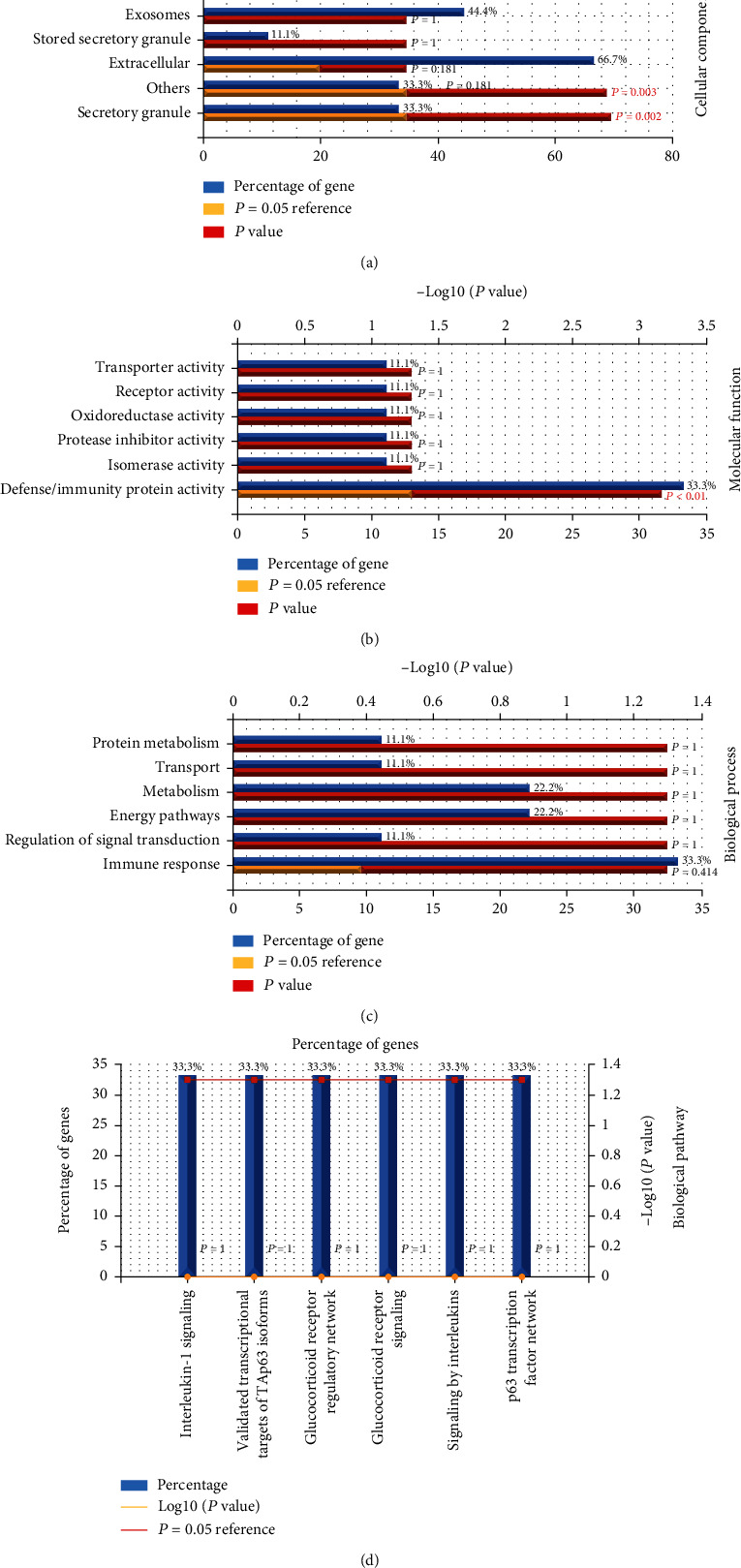
GO and KEGG analyses of 9 downregulated DEGs: (a–d) GO and KEGG analyses of downregulated DEGs. Corrected *P* < 0.05.

GO and KEGG analyses were performed to conduct cellular processes and fundamental signaling pathways upon biomarkers [23]. We further performed GO and KEGG analyses on 22 upregulated mRNAs and 9 downregulated mRNAs. As shown in Figure 4, upregulated mRNAs (Figures 4(a)–4(d)) were enriched in the cellular component (CC) in the immunological synapse (*P* = 0.033) (Figure 4(a)), and molecular function (MF) was enriched in serine-type peptidase activity (*P* = 0.049) (Figure 4(b)). And the analysis of 9 downregulated mRNAs (Figures 5(a)–5(d)) revealed that enrichments in CC (Figure 5(a)) were secretory granule (*P* = 0.002) and others (*P* = 0.003) and enrichments in MF were defense/immunity protein activity (*P* < 0.001) (Figure 5(b)). KEGG analysis of upregulated DEGs revealed the most likely enrichment pathway for biological pathways (BP) in IL12-mediated signaling events (*P* < 0.001), translocation of ZAP-70 to immunological synapse (*P* < 0.001), TCR signaling in CD8+ T cells (*P* = 0.004), generation of second messenger molecules (*P* = 0.008), and downstream TCR signaling (*P* = 0.043) (Figure 4(d)), while KEGG analysis of 9 downregulated DEGs are not significant (*P* > 0.05) (Figures 5(c)–5(d)).

### 3.3. miRNA Prediction of miRTarBase, TargetScan, and miRDB Databases

**Figure 6:**
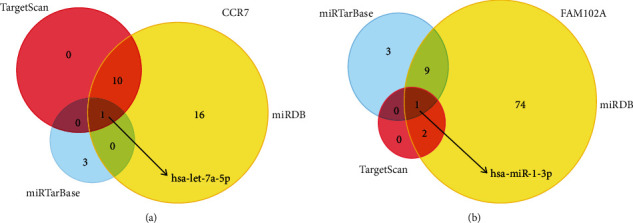
Target prediction of *CCR7* and *FAM102A*. (a, b) Venn diagram was used to select overlapped target-expressed miRNAs by miRTarBase, miRDB, and TargetScan databases.

miRNA has been identified as a key regulator in complex biological processes related to the pathogenesis of normal physiology and diseases, such as cancer [24]. Therefore, we chose target genes with common miRNAs for subsequent research analysis through miRTarBase, TargetScan, and miRDB databases to predict the miRNA corresponding to 31 mRNAs [25] and applied the Venn diagram to calculate the common miRNA. As shown in Figures 6(a) and 6(b), the upregulated mRNAs that overlapped in the 3 databases (hsa-let-7a-5p, hsa-miR-1-3p) were selected for further analysis.

### 3.4. Relative Expression and Overall Survival of the Key mRNAs (CCR7 and FAM102A) in GBM

**Figure 7:**
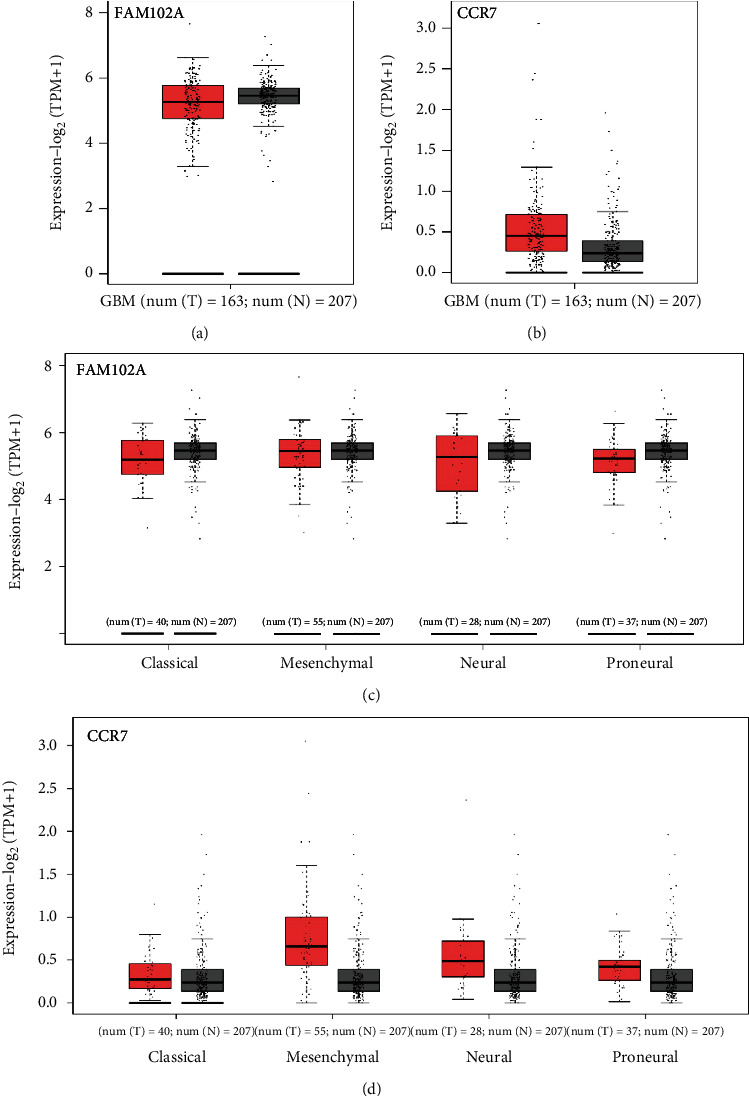
Expression pattern of overlapped DEGs (*CCR7* and *FAM102A*) for GBM (T), normal patients (N), and GBM subtypes: (a) *CCR7* expression of GBM; (b) *FAM102A* expression of GBM; (c) *CCR7* expression of GBM subtypes; (d) *FAM102A* expression of GBM subtypes.

**Figure 8:**
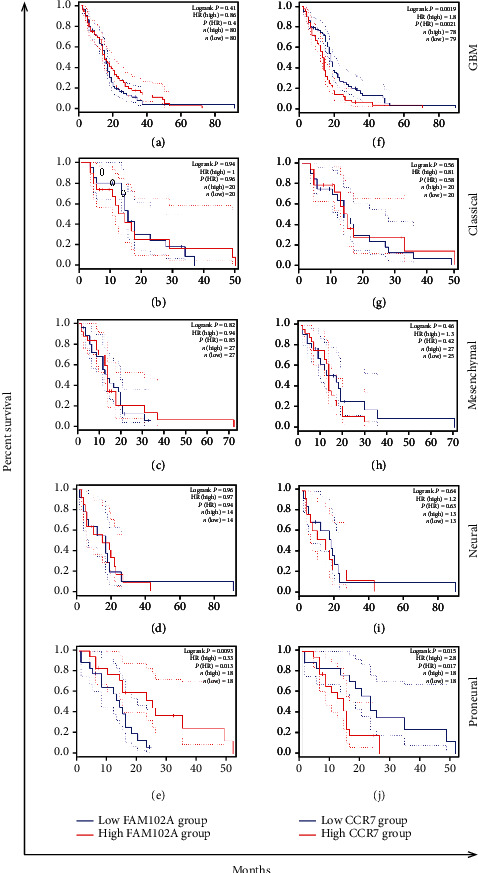
The overall survival analysis of DEGs (*CCR7* and *FAM102A*) for GBM and subtypes (proneural, neural, mesenchymal, and classical). (a–d) Percent survival of *CCR7* in GBM and GBM subtypes. (g–k) Percent survival of and *FAM102A* in GBM and GBM subtypes. |log_2_*FC*|cut off > 1, *P* cutoff < 0.01.

To confirm the expression and function of platelet mRNA *CCR7* and FAM102A in glioma patients, we used the GEPIA [26] to analyze the expression levels of *CCR7* and *FAM102A* in GBM and its subtypes by integrating TCGA and GTX data (|log2FC|cut off > 1, *P* cutoff < 0.01) in Figures 7(a)–7(d). As shown in Figures 8(a)–8(j), high expression of *CCR7* predicted unfavorable OS in all GBM cohorts (*P* = 0.0019), and a similar observation was seen in GBM patients with proneural subtype (*P* = 0.015). The expression of *FAM102A* in all GBM (*P* = 0.41) was not statistically significant, while increased expression of *FAM102A* was significantly correlated with poor OS in the proneural (*P* = 0.0093) subtype.

### 3.5. circRNA Prediction and Construction ceRNA Network

**Figure 9:**
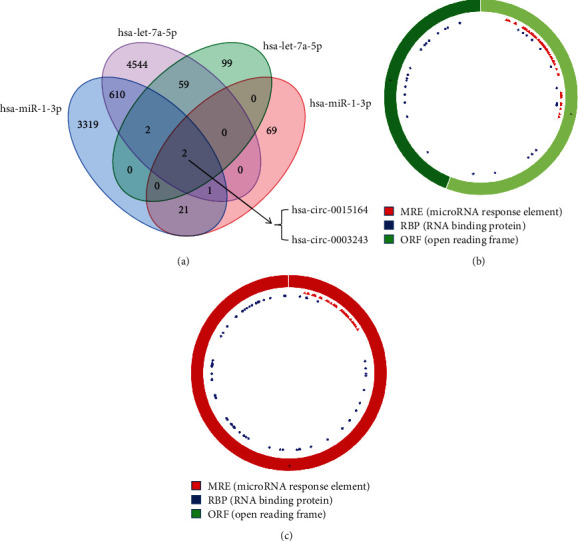
circRNA prediction and circRNA-mRNA-miRNA network construction. (a) circRNA prediction of 2 miRNAs (hsa-let-7a-5p, hsa-miR-1-3p) using circBase and circBank. (b, c) Structural pattern of overlapped circRNA hsa-circ-0003243 (b) and has-circ-0015164 (c); two circRNA structures were obtained from the CSCD database, red indicates bind position of miRNA, blue represents the position where the protein may bind, and green indicates open reading frame.

**Figure 10:**
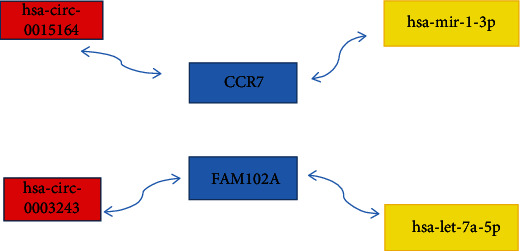
Constructing regulation network (a) circRNA-mRNA-miRNA network based on two mRNAs, two miRNAs, and two circRNAs by Cytoscape. Blue indicates mRNAs, yellow indicates miRNAs, and red indicates circRNAs.

circRNA has a more stable structure than other ncRNAs (noncoding RNAs) and acts as a miRNA sponge to competitively bind miRNA, thereby affecting the activity of miRNA and the expression of downstream target genes [27, 28]. We used two databases (circBase [29] and circBank [30]) to predict circRNAs of hsa-let-7a-5p and hsa-miR-1-3p. The overlapped targets (has-circ-0015164, hsa-circ-0003243) were selected for further analysis in Figure 9(a) and Supplement Files: Table S4. The basic structural pattern of these 2 circRNAs by the CSCD database [31] is shown in Figures 9(b) and 9(c) and Table 1. A ceRNA network of circRNA-mRNA-miRNA interaction through Cytoscape [32] was established and visualized (Figure 10(a)).

## 4. Discussion

Glioblastoma (GBM) is one of the most intractable tumors in neurosurgery because of its high recurrence rate and poor prognosis [33]. The traditional treatment of GBM is maximum surgical resection, supplemented by radiotherapy and chemotherapy. However, the five-year survival rate of patients is less than 5%, and the median survival time is only 15 months, which poses a serious burden on families and society [34]. Therefore, we urgently need a new diagnosis and treatment to improve the current situation.

Molecular diagnosis and targeted therapy have been given more and more attention when molecular biology and the in-depth study of the pathophysiological process of glioma have developed over the years [35]. The role of THE immune microenvironment in tumors cannot be ignored. Yang et al. found that by identifying the immune microenvironment in ovarian cancer, the high expression of immune-related genes is beneficial to the prognosis [36]. Wang et al. established immune-related prognosis scores based on 22 breast cancer cohorts, and patients with high scores had a good prognosis and chemotherapy effect [37]. Qi et al. found that members of the cGAS-STING pathway can be used as prognostic biomarkers and immunotherapy to target hepatocellular carcinoma patients [38]. For glioma, its molecular characteristics and molecular mechanism have become one of the research hotspots in recent years: the new classification of central nervous system tumors by the World Health Organization (WHO) breaks the traditional diagnostic principle based on histological criteria and includes molecular markers [39]. *IDH1* and *IDH2* mutations in patients with gliomas can be used as the basis for classification [40]; gliomas are classified into G1, G2, and G3 subtypes by the Chinese Glioma Genome Atlas (CGGA) [41]; gene mutations such as *TP53*, *PTEN*, and *H3K27M* and other gene mutations are also involved in the occurrence and development of gliomas [42, 43]. As the study progresses, it has been found that the pathology of glioma is similar to that of special cancers and that its mechanism is complex, which is possibly why advanced patients have a poor prognosis. Therefore, to find more effective diagnostic indicators and treatment targets of glioma is still the main goal.

Historically, most research has focused on genes that encode proteins. In recent years, it has been found that noncoding RNAs, such as microRNA (miRNA) and circular RNA (circRNA), although they do not encode proteins or only encode some small peptides, still play an important role in the regulation of physiological function and pathology [44, 45].

miRNA is a kind of noncoding single-stranded small molecule RNA, which regulates gene expression at the posttranscriptional level and is an important regulatory molecule in the process of life [46]. In tumor tissues, the expression of many miRNAs is significantly different and unique compared with the surrounding normal tissues, so the expression of miRNA can be used as a marker of tumor diagnosis, and miRNA technology will also become a powerful means of tumor prevention and treatment [47].

The expression of circRNAs is more stable than linear RNA, and its expression in vivo is more than 10 times that of linear RNA [48]. Its 3′ and 5′ ends are covalently linked by a closed-loop structure, so it is not easy to be affected by endonuclease and stable expression in vivo, so it is an ideal biomarker for disease diagnosis and molecular biotherapy target [49]. As a sponge of miRNA molecules, circRNA contains a large number of miRNA binding sites and acts as a miRNA sponge, which indirectly regulates the expression of downstream target genes of miRNA [50]. Current studies have shown that circRNA is abnormally expressed in many tumors, such as nonsmall cell lung cancer, colorectal cancer, breast cancer, liver cancer, and gastric cancer.

In the era of big data, it is possible for bioinformatics analysis to identify biomarkers with greater efficiency and accuracy that can be used as prognostic and treatment tools, so that gliomas can be more effectively detected and treated and patients can live longer, reducing recurrence and resistance to radiotherapy and chemotherapy [51]. The use of single-cell sequencing analysis and other technologies has also emerged as the times require, and related research has also emerged one after the other [52]. It has been found that tumor cells release RNA to the circulatory system through various types of microvesicles [8, 9]. Membrane vesicles secreted by cancer cells transfer tumor-derived RNA to platelet carriers. Glioblastoma transports microvesicles (exosomes) containing mRNA, miRNA, and angiogenic proteins, and messenger RNA mutants and miRNAs are detected in serum microvesicles of glioblastoma patients. It has been found that platelet-derived microvesicles can stimulate the proliferation of A549 cells and upregulate the expression of cyclin D2 [53, 54]. Platelet-derived microcapsules regulate metastasis and angiogenesis of lung cancer. It was found that the extracellular vesicles containing tumor DNA, miRNA, and protein in peripheral blood can be used as an information pool for the rapid diagnosis of typical gene mutations, regulatory miRNA, and tumor proteins in gliomas, and even to analyze the whole genome of brain tumors.

In general, glioma is diagnosed mainly by imaging examinations, and the final diagnosis is made by pathological examination of surgically resected specimens or tissue biopsy [55]. Body fluids such as blood are easy to collect, and the current research trend is towards efficient, sensitive, and noninvasive diagnostic methods, such as detecting gliomas through the appropriate molecular characteristics in peripheral blood and then popularizing early glioma screening. It can reduce the economic burden of the patient's family and society to a certain extent. The fact that platelets do not contain nuclei and that they can be easily separated from blood suggests that platelets obtained from tumor cells may become a new direction for cancer detection in the future.

At present, the mining of biomarkers related to tumor prognosis is mainly based on a molecular tag in mRNA, microRNA, and circRNA, while ignoring the interaction between circRNA-mRNA-miRNA and their interaction on tumor, and the regulatory network is less in the study of GBM. Therefore, this study will start with the related molecular characteristics of the circulatory system and nervous system to construct the ceRNA regulatory network and try to explore the potential relationship between it and GBM.

In this study, we obtained the platelet mRNA data (GSE31095) of normal persons and GBM patients from the GEO database. Two significantly different mRNAs (*CCR7* and *FAM102A*) were screened out by bioinformatics analysis. The analysis of the expression level of key genes in GBM patients and the overall survival rate showed that the expression of *CCR7* and *FAM102A* from platelets in GBM patients was also statistically significant.

Studies have found that *CCR7* is closely related to the prognosis of various cancers, such as squamous cell carcinoma of the tongue and floor of the mouth, bladder cancer, and colorectal cancer [56–58]. In addition, studies on glioblastoma have also reported that *CCR7* activates TGF-*β*1-induced invasion, migration, and epithelial-mesenchymal transformation of human malignant gliomas by activating MMP2/9 and the nuclear factor KappaB signal transduction pathway [59]. The current research report points out that *FAM102A* is mainly important for the formation of osteoclasts [60].

In order to clearly define the overlapping mRNA functions (upregulated and downregulated mRNAs) in GBM, we carried out gene enrichment analysis. GO enrichment analysis showed that 22 upregulated mRNAs mainly regulated the activity of immune synapses, serine peptides, and chimeric antigen receptor T (CAR T) cells; rapidly recruited particles to immune synapses; separated from target cells more quickly; and drove cytotoxicity. Heparin-stimulated serine protease was induced in gonadal tumors of Lobund Wistar rats [61]. The analysis of the KEGG pathway showed that this pathway was enriched in IL12-mediated signal transduction, ZAP70 to immune synaptic enzyme, second messenger molecule production, and TCR signal transduction and was also involved in many cancers. mRNAs also showed anticancer potential [62, 63].

It was found that CircCPA4 regulates the proliferation and metastasis of GBM. CircNTE can act as a sponge adsorbent of miR-422a and inhibit its activity, thus affecting the tumorigenesis of glioblastoma [64]. Although these studies found that circRNA is abnormally expressed in GBM and participates in tumor progression, the specific mechanism of its action in GBM is still unclear. We have identified two key circRNAs (hsa-circ-0015164, has-circ0003243) and constructed the structures of two circRNAs and found that these circRNA have not been studied, which may be a breakthrough for future research. Clarifying the biological function of circRNA may prove the molecular clues of GBM-targeted therapy, which is helpful for us to understand GBM at the genetic and molecular levels. Additionally, there were some limitations in this study: in recent years, multigroup and multiplatform integrated analyses, such as the GEO database and the TCGA database, have gained popularity. Our study also attempts to obtain the sequencing data of GBM through TCGA, GTEx, and other platforms. Unfortunately, there is a lack of normal samples on TCGA GBM. And if we include the normal samples corresponding to GBM in GTEx, the normal sample size will be too large and the tumor group samples will be too small. The purpose of this study is to explore the characteristics of gene expression in platelets and the differential expression between tumor groups and normal groups. To meet the tradeoff, we only analyze GSE31095 sequencing data on the GEO dataset, and we hope to have more comprehensive datasets or further verification in the future.

## 5. Conclusions

In this study, the GEO public database and bioinformatics analysis were used to analyze the expression data of GBM in platelets, and a ceRNA network was constructed to screen new potential diagnostic biomarkers, which provided a scientific basis for exploring the key analytical mechanism of GBM, and provided a new breakthrough for the development of new targets for early diagnosis and clinical treatment of GBM. However, the prognostic value of these important RNAs expressions still needs to be further confirmed.

## Figures and Tables

**Table 1:** Prediction of circRNAs.

circRNA	Gene name	Position	Strand	Genomic length	Best transcript
hsa_circ_0003243	DGUOK	chr2:74173845|74177859	+	4014	NM_080916
hsa_circ_0055268	DGUOK	chr2:74173845|74186088	+	12243	NM_080916
hsa_circ_0015164	SLC19A2	chr1:169446392|169446995	—	603	NM_006996
hsa_circ_0015165	SLC19A2	chr1:169446392|169455208	—	8816	NM_006996

## Data Availability

The datasets analyzed in this study are available in the Gene Expression Omnibus (GEO) public repository: (1) GEO: https://www.ncbi.nlm.nih.gov/geo/; (2) GEPIA: http://gepia.cancer-pku.cn/.

## Supplementary Materials

Supplementary 1 Supplement Files: Table S1: GSE31095 glioma raw data.

Supplementary 2 Supplement Files: Table S2: DEGs.

Supplementary 3 Supplement Files: Table S3: upregulated mRNAs and downregulated mRNAs.

Supplementary 4 Supplement Files: Table S4: the overlapped targets (has-circ-0015164, hsa-circ-0003243).
