# Supplementary material for: Identification of Potential Biomarkers of Platelet RNA in Glioblastoma by Bioinformatics Analysis
Source: Biomed Res Int. 2022 Aug 12;2022:2488139. doi: 10.1155/2022/2488139 (PMC9391609; doi:10.1155/2022/2488139)
Supplement: Supplementary 1 — Supplement Files: Table S1: GSE31095 glioma raw data. [file 2488139.f1.pdf]

Supplement Files: Table S1:GSE31095 glioma raw data

Accession	SAMPLE TYPE
GSM769920	healty control
GSM769921	healty control
GSM769922	healty control
GSM769923	healty control
GSM769924	healty control
GSM769925	healty control
GSM769926	healty control
GSM769927	healty control
GSM769928	healty control
GSM769929	healty control
GSM769930	healty control
GSM769931	healty control
GSM769932	brain tumor
GSM769933	brain tumor
GSM769934	brain tumor
GSM769935	brain tumor
GSM769936	brain tumor
GSM769937	brain tumor
GSM769938	brain tumor
GSM769939	brain tumor
