# Supplementary material for: Identification of Potential Biomarkers of Platelet RNA in Glioblastoma by Bioinformatics Analysis
Source: Biomed Res Int. 2022 Aug 12;2022:2488139. doi: 10.1155/2022/2488139 (PMC9391609; doi:10.1155/2022/2488139)
Supplement: Supplementary 2 — Supplement Files: Table S2: DEGs. [file 2488139.f2.pdf]

Supplement Files: Table S2:DEGs

Gene name	logFC	PValue	FDR	regulated
GIMAP7	2.354652	2.38E-07	0.004688	Up-Regulated
ADA	2.146243	2.12E-12	4.19E-08	Up-Regulated
LCK	2.430346	3.02E-09	5.96E-05	Up-Regulated
LTB	2.64917	5.58E-08	0.001101	Up-Regulated
GZMA	2.75205	2.91E-08	0.000573	Up-Regulated
GZMB	3.282743	3.94E-12	7.77E-08	Up-Regulated
GZMH	2.841013	6.99E-07	0.013762	Up-Regulated
ZAP70	2.312321	2.06E-06	0.04039	Up-Regulated
LEF1	2.404212	3.95E-07	0.007786	Up-Regulated
FAM102A	2.716688	3.62E-07	0.007119	Up-Regulated
CX3CR1	2.90767	8.27E-09	0.000163	Up-Regulated
NKG7	2.370286	1.35E-09	2.67E-05	Up-Regulated
KLRB1	2.405777	6.50E-13	1.28E-08	Up-Regulated
CDKN1C	2.493584	4.66E-14	9.20E-10	Up-Regulated
IL2RB	2.221076	2.41E-07	0.004739	Up-Regulated
RASAL3	2.366675	2.42E-06	0.047487	Up-Regulated
RPL34	2.050126	6.84E-07	0.013461	Up-Regulated
PLAC8	2.22022	1.05E-11	2.08E-07	Up-Regulated
FGFBP2	2.013025	1.58E-06	0.031109	Up-Regulated
CCL4	2.288887	7.48E-11	1.48E-06	Up-Regulated
CD3D	2.215982	2.10E-06	0.041305	Up-Regulated
CCR7	3.187991	1.88E-06	0.036879	Up-Regulated

FDXR	-2.13517	1.17E-11	2.32E-07	Down-Regulated
LTF	-2.01418	4.21E-07	0.008294	Down-Regulated
IL1R2	-3.96896	3.17E-10	6.25E-06	Down-Regulated
FKBP5	-2.72674	1.97E-17	3.90E-13	Down-Regulated
ACRC	-2.50518	7.47E-11	1.48E-06	Down-Regulated
DEFA4	-2.48737	3.52E-10	6.95E-06	Down-Regulated
DEFA3	-4.0208	1.44E-09	2.83E-05	Down-Regulated
CAMP	-2.1333	2.41E-07	0.004753	Down-Regulated
CST7	-3.7859	2.32E-07	0.004578	Down-Regulated
