# Supplementary material for: Identification of Potential Biomarkers of Platelet RNA in Glioblastoma by Bioinformatics Analysis
Source: Biomed Res Int. 2022 Aug 12;2022:2488139. doi: 10.1155/2022/2488139 (PMC9391609; doi:10.1155/2022/2488139)
Supplement: Supplementary 3 — Supplement Files: Table S3: upregulated mRNAs and downregulated mRNAs. [file 2488139.f3.pdf]

Supplement Files: Table S3:up-regulated mRNAs AND down-regulated mRNAs

Num	Gene ID	Gene name	Official Full Name	Location
1	168537	GIMAP7	GTPase, IMAP family member 7	7q36.1
2	100	ADA	adenosine deaminase	20q13.12
3	3932	LCK	LCK proto-oncogene, Src family tyrosine kinase	1p35.2
4	4050	LTB	lymphotoxin beta	6p21.33
5	3001	GZMA	granzyme A	5q11.2
6	3002	GZMB	granzyme B	14q12
7	2999	GZMH	granzyme H	14q12
8	7535	ZAP70	zeta chain of T cell receptor associated protein kinase 70	2q11.2
9	51176	LEF1	lymphoid enhancer binding factor 1	4q25
10	399665	FAM102A	family with sequence similarity 102 member A	9q34.11
11	1524	CX3CR1	C-X3-C motif chemokine receptor 1	3p22.2
12	4818	NKG7	natural killer cell granule protein 7	19q13.41
13	3820	KLRB1	killer cell lectin like receptor B1	12p13.31
14	1028	CDKN1C	cyclin dependent kinase inhibitor 1C	11p15.4
15	3560	IL2RB	interleukin 2 receptor subunit beta	22q12.3
16	64	RAS	RAS protein activator	19p

6	926	AL3	like 3	13.12
17	6164	RPL34	ribosomal protein L34	4q25
18	51316	PLAC8	placenta associated 8	4q21.22
19	83888	FGFBP2	fibroblast growth factor binding protein 2	4p15.32
20	6351	CCL4	C-C motif chemokine ligand 4	17q12
21	915	CD3D	CD3d molecule	11q23.3
22	1236	CCR7	C-C motif chemokine receptor 7	17q21.2
23	2232	FDXR	ferredoxin reductase	17q25.1
24	4057	LTF	lactotransferrin	3p21.31
25	7850	IL1R2	interleukin 1 receptor type 2	2q11.2
26	2289	FKBP5	FKBP prolyl isomerase 5	6p21.31
27	93953	ACRC	Acidic repeat-containing protein	Xq13.1
28	1669	DEFA4	defensin alpha 4	8p23.1
29	1668	DEFA3	defensin alpha 3	8p23.1
30	820	CAMP	cathelicidin antimicrobial peptide	3p21.31
31	8530	CST7	cystatin F	20p11.21
