# Supplementary material for: Identification of Potential Biomarkers of Platelet RNA in Glioblastoma by Bioinformatics Analysis
Source: Biomed Res Int. 2022 Aug 12;2022:2488139. doi: 10.1155/2022/2488139 (PMC9391609; doi:10.1155/2022/2488139)
Supplement: Supplementary 4 — Supplement Files: Table S4: the overlapped targets (has-circ-0015164, hsa-circ-0003243). [file 2488139.f4.pdf]

**Supplement Files: Table S4**:The overlapped targets (has-circ-0015164, hsa-circ-0003243)

| mirRNA | circBase prediction | | circBase prediction | | circBank prediction | | circBank prediction |
|---|---|---|---|---|---|---|---|
| hsa-miR-1-3p | | hsa-let-7a-5p | | hsa-miR-1-3p | | hsa-let-7a-5p | |
| | hsa_circ_0009298 | | hsa_circ_000828 | | hsa_circ_0043225 | | hsa_circ_0100108 |
| | hsa_circ_0009299 | | hsa_circ_0007211 | | hsa_circ_0006952 | | hsa_circ_0084831 |
| | hsa_circ_0009321 | | hsa_circ_0009180 | | hsa_circ_0120449 | | hsa_circ_0008534 |
| | hsa_circ_0009487 | | hsa_circ_0009181 | | hsa_circ_0056854 | | hsa_circ_0015491 |
| | hsa_circ_0009488 | | hsa_circ_0009255 | | hsa_circ_0000182 | | hsa_circ_0035477 |
| | hsa_circ_0009489 | | hsa_circ_0009301 | | hsa_circ_0001314 | | hsa_circ_0040524 |
| | hsa_circ_0009723 | | hsa_circ_0009300 | | hsa_circ_0001359 | | hsa_circ_0058229 |
| | hsa_circ_0009724 | | hsa_circ_002136 | | hsa_circ_0003243 | | hsa_circ_0069396 |
| | hsa_circ_0009725 | | hsa_circ_001621 | | hsa_circ_0016411 | | hsa_circ_0079284 |
| | hsa_circ_0009727 | | hsa_circ_0009316 | | hsa_circ_0016458 | | hsa_circ_0095454 |
| | hsa_circ_0 | | hsa_circ_0 | | hsa_circ_0 | | hsa_circ_0 |

| | | | |
|---|---|---|---|
| 009730 | 009318 | 017244 | 099132 |
| hsa_circ_0009731 | hsa_circ_0009315 | hsa_circ_0017248 | hsa_circ_0110572 |
| hsa_circ_0009733 | hsa_circ_0009420 | hsa_circ_0017924 | hsa_circ_0122027 |
| hsa_circ_0009734 | hsa_circ_0009419 | hsa_circ_0018274 | hsa_circ_0125513 |
| hsa_circ_0009735 | hsa_circ_0009487 | hsa_circ_0029985 | hsa_circ_0126187 |
| hsa_circ_0009736 | hsa_circ_0009488 | hsa_circ_0032157 | hsa_circ_0130623 |
| hsa_circ_0002437 | hsa_circ_0009489 | hsa_circ_0035431 | hsa_circ_0126186 |
| hsa_circ_0004493 | hsa_circ_0009551 | hsa_circ_0037010 | hsa_circ_0100106 |
| hsa_circ_0005553 | hsa_circ_0007962 | hsa_circ_0037157 | hsa_circ_0100105 |
| hsa_circ_0010439 | hsa_circ_0009684 | hsa_circ_0037158 | hsa_circ_0077806 |
| hsa_circ_0010442 | hsa_circ_0009687 | hsa_circ_0037953 | hsa_circ_0007652 |
| hsa_circ_0010443 | hsa_circ_0009689 | hsa_circ_0037956 | hsa_circ_0007035 |
| hsa_circ_0010444 | hsa_circ_0009690 | hsa_circ_0044896 | hsa_circ_0047885 |
| hsa_circ_000809 | hsa_circ_0009944 | hsa_circ_0048279 | hsa_circ_0001009 |

| | | | |
|---|---|---|---|
| hsa_circ_001228 | hsa_circ_0009945 | hsa_circ_0054702 | hsa_circ_0001377 |
| hsa_circ_0010450 | hsa_circ_0009946 | hsa_circ_0055849 | hsa_circ_0002233 |
| hsa_circ_0010451 | hsa_circ_0009948 | hsa_circ_0065182 | hsa_circ_0002383 |
| hsa_circ_0010452 | hsa_circ_0009949 | hsa_circ_0070330 | hsa_circ_0002984 |
| hsa_circ_0010453 | hsa_circ_0009952 | hsa_circ_0070429 | hsa_circ_0003243 |
| hsa_circ_0010785 | hsa_circ_0009953 | hsa_circ_0073111 | hsa_circ_0006315 |
| hsa_circ_0010786 | hsa_circ_0009957 | hsa_circ_0073114 | hsa_circ_0006552 |
| hsa_circ_0010787 | hsa_circ_0009958 | hsa_circ_0076220 | hsa_circ_0007767 |
| hsa_circ_0010789 | hsa_circ_0009959 | hsa_circ_0081233 | hsa_circ_0012628 |
| hsa_circ_0010792 | hsa_circ_0009960 | hsa_circ_0085081 | hsa_circ_0012792 |
| hsa_circ_0010791 | hsa_circ_0009961 | hsa_circ_0098246 | hsa_circ_0013751 |
| hsa_circ_0010904 | hsa_circ_0009962 | hsa_circ_0103391 | hsa_circ_0013761 |
| hsa_circ_0010915 | hsa_circ_0002898 | hsa_circ_0104944 | hsa_circ_0015262 |
| hsa_circ_00109 | hsa_circ_00091 | hsa_circ_010504 | hsa_circ_001798 |

| | | | |
|---|---|---|---|
| 17 | 18 | 2 | 3 |
| hsa_circ_0010918 | hsa_circ_0010040 | hsa_circ_0107203 | hsa_circ_0018280 |
| hsa_circ_0010919 | hsa_circ_0010041 | hsa_circ_0111674 | hsa_circ_0020083 |
| hsa_circ_0010921 | hsa_circ_0010042 | hsa_circ_0111675 | hsa_circ_0020919 |
| hsa_circ_0010922 | hsa_circ_0010043 | hsa_circ_0111817 | hsa_circ_0022023 |
| hsa_circ_0010983 | hsa_circ_0010044 | hsa_circ_0111906 | hsa_circ_0024842 |
| hsa_circ_0010984 | hsa_circ_0010045 | hsa_circ_0112774 | hsa_circ_0024843 |
| hsa_circ_0010985 | hsa_circ_000541 | hsa_circ_0112777 | hsa_circ_0027251 |
| hsa_circ_0011013 | hsa_circ_0010119 | hsa_circ_0112779 | hsa_circ_0028312 |
| hsa_circ_0011018 | hsa_circ_0010120 | hsa_circ_0112780 | hsa_circ_0029457 |
| hsa_circ_0011019 | hsa_circ_0010123 | hsa_circ_0112781 | hsa_circ_0029458 |
| hsa_circ_0011064 | hsa_circ_0010125 | hsa_circ_0114260 | hsa_circ_0030543 |
| hsa_circ_0011065 | hsa_circ_0010127 | hsa_circ_0114966 | hsa_circ_0030547 |
| hsa_circ_0011066 | hsa_circ_0010128 | hsa_circ_0116518 | hsa_circ_0032420 |
| hsa_c | hsa_c | hsa_c | hsa_c |

| | | | | |
|---|---|---|---|---|
| | irc_0011067 | irc_0010129 | irc_0116943 | irc_0035403 |
| | hsa_circ_0011068 | hsa_circ_0005240 | hsa_circ_0116959 | hsa_circ_0038074 |
| | hsa_circ_0011069 | hsa_circ_0008611 | hsa_circ_0120540 | hsa_circ_0038696 |
| | hsa_circ_0011070 | hsa_circ_0008109 | hsa_circ_0124295 | hsa_circ_0039012 |
| | hsa_circ_0011071 | hsa_circ_0006643 | hsa_circ_0126452 | hsa_circ_0040132 |
| | hsa_circ_0011178 | hsa_circ_0004784 | hsa_circ_0127820 | hsa_circ_0042098 |
| | hsa_circ_0011331 | hsa_circ_0004177 | hsa_circ_0128505 | hsa_circ_0047545 |
| | hsa_circ_0011332 | hsa_circ_0002978 | hsa_circ_0128904 | hsa_circ_0050570 |
| | hsa_circ_0011333 | hsa_circ_0008733 | hsa_circ_0128910 | hsa_circ_0054674 |
| | hsa_circ_0011334 | hsa_circ_0010170 | hsa_circ_0129699 | hsa_circ_0055402 |
| | hsa_circ_0006349 | hsa_circ_0010171 | hsa_circ_0132492 | hsa_circ_0059945 |
| | hsa_circ_0011344 | hsa_circ_0010172 | hsa_circ_0132843 | hsa_circ_0063475 |
| | hsa_circ_0011416 | hsa_circ_0010246 | hsa_circ_0133348 | hsa_circ_0063972 |
| | hsa_circ_0011417 | hsa_circ_0010250 | hsa_circ_0134161 | hsa_circ_0065305 |

| | | | |
|---|---|---|---|
| hsa_circ_0011449 | hsa_circ_0010251 | hsa_circ_0134163 | hsa_circ_0065552 |
| hsa_circ_0011453 | hsa_circ_0010252 | hsa_circ_0137646 | hsa_circ_0066148 |
| hsa_circ_0011583 | hsa_circ_0010253 | hsa_circ_0136084 | hsa_circ_0066856 |
| hsa_circ_0011617 | hsa_circ_0010254 | hsa_circ_0132627 | hsa_circ_0069243 |
| hsa_circ_0011623 | hsa_circ_0010255 | hsa_circ_0122779 | hsa_circ_0069443 |
| hsa_circ_0011661 | hsa_circ_0010256 | hsa_circ_0118441 | hsa_circ_0071021 |
| hsa_circ_0011662 | hsa_circ_0010258 | hsa_circ_0114588 | hsa_circ_0071023 |
| hsa_circ_0011663 | hsa_circ_0010245 | hsa_circ_0106689 | hsa_circ_0071160 |
| hsa_circ_0011664 | hsa_circ_0010399 | hsa_circ_0104672 | hsa_circ_0071365 |
| hsa_circ_0011671 | hsa_circ_0010403 | hsa_circ_0097401 | hsa_circ_0071818 |
| hsa_circ_0011687 | hsa_circ_0010402 | hsa_circ_0076704 | hsa_circ_0088153 |
| hsa_circ_0011744 | hsa_circ_0010553 | hsa_circ_0069337 | hsa_circ_0090614 |
| hsa_circ_0011745 | hsa_circ_0010559 | hsa_circ_0067913 | hsa_circ_0092556 |
| hsa_circ_00117 | hsa_circ_00105 | hsa_circ_006790 | hsa_circ_009431 |

| | | | |
|---|---|---|---|
| 46 | 60 | 9 | 6 |
| hsa_circ_0011748 | hsa_circ_0010570 | hsa_circ_0064604 | hsa_circ_0094964 |
| hsa_circ_0011749 | hsa_circ_0010573 | hsa_circ_0063059 | hsa_circ_0095790 |
| hsa_circ_0011952 | hsa_circ_0010574 | hsa_circ_0057034 | hsa_circ_0095791 |
| hsa_circ_0012016 | hsa_circ_0010551 | hsa_circ_0044898 | hsa_circ_0098180 |
| hsa_circ_0012017 | hsa_circ_0005247 | hsa_circ_0042421 | hsa_circ_0099045 |
| hsa_circ_0012020 | hsa_circ_0010869 | hsa_circ_0039359 | hsa_circ_0099128 |
| hsa_circ_0012021 | hsa_circ_00040 | hsa_circ_0039357 | hsa_circ_0099201 |
| hsa_circ_0012093 | hsa_circ_0010888 | hsa_circ_0025708 | hsa_circ_0099755 |
| hsa_circ_0012223 | hsa_circ_0003553 | hsa_circ_0017246 | hsa_circ_0102445 |
| hsa_circ_0012281 | hsa_circ_0010948 | hsa_circ_0015164 | hsa_circ_0104465 |
| hsa_circ_0012285 | hsa_circ_0010954 | hsa_circ_0014446 | hsa_circ_0105287 |
| hsa_circ_0012290 | hsa_circ_0010957 | hsa_circ_0007478 | hsa_circ_0107665 |
| hsa_circ_0012291 | hsa_circ_0010960 | hsa_circ_0005228 | hsa_circ_0107710 |
| hsa_c | hsa_c | hsa_c | hsa_c |

| | | | |
|---|---|---|---|
| irc_0012292 | irc_0011023 | irc_0001360 | irc_0114399 |
| hsa_circ_0012467 | hsa_circ_0011024 | | hsa_circ_0114983 |
| hsa_circ_0012490 | hsa_circ_0007283 | | hsa_circ_0115837 |
| hsa_circ_0012491 | hsa_circ_0011048 | | hsa_circ_0116603 |
| hsa_circ_0012496 | hsa_circ_0011109 | | hsa_circ_0117451 |
| hsa_circ_0012497 | hsa_circ_0011110 | | hsa_circ_0117792 |
| hsa_circ_0012498 | hsa_circ_0008057 | | hsa_circ_0123431 |
| hsa_circ_0012499 | hsa_circ_0011111 | | hsa_circ_0123441 |
| hsa_circ_0012504 | hsa_circ_0011116 | | hsa_circ_0124202 |
| hsa_circ_0012505 | hsa_circ_0011117 | | hsa_circ_0126300 |
| hsa_circ_0012506 | hsa_circ_0011118 | | hsa_circ_0128770 |
| hsa_circ_0012507 | hsa_circ_0011119 | | hsa_circ_0128793 |
| hsa_circ_0012666 | hsa_circ_0007895 | | hsa_circ_0129760 |
| hsa_circ_0012667 | hsa_circ_0011125 | | hsa_circ_0130220 |

| | | | |
|---|---|---|---|
| hsa_circ_0012669 | hsa_circ_0011137 | | hsa_circ_0132958 |
| hsa_circ_0012670 | hsa_circ_0011138 | | hsa_circ_0133128 |
| hsa_circ_0012671 | hsa_circ_0011139 | | hsa_circ_0135190 |
| hsa_circ_0012863 | hsa_circ_0011140 | | hsa_circ_0139311 |
| hsa_circ_0012963 | hsa_circ_0011141 | | hsa_circ_0139779 |
| hsa_circ_0012988 | hsa_circ_0011136 | | hsa_circ_0140179 |
| hsa_circ_0013014 | hsa_circ_0005700 | | hsa_circ_0140177 |
| hsa_circ_0013015 | hsa_circ_0011192 | | hsa_circ_0134144 |
| hsa_circ_0013016 | hsa_circ_0011193 | | hsa_circ_0132957 |
| hsa_circ_0013017 | hsa_circ_0011194 | | hsa_circ_0126380 |
| hsa_circ_0013086 | hsa_circ_0011196 | | hsa_circ_0124377 |
| hsa_circ_0013118 | hsa_circ_0011197 | | hsa_circ_0114987 |
| hsa_circ_0013129 | hsa_circ_0003904 | | hsa_circ_0114985 |
| hsa_circ_00131 | hsa_circ_00112 | | hsa_circ_011108 |

| | | |
|---|---|---|
| 30 | 06 | 3 |
| hsa_circ_0013131 | hsa_circ_0011207 | hsa_circ_0110609 |
| hsa_circ_0013146 | hsa_circ_0011208 | hsa_circ_0108473 |
| hsa_circ_0013147 | hsa_circ_0011209 | hsa_circ_0106200 |
| hsa_circ_0013210 | hsa_circ_0011210 | hsa_circ_0105502 |
| hsa_circ_0013243 | hsa_circ_0011205 | hsa_circ_0104521 |
| hsa_circ_0013303 | hsa_circ_0011212 | hsa_circ_0102436 |
| hsa_circ_0013306 | hsa_circ_0011354 | hsa_circ_0096512 |
| hsa_circ_0013307 | hsa_circ_0011360 | hsa_circ_0095928 |
| hsa_circ_0007868 | hsa_circ_0011364 | hsa_circ_0095381 |
| hsa_circ_0006695 | hsa_circ_0011413 | hsa_circ_0095056 |
| hsa_circ_0013458 | hsa_circ_0011417 | hsa_circ_0084721 |
| hsa_circ_0013459 | hsa_circ_0011416 | hsa_circ_0083905 |
| hsa_circ_0013529 | hsa_circ_0011451 | hsa_circ_0081202 |
| hsa_c | hsa_c | hsa_c |

| | | |
|---|---|---|
| irc_0013534 | irc_0011450 | irc_0079273 |
| hsa_circ_0013535 | hsa_circ_0011449 | hsa_circ_0077388 |
| hsa_circ_0013538 | hsa_circ_0011454 | hsa_circ_0069244 |
| hsa_circ_0013539 | hsa_circ_0011474 | hsa_circ_0060918 |
| hsa_circ_0013545 | hsa_circ_0004320 | hsa_circ_0060243 |
| hsa_circ_0013552 | hsa_circ_0011521 | hsa_circ_0055250 |
| hsa_circ_0013573 | hsa_circ_0011523 | hsa_circ_0054127 |
| hsa_circ_0013575 | hsa_circ_0011524 | hsa_circ_0053375 |
| hsa_circ_0013576 | hsa_circ_0003730 | hsa_circ_0050115 |
| hsa_circ_0013579 | hsa_circ_0011559 | hsa_circ_0036173 |
| hsa_circ_0013581 | hsa_circ_0011560 | hsa_circ_0034654 |
| hsa_circ_0013583 | hsa_circ_0011617 | hsa_circ_0032419 |
| hsa_circ_0013584 | hsa_circ_0011623 | hsa_circ_0026782 |
| hsa_circ_0013585 | hsa_circ_0011671 | hsa_circ_0025996 |

| | | | |
|---|---|---|---|
| hsa_circ_0013586 | hsa_circ_0011687 | | hsa_circ_0020127 |
| hsa_circ_0013605 | hsa_circ_0011711 | | hsa_circ_0017608 |
| hsa_circ_0013628 | hsa_circ_0011713 | | hsa_circ_0015164 |
| hsa_circ_0013638 | hsa_circ_0011715 | | hsa_circ_0014084 |
| hsa_circ_0013681 | hsa_circ_0011716 | | hsa_circ_0013760 |
| hsa_circ_0013682 | hsa_circ_0011753 | | hsa_circ_0005435 |
| hsa_circ_0013684 | hsa_circ_0011754 | | hsa_circ_0005368 |
| hsa_circ_0013685 | hsa_circ_0011755 | | hsa_circ_0005229 |
| hsa_circ_0013687 | hsa_circ_0011756 | | hsa_circ_0004300 |
| hsa_circ_0013688 | hsa_circ_0011769 | | hsa_circ_0003806 |
| hsa_circ_0013689 | hsa_circ_0011770 | | hsa_circ_0003369 |
| hsa_circ_0013692 | hsa_circ_0011771 | | hsa_circ_0000676 |
| hsa_circ_0013749 | hsa_circ_0011772 | | hsa_circ_0022382 |
| hsa_circ_00137 | hsa_circ_00117 | | hsa_circ_008343 |

| | | |
|---|---|---|
| 50 | 73 | 2 |
| hsa_circ_0013751 | hsa_circ_0011774 | hsa_circ_0102446 |
| hsa_circ_0013752 | hsa_circ_0011775 | |
| hsa_circ_0005737 | hsa_circ_0011777 | |
| hsa_circ_0006744 | hsa_circ_0011778 | |
| hsa_circ_0007526 | hsa_circ_0011779 | |
| hsa_circ_0008663 | hsa_circ_0011849 | |
| hsa_circ_0013785 | hsa_circ_0011850 | |
| hsa_circ_0013791 | hsa_circ_0011851 | |
| hsa_circ_0013796 | hsa_circ_0011852 | |
| hsa_circ_0013792 | hsa_circ_0011899 | |
| hsa_circ_0013794 | hsa_circ_0011917 | |
| hsa_circ_0013795 | hsa_circ_0011927 | |
| hsa_circ_0013800 | hsa_circ_0012010 | |
| hsa_c | hsa_c | |

| | |
|---|---|
| irc_0013828 | irc_0012032 |
| hsa_circ_0013829 | hsa_circ_0012034 |
| hsa_circ_0004784 | hsa_circ_0012035 |
| hsa_circ_0006643 | hsa_circ_0012040 |
| hsa_circ_0002978 | hsa_circ_0012042 |
| hsa_circ_0013860 | hsa_circ_0012043 |
| hsa_circ_0002956 | hsa_circ_0012044 |
| hsa_circ_0004051 | hsa_circ_0012105 |
| hsa_circ_0013861 | hsa_circ_0012180 |
| hsa_circ_0005431 | hsa_circ_0012222 |
| hsa_circ_0013862 | hsa_circ_0012330 |
| hsa_circ_0013864 | hsa_circ_0012331 |
| hsa_circ_0013865 | hsa_circ_0012327 |
| hsa_circ_0013866 | hsa_circ_0008197 |

| | |
|---|---|
| hsa_circ_0013867 | hsa_circ_0012391 |
| hsa_circ_0013892 | hsa_circ_0012393 |
| hsa_circ_0013893 | hsa_circ_0012394 |
| hsa_circ_0013894 | hsa_circ_0012395 |
| hsa_circ_0013897 | hsa_circ_0012396 |
| hsa_circ_0013901 | hsa_circ_0012397 |
| hsa_circ_0013903 | hsa_circ_0012398 |
| hsa_circ_0003953 | hsa_circ_0012399 |
| hsa_circ_0007359 | hsa_circ_0012400 |
| hsa_circ_0007636 | hsa_circ_0012531 |
| hsa_circ_0008442 | hsa_circ_0012532 |
| hsa_circ_0013934 | hsa_circ_0012537 |
| hsa_circ_0013935 | hsa_circ_0012538 |
| hsa_circ_00139 | hsa_circ_00125 |

| | |
|---|---|
| 36 | 39 |
| hsa_circ_0013937 | hsa_circ_0012541 |
| hsa_circ_0014028 | hsa_circ_0012543 |
| hsa_circ_0014043 | hsa_circ_0012544 |
| hsa_circ_0014045 | hsa_circ_0012588 |
| hsa_circ_0014106 | hsa_circ_0006676 |
| hsa_circ_0014246 | hsa_circ_0012600 |
| hsa_circ_0014247 | hsa_circ_0012601 |
| hsa_circ_0014248 | hsa_circ_0012602 |
| hsa_circ_0014249 | hsa_circ_0012603 |
| hsa_circ_0014250 | hsa_circ_0012604 |
| hsa_circ_0014251 | hsa_circ_0012605 |
| hsa_circ_0014252 | hsa_circ_0012606 |
| hsa_circ_0014253 | hsa_circ_0012607 |
| hsa_c | hsa_c |

| | |
|---|---|
| irc_0 0142 54 | irc_0 0126 26 |
| hsa_c irc_0 0142 55 | hsa_c irc_0 0126 27 |
| hsa_c irc_0 0143 22 | hsa_c irc_0 0126 28 |
| hsa_c irc_0 0143 23 | hsa_c irc_0 0126 29 |
| hsa_c irc_0 0143 24 | hsa_c irc_0 0126 30 |
| hsa_c irc_0 0143 25 | hsa_c irc_0 0126 66 |
| hsa_c irc_0 0143 26 | hsa_c irc_0 0126 67 |
| hsa_c irc_0 0143 27 | hsa_c irc_0 0126 68 |
| hsa_c irc_0 0143 28 | hsa_c irc_0 0126 69 |
| hsa_c irc_0 0143 29 | hsa_c irc_0 0126 70 |
| hsa_c irc_0 0143 30 | hsa_c irc_0 0126 84 |
| hsa_c irc_0 0143 31 | hsa_c irc_0 0126 89 |
| hsa_c irc_0 0143 32 | hsa_c irc_0 0126 91 |
| hsa_c irc_0 0143 33 | hsa_c irc_0 0126 94 |

| | |
|---|---|
| hsa_circ_0014407 | hsa_circ_0012696 |
| hsa_circ_0014431 | hsa_circ_0012698 |
| hsa_circ_0014853 | hsa_circ_0012699 |
| hsa_circ_0014854 | hsa_circ_0012700 |
| hsa_circ_0014855 | hsa_circ_0012703 |
| hsa_circ_0014856 | hsa_circ_0012762 |
| hsa_circ_0014857 | hsa_circ_0012801 |
| hsa_circ_0008857 | hsa_circ_0012803 |
| hsa_circ_0015017 | hsa_circ_0012804 |
| hsa_circ_0015018 | hsa_circ_0012807 |
| hsa_circ_0015020 | hsa_circ_0012808 |
| hsa_circ_0015025 | hsa_circ_0012809 |
| hsa_circ_0015028 | hsa_circ_0012810 |
| hsa_circ_00150 | hsa_circ_00128 |

| | |
|---|---|
| 29 | 41 |
| hsa_circ_0015030 | hsa_circ_001483 |
| hsa_circ_0015031 | hsa_circ_0012863 |
| hsa_circ_0015033 | hsa_circ_0012923 |
| hsa_circ_0015164 | hsa_circ_0012931 |
| hsa_circ_0015165 | hsa_circ_0013047 |
| hsa_circ_0015166 | hsa_circ_0013048 |
| hsa_circ_0015339 | hsa_circ_0013049 |
| hsa_circ_0015418 | hsa_circ_0013053 |
| hsa_circ_0015458 | hsa_circ_0013054 |
| hsa_circ_0015496 | hsa_circ_0013055 |
| hsa_circ_0015497 | hsa_circ_0013076 |
| hsa_circ_0015499 | hsa_circ_0013077 |
| hsa_circ_0015500 | hsa_circ_0013078 |
| hsa_c | hsa_c |

| | |
|---|---|
| irc_0015502 | irc_0013079 |
| hsa_circ_0015503 | hsa_circ_0013080 |
| hsa_circ_0003683 | hsa_circ_0013125 |
| hsa_circ_0015522 | hsa_circ_0013129 |
| hsa_circ_0015610 | hsa_circ_0013130 |
| hsa_circ_0015618 | hsa_circ_0013131 |
| hsa_circ_0015619 | hsa_circ_0005720 |
| hsa_circ_0015621 | hsa_circ_001732 |
| hsa_circ_0015622 | hsa_circ_0013308 |
| hsa_circ_0015623 | hsa_circ_0013344 |
| hsa_circ_0015624 | hsa_circ_0013367 |
| hsa_circ_0015625 | hsa_circ_0013415 |
| hsa_circ_0015626 | hsa_circ_0013417 |
| hsa_circ_0015627 | hsa_circ_0013418 |

| | |
|---|---|
| hsa_circ_0015681 | hsa_circ_0013419 |
| hsa_circ_0015682 | hsa_circ_0013420 |
| hsa_circ_0015684 | hsa_circ_0013421 |
| hsa_circ_0015686 | hsa_circ_0013425 |
| hsa_circ_0015832 | hsa_circ_0013427 |
| hsa_circ_0015894 | hsa_circ_0013428 |
| hsa_circ_0015896 | hsa_circ_0013508 |
| hsa_circ_0015897 | hsa_circ_0013509 |
| hsa_circ_0015898 | hsa_circ_0013510 |
| hsa_circ_0015899 | hsa_circ_0013511 |
| hsa_circ_0015920 | hsa_circ_0013512 |
| hsa_circ_0015930 | hsa_circ_0013513 |
| hsa_circ_0015937 | hsa_circ_0013545 |
| hsa_circ_00159 | hsa_circ_00136 |

| | |
|---|---|
| 38 | 05 |
| hsa_circ_0015946 | hsa_circ_0013606 |
| hsa_circ_0015947 | hsa_circ_0013627 |
| hsa_circ_0015948 | hsa_circ_0013641 |
| hsa_circ_0015949 | hsa_circ_0013656 |
| hsa_circ_0015950 | hsa_circ_0013651 |
| hsa_circ_0015971 | hsa_circ_0013659 |
| hsa_circ_0016130 | hsa_circ_0002233 |
| hsa_circ_0016179 | hsa_circ_0013749 |
| hsa_circ_0016180 | hsa_circ_0013750 |
| hsa_circ_0016202 | hsa_circ_0013751 |
| hsa_circ_0016203 | hsa_circ_0013752 |
| hsa_circ_0016218 | hsa_circ_0013753 |
| hsa_circ_0016421 | hsa_circ_0013760 |
| hsa_c | hsa_c |

| | |
|---|---|
| irc_0016423 | irc_0013761 |
| hsa_circ_0016440 | hsa_circ_0013762 |
| hsa_circ_0016480 | hsa_circ_0013765 |
| hsa_circ_0016483 | hsa_circ_0013806 |
| hsa_circ_0016484 | hsa_circ_0013800 |
| hsa_circ_0016524 | hsa_circ_0004434 |
| hsa_circ_0003163 | hsa_circ_0013829 |
| hsa_circ_0016897 | hsa_circ_0002978 |
| hsa_circ_0016999 | hsa_circ_0004784 |
| hsa_circ_0017177 | hsa_circ_0006643 |
| hsa_circ_0017195 | hsa_circ_0013831 |
| hsa_circ_0017218 | hsa_circ_0013832 |
| hsa_circ_0017221 | hsa_circ_0013833 |
| hsa_circ_0017222 | hsa_circ_0013834 |

| | |
|---|---|
| hsa_circ_0017223 | hsa_circ_0013835 |
| hsa_circ_0017224 | hsa_circ_0013836 |
| hsa_circ_0017225 | hsa_circ_0013837 |
| hsa_circ_0017226 | hsa_circ_0013828 |
| hsa_circ_0017227 | hsa_circ_0013861 |
| hsa_circ_0002240 | hsa_circ_0013864 |
| hsa_circ_0017242 | hsa_circ_0013865 |
| hsa_circ_0017243 | hsa_circ_0013867 |
| hsa_circ_0017244 | hsa_circ_0013887 |
| hsa_circ_0017245 | hsa_circ_0003953 |
| hsa_circ_0017246 | hsa_circ_0007359 |
| hsa_circ_0017247 | hsa_circ_0007636 |
| hsa_circ_0017248 | hsa_circ_0008442 |
| hsa_circ_00146 | hsa_circ_00139 |

| | |
|---|---|
| 9 | 56 |
| hsa_circ_0052432 | hsa_circ_0013934 |
| hsa_circ_0052433 | hsa_circ_0013935 |
| hsa_circ_0052434 | hsa_circ_0013936 |
| hsa_circ_0052435 | hsa_circ_0013937 |
| hsa_circ_0052436 | hsa_circ_0013942 |
| hsa_circ_0052437 | hsa_circ_0006061 |
| hsa_circ_0052438 | hsa_circ_0006679 |
| hsa_circ_0052439 | hsa_circ_0013974 |
| hsa_circ_0052440 | hsa_circ_0013976 |
| hsa_circ_0052441 | hsa_circ_0013978 |
| hsa_circ_0052484 | hsa_circ_0004300 |
| hsa_circ_0052538 | hsa_circ_0013997 |
| hsa_circ_0052542 | hsa_circ_0013998 |
| hsa_c | hsa_c |

| | |
|---|---|
| irc_0052543 | irc_0013999 |
| hsa_circ_0052546 | hsa_circ_0014055 |
| hsa_circ_0052547 | hsa_circ_0014062 |
| hsa_circ_0052548 | hsa_circ_0014067 |
| hsa_circ_0052549 | hsa_circ_0014070 |
| hsa_circ_0052550 | hsa_circ_0014072 |
| hsa_circ_0052551 | hsa_circ_0014073 |
| hsa_circ_0052679 | hsa_circ_0014075 |
| hsa_circ_0052680 | hsa_circ_0014084 |
| hsa_circ_0052882 | hsa_circ_0014085 |
| hsa_circ_0052883 | hsa_circ_0006817 |
| hsa_circ_0052884 | hsa_circ_0014243 |
| hsa_circ_0052885 | hsa_circ_0014284 |
| hsa_circ_0052891 | hsa_circ_0014311 |

| | |
|---|---|
| hsa_circ_0052886 | hsa_circ_0014342 |
| hsa_circ_0052887 | hsa_circ_0014330 |
| hsa_circ_0052888 | hsa_circ_0014407 |
| hsa_circ_0052889 | hsa_circ_0014469 |
| hsa_circ_0052890 | hsa_circ_0014467 |
| hsa_circ_0053176 | hsa_circ_0014500 |
| hsa_circ_0053179 | hsa_circ_0014509 |
| hsa_circ_0053192 | hsa_circ_0014516 |
| hsa_circ_0053197 | hsa_circ_0014517 |
| hsa_circ_0053204 | hsa_circ_0014518 |
| hsa_circ_0053205 | hsa_circ_0014519 |
| hsa_circ_0053206 | hsa_circ_0014515 |
| hsa_circ_0053207 | hsa_circ_0014546 |
| hsa_circ_00532 | hsa_circ_00146 |

| | |
|---|---|
| 10 | 67 |
| hsa_circ_053211 | hsa_circ_014666 |
| hsa_circ_053379 | hsa_circ_014676 |
| hsa_circ_053384 | hsa_circ_014686 |
| hsa_circ_053385 | hsa_circ_014687 |
| hsa_circ_053386 | hsa_circ_014688 |
| hsa_circ_053387 | hsa_circ_014689 |
| hsa_circ_053388 | hsa_circ_014690 |
| hsa_circ_053389 | hsa_circ_014691 |
| hsa_circ_053390 | hsa_circ_014685 |
| hsa_circ_007337 | hsa_circ_014703 |
| hsa_circ_053433 | hsa_circ_014704 |
| hsa_circ_053434 | hsa_circ_014705 |
| hsa_circ_053435 | hsa_circ_014702 |
| hsa_c | hsa_c |

| | |
|---|---|
| irc_0053436 | irc_0014736 |
| hsa_circ_0053437 | hsa_circ_0014771 |
| hsa_circ_0053438 | hsa_circ_0014772 |
| hsa_circ_0053442 | hsa_circ_0014770 |
| hsa_circ_0053443 | hsa_circ_0014806 |
| hsa_circ_0053444 | hsa_circ_0014807 |
| hsa_circ_002146 | hsa_circ_0014808 |
| hsa_circ_001287 | hsa_circ_0014809 |
| hsa_circ_0053859 | hsa_circ_0014810 |
| hsa_circ_0053860 | hsa_circ_0014811 |
| hsa_circ_0053861 | hsa_circ_0014805 |
| hsa_circ_0053862 | hsa_circ_0014861 |
| hsa_circ_0053863 | hsa_circ_0014860 |
| hsa_circ_0053864 | hsa_circ_0014868 |

| | |
|---|---|
| hsa_circ_0002735 | hsa_circ_0014869 |
| hsa_circ_0005910 | hsa_circ_0014867 |
| hsa_circ_0053889 | hsa_circ_0014880 |
| hsa_circ_0053890 | hsa_circ_0015097 |
| hsa_circ_0053892 | hsa_circ_0015099 |
| hsa_circ_0053897 | hsa_circ_0015096 |
| hsa_circ_0053893 | hsa_circ_0015165 |
| hsa_circ_0053894 | hsa_circ_0015164 |
| hsa_circ_0053895 | hsa_circ_0008003 |
| hsa_circ_0053896 | hsa_circ_0015199 |
| hsa_circ_0006004 | hsa_circ_0015204 |
| hsa_circ_0006746 | hsa_circ_0015212 |
| hsa_circ_0053944 | hsa_circ_0015213 |
| hsa_circ_00539 | hsa_circ_00152 |

| | |
|---|---|
| 45 | 14 |
| hsa_circ_0053946 | hsa_circ_0015222 |
| hsa_circ_0054110 | hsa_circ_0015223 |
| hsa_circ_0054168 | hsa_circ_0015226 |
| hsa_circ_0054181 | hsa_circ_0015218 |
| hsa_circ_0054182 | hsa_circ_0002269 |
| hsa_circ_0054269 | hsa_circ_0015259 |
| hsa_circ_0054271 | hsa_circ_0015260 |
| hsa_circ_0054275 | hsa_circ_0015261 |
| hsa_circ_0054278 | hsa_circ_0015262 |
| hsa_circ_0054279 | hsa_circ_0015263 |
| hsa_circ_0054282 | hsa_circ_0015258 |
| hsa_circ_0054283 | hsa_circ_01903 |
| hsa_circ_0054285 | hsa_circ_0015275 |
| hsa_c | hsa_c |

| | |
|---|---|
| irc_0054293 | irc_0015273 |
| hsa_circ_0054297 | hsa_circ_0015282 |
| hsa_circ_0054299 | hsa_circ_0015315 |
| hsa_circ_0054300 | hsa_circ_0015317 |
| hsa_circ_0054301 | hsa_circ_0015312 |
| hsa_circ_0054302 | hsa_circ_0015474 |
| hsa_circ_0054303 | hsa_circ_0015490 |
| hsa_circ_0054304 | hsa_circ_0015491 |
| hsa_circ_0054305 | hsa_circ_0007652 |
| hsa_circ_0054306 | hsa_circ_0015510 |
| hsa_circ_0005539 | hsa_circ_0015511 |
| hsa_circ_0054333 | hsa_circ_0015512 |
| hsa_circ_0054334 | hsa_circ_0015513 |
| hsa_circ_0054335 | hsa_circ_0015514 |

| | |
|---|---|
| hsa_circ_0054336 | hsa_circ_0015509 |
| hsa_circ_0054337 | hsa_circ_0015545 |
| hsa_circ_0054338 | hsa_circ_0015681 |
| hsa_circ_0054339 | hsa_circ_0015682 |
| hsa_circ_0054340 | hsa_circ_0015683 |
| hsa_circ_0054341 | hsa_circ_0015684 |
| hsa_circ_0054436 | hsa_circ_0015815 |
| hsa_circ_0054537 | hsa_circ_0015814 |
| hsa_circ_0054538 | hsa_circ_0015946 |
| hsa_circ_0054552 | hsa_circ_0015995 |
| hsa_circ_0054721 | hsa_circ_0016037 |
| hsa_circ_0054722 | hsa_circ_0016055 |
| hsa_circ_0054726 | hsa_circ_0016054 |
| hsa_circ_00547 | hsa_circ_00160 |

| | |
|---|---|
| 28 | 58 |
| hsa_circ_0054731 | hsa_circ_0016056 |
| hsa_circ_0054734 | hsa_circ_0016106 |
| hsa_circ_0054735 | hsa_circ_0016107 |
| hsa_circ_0054736 | hsa_circ_0016108 |
| hsa_circ_0054737 | hsa_circ_0007684 |
| hsa_circ_0054741 | hsa_circ_0016126 |
| hsa_circ_0002370 | hsa_circ_0016127 |
| hsa_circ_0006759 | hsa_circ_0016154 |
| hsa_circ_0054924 | hsa_circ_0016155 |
| hsa_circ_0054944 | hsa_circ_0016146 |
| hsa_circ_0054949 | hsa_circ_000079 |
| hsa_circ_0054950 | hsa_circ_0016156 |
| hsa_circ_0054951 | hsa_circ_0016160 |
| hsa_c | hsa_c |

| | |
|---|---|
| irc_0005266 | irc_0016162 |
| hsa_circ_0003808 | hsa_circ_0016164 |
| hsa_circ_0055039 | hsa_circ_0016165 |
| hsa_circ_0055040 | hsa_circ_0016166 |
| hsa_circ_0055045 | hsa_circ_0016167 |
| hsa_circ_0055046 | hsa_circ_0016208 |
| hsa_circ_0055047 | hsa_circ_0016230 |
| hsa_circ_0055048 | hsa_circ_0016238 |
| hsa_circ_0055049 | hsa_circ_0016241 |
| hsa_circ_0055050 | hsa_circ_0016240 |
| hsa_circ_0055052 | hsa_circ_0016237 |
| hsa_circ_0055069 | hsa_circ_0016275 |
| hsa_circ_0055070 | hsa_circ_0016276 |
| hsa_circ_0055071 | hsa_circ_0016277 |

| | |
|---|---|
| hsa_circ_0055072 | hsa_circ_0016274 |
| hsa_circ_0055101 | hsa_circ_0016279 |
| hsa_circ_0055147 | hsa_circ_0016280 |
| hsa_circ_0055230 | hsa_circ_0016281 |
| hsa_circ_0055231 | hsa_circ_0016282 |
| hsa_circ_0055234 | hsa_circ_0016278 |
| hsa_circ_0055236 | hsa_circ_0016310 |
| hsa_circ_0055237 | hsa_circ_0016339 |
| hsa_circ_0055238 | hsa_circ_0016342 |
| hsa_circ_0055240 | hsa_circ_0016338 |
| hsa_circ_0055251 | hsa_circ_0016376 |
| hsa_circ_0003243 | hsa_circ_0016389 |
| hsa_circ_0055268 | hsa_circ_0007655 |
| hsa_circ_00552 | hsa_circ_00164 |

| | |
|---|---|
| 70 | 40 |
| hsa_circ_0055271 | hsa_circ_0016537 |
| hsa_circ_0055272 | hsa_circ_0016534 |
| hsa_circ_0055414 | hsa_circ_0003163 |
| hsa_circ_0055423 | hsa_circ_0016543 |
| hsa_circ_0055425 | hsa_circ_0016585 |
| hsa_circ_0007191 | hsa_circ_0016584 |
| hsa_circ_0007490 | hsa_circ_0016637 |
| hsa_circ_0055476 | hsa_circ_0016638 |
| hsa_circ_0055482 | hsa_circ_0016639 |
| hsa_circ_0055483 | hsa_circ_0016640 |
| hsa_circ_0055486 | hsa_circ_0016641 |
| hsa_circ_0055489 | hsa_circ_0016642 |
| hsa_circ_0055490 | hsa_circ_0016643 |
| hsa_c | hsa_c |

| | |
|---|---|
| irc_0055491 | irc_0016657 |
| hsa_circ_0055492 | hsa_circ_0016656 |
| hsa_circ_0055525 | hsa_circ_0016670 |
| hsa_circ_0055532 | hsa_circ_0016671 |
| hsa_circ_0055533 | hsa_circ_0016672 |
| hsa_circ_0055534 | hsa_circ_0016673 |
| hsa_circ_0055550 | hsa_circ_0016674 |
| hsa_circ_0055626 | hsa_circ_0016675 |
| hsa_circ_0055633 | hsa_circ_0016676 |
| hsa_circ_0055634 | hsa_circ_0016757 |
| hsa_circ_0055635 | hsa_circ_0016771 |
| hsa_circ_0055636 | hsa_circ_0016831 |
| hsa_circ_0055638 | hsa_circ_0016833 |
| hsa_circ_0055639 | hsa_circ_0016832 |

| | |
|---|---|
| hsa_circ_0055640 | hsa_circ_0016915 |
| hsa_circ_0055641 | hsa_circ_0016916 |
| hsa_circ_0055642 | hsa_circ_0016917 |
| hsa_circ_0055643 | hsa_circ_0016922 |
| hsa_circ_0055801 | hsa_circ_0016926 |
| hsa_circ_0004147 | hsa_circ_0016935 |
| hsa_circ_0055813 | hsa_circ_0016936 |
| hsa_circ_0055817 | hsa_circ_0016949 |
| hsa_circ_0055823 | hsa_circ_0016963 |
| hsa_circ_0055824 | hsa_circ_0016972 |
| hsa_circ_0055826 | hsa_circ_0016977 |
| hsa_circ_0055827 | hsa_circ_0016982 |
| hsa_circ_0055829 | hsa_circ_0016983 |
| hsa_circ_00558 | hsa_circ_00039 |

| | |
|---|---|
| 30 | 29 |
| hsa_circ_0055832 | hsa_circ_0017140 |
| hsa_circ_0055833 | hsa_circ_0017141 |
| hsa_circ_0055835 | hsa_circ_0017142 |
| hsa_circ_0055837 | hsa_circ_0017143 |
| hsa_circ_001109 | hsa_circ_0017146 |
| hsa_circ_0055856 | hsa_circ_0017152 |
| hsa_circ_0055857 | hsa_circ_0017153 |
| hsa_circ_0055912 | hsa_circ_0017154 |
| hsa_circ_0007197 | hsa_circ_0017157 |
| hsa_circ_0003581 | hsa_circ_0017177 |
| hsa_circ_0007702 | hsa_circ_0002802 |
| hsa_circ_0002937 | hsa_circ_0052437 |
| hsa_circ_0003382 | hsa_circ_0052438 |
| hsa_c | hsa_c |

| | |
|---|---|
| irc_0004923 | irc_0052439 |
| hsa_circ_0004360 | hsa_circ_0052440 |
| hsa_circ_0007373 | hsa_circ_0052441 |
| hsa_circ_0003072 | hsa_circ_0052442 |
| hsa_circ_0004848 | hsa_circ_0052443 |
| hsa_circ_0007133 | hsa_circ_0052463 |
| hsa_circ_0008570 | hsa_circ_0052464 |
| hsa_circ_0056045 | hsa_circ_0052465 |
| hsa_circ_0056124 | hsa_circ_0052466 |
| hsa_circ_0056142 | hsa_circ_0052467 |
| hsa_circ_0008712 | hsa_circ_000689 |
| hsa_circ_0056190 | hsa_circ_0052584 |
| hsa_circ_0056191 | hsa_circ_0052586 |
| hsa_circ_00561 | hsa_circ_00525 |

| | |
|---|---|
| 94 | 87 |
| hsa_circ_0056195 | hsa_circ_0052588 |
| hsa_circ_0056196 | hsa_circ_0052882 |
| hsa_circ_0056198 | hsa_circ_0052883 |
| hsa_circ_0056199 | hsa_circ_0052884 |
| hsa_circ_0056203 | hsa_circ_0052885 |
| hsa_circ_0056204 | hsa_circ_0052890 |
| hsa_circ_0056206 | hsa_circ_0052891 |
| hsa_circ_0056208 | hsa_circ_0052886 |
| hsa_circ_0056209 | hsa_circ_0052887 |
| hsa_circ_0056292 | hsa_circ_0052888 |
| hsa_circ_0056291 | hsa_circ_0052889 |
| hsa_circ_0056293 | hsa_circ_0052933 |
| hsa_circ_0056294 | hsa_circ_0052934 |
| hsa_c | hsa_c |

| | |
|---|---|
| irc_0056295 | irc_0052935 |
| hsa_circ_0056364 | hsa_circ_0052936 |
| hsa_circ_0056476 | hsa_circ_0052937 |
| hsa_circ_0056477 | hsa_circ_0052938 |
| hsa_circ_0056478 | hsa_circ_0052939 |
| hsa_circ_0056479 | hsa_circ_0052940 |
| hsa_circ_0056480 | hsa_circ_0052941 |
| hsa_circ_0056509 | hsa_circ_0052942 |
| hsa_circ_0056510 | hsa_circ_0052974 |
| hsa_circ_0056525 | hsa_circ_0052975 |
| hsa_circ_0056527 | hsa_circ_0052979 |
| hsa_circ_0056529 | hsa_circ_0052980 |
| hsa_circ_0056530 | hsa_circ_0052981 |
| hsa_circ_0056531 | hsa_circ_0052982 |

| | |
|---|---|
| hsa_circ_0056532 | hsa_circ_0053004 |
| hsa_circ_0056533 | hsa_circ_0053007 |
| hsa_circ_0056534 | hsa_circ_0053009 |
| hsa_circ_0008036 | hsa_circ_0053034 |
| hsa_circ_0056545 | hsa_circ_0053035 |
| hsa_circ_0056549 | hsa_circ_0053036 |
| hsa_circ_0056551 | hsa_circ_0053037 |
| hsa_circ_0056552 | hsa_circ_0053038 |
| hsa_circ_0056566 | hsa_circ_0053093 |
| hsa_circ_0056583 | hsa_circ_0053137 |
| hsa_circ_0056584 | hsa_circ_0053144 |
| hsa_circ_0056587 | hsa_circ_0053145 |
| hsa_circ_0056615 | hsa_circ_0053146 |
| hsa_circ_00566 | hsa_circ_00532 |

| | |
|---|---|
| 65 | 61 |
| hsa_circ_0056730 | hsa_circ_0053263 |
| hsa_circ_0056739 | hsa_circ_0053280 |
| hsa_circ_0056742 | hsa_circ_0053283 |
| hsa_circ_0056769 | hsa_circ_0004337 |
| hsa_circ_0056802 | hsa_circ_0053375 |
| hsa_circ_0056900 | hsa_circ_0053376 |
| hsa_circ_0056901 | hsa_circ_002075 |
| hsa_circ_0056925 | hsa_circ_0053434 |
| hsa_circ_0007529 | hsa_circ_0053435 |
| hsa_circ_0057058 | hsa_circ_0053436 |
| hsa_circ_0057065 | hsa_circ_0053437 |
| hsa_circ_0057066 | hsa_circ_0053438 |
| hsa_circ_0057067 | hsa_circ_0053444 |
| hsa_c | hsa_c |

| | |
|---|---|
| irc_0057124 | irc_0053445 |
| hsa_circ_0057169 | hsa_circ_0053446 |
| hsa_circ_0057213 | hsa_circ_0053439 |
| hsa_circ_0057258 | hsa_circ_0053440 |
| hsa_circ_0057259 | hsa_circ_0053441 |
| hsa_circ_0057260 | hsa_circ_0053442 |
| hsa_circ_0057261 | hsa_circ_0053443 |
| hsa_circ_0057262 | hsa_circ_001287 |
| hsa_circ_0057263 | hsa_circ_0004182 |
| hsa_circ_0057334 | hsa_circ_0004336 |
| hsa_circ_0057335 | hsa_circ_0007972 |
| hsa_circ_0057497 | hsa_circ_0005507 |
| hsa_circ_0057507 | hsa_circ_0006294 |
| hsa_circ_0057508 | hsa_circ_0006330 |

| | | | |
|---|---|---|---|
| | hsa_circ_0057509 | hsa_circ_0007971 | |
| | hsa_circ_0057511 | hsa_circ_0054025 | |
| | hsa_circ_0057512 | hsa_circ_0006444 | |
| | hsa_circ_0057513 | hsa_circ_0054167 | |
| | hsa_circ_0057514 | hsa_circ_0054181 | |
| | hsa_circ_0057515 | hsa_circ_0054182 | |
| | hsa_circ_0057539 | hsa_circ_0054436 | |
| | hsa_circ_0057548 | hsa_circ_0054473 | |
| | hsa_circ_0057552 | hsa_circ_0054475 | |
| | hsa_circ_0057553 | hsa_circ_0054477 | |
| | hsa_circ_0057554 | hsa_circ_0054478 | |
| | hsa_circ_0057555 | hsa_circ_0054537 | |
| | hsa_circ_0057556 | hsa_circ_0054548 | |
| | hsa_circ_00575 | hsa_circ_00545 | |

| | |
|---|---|
| 57 | 53 |
| hsa_circ_0057558 | hsa_circ_0054554 |
| hsa_circ_0057559 | hsa_circ_0003165 |
| hsa_circ_0057658 | hsa_circ_0054669 |
| hsa_circ_0057659 | hsa_circ_0054671 |
| hsa_circ_0057660 | hsa_circ_0054674 |
| hsa_circ_0057661 | hsa_circ_0054675 |
| hsa_circ_0057662 | hsa_circ_0054676 |
| hsa_circ_0057663 | hsa_circ_0054677 |
| hsa_circ_0057664 | hsa_circ_001766 |
| hsa_circ_0057665 | hsa_circ_0008534 |
| hsa_circ_0057666 | hsa_circ_0054699 |
| hsa_circ_0057694 | hsa_circ_0054700 |
| hsa_circ_0057723 | hsa_circ_000684 |
| hsa_c | hsa_c |

| | |
|---|---|
| irc_0057754 | irc_0002370 |
| hsa_circ_0057755 | hsa_circ_0006759 |
| hsa_circ_0057756 | hsa_circ_0054924 |
| hsa_circ_0057897 | hsa_circ_0054944 |
| hsa_circ_0057898 | hsa_circ_0054951 |
| hsa_circ_0057899 | hsa_circ_0054955 |
| hsa_circ_0057900 | hsa_circ_0054956 |
| hsa_circ_0057955 | hsa_circ_0054957 |
| hsa_circ_0057964 | hsa_circ_0054958 |
| hsa_circ_0057965 | hsa_circ_0054959 |
| hsa_circ_0057966 | hsa_circ_0054960 |
| hsa_circ_00023 | hsa_circ_0054964 |
| hsa_circ_0057978 | hsa_circ_0054965 |
| hsa_circ_0057979 | hsa_circ_0055069 |

| | |
|---|---|
| hsa_circ_0057981 | hsa_circ_0055070 |
| hsa_circ_0057982 | hsa_circ_0055071 |
| hsa_circ_0057984 | hsa_circ_0055072 |
| hsa_circ_0058088 | hsa_circ_0055101 |
| hsa_circ_0058089 | hsa_circ_0055114 |
| hsa_circ_0058090 | hsa_circ_0055136 |
| hsa_circ_0058196 | hsa_circ_0055137 |
| hsa_circ_0058197 | hsa_circ_0055138 |
| hsa_circ_0058219 | hsa_circ_0055139 |
| hsa_circ_0058220 | hsa_circ_0055140 |
| hsa_circ_0058398 | hsa_circ_0055141 |
| hsa_circ_0058399 | hsa_circ_0055207 |
| hsa_circ_0058400 | hsa_circ_0055230 |
| hsa_circ_0 | hsa_circ_0 |

| | |
|---|---|
| 058401 | 055231 |
| hsa_circ_0004821 | hsa_circ_0055234 |
| hsa_circ_0058443 | hsa_circ_0055236 |
| hsa_circ_0058444 | hsa_circ_0055237 |
| hsa_circ_0058445 | hsa_circ_0055238 |
| hsa_circ_0058446 | hsa_circ_0055240 |
| hsa_circ_0058447 | hsa_circ_0011126 |
| hsa_circ_0058448 | hsa_circ_0055249 |
| hsa_circ_0058449 | hsa_circ_0055250 |
| hsa_circ_0058450 | hsa_circ_0055255 |
| hsa_circ_0058457 | hsa_circ_0055256 |
| hsa_circ_0004153 | hsa_circ_0055257 |
| hsa_circ_0021162 | hsa_circ_0055258 |
| hsa_circ_0011118 | hsa_circ_0055259 |

| | |
|---|---|
| hsa_circ_0058665 | hsa_circ_0055260 |
| hsa_circ_0058666 | hsa_circ_0055261 |
| hsa_circ_0058667 | hsa_circ_0055264 |
| hsa_circ_0058713 | hsa_circ_0055265 |
| hsa_circ_0058722 | hsa_circ_0003243 |
| hsa_circ_0058761 | hsa_circ_0055268 |
| hsa_circ_0058778 | hsa_circ_0055270 |
| hsa_circ_0058779 | hsa_circ_0055271 |
| hsa_circ_0058780 | hsa_circ_0055272 |
| hsa_circ_0058815 | hsa_circ_0055289 |
| hsa_circ_0059063 | hsa_circ_0055291 |
| hsa_circ_0059065 | hsa_circ_0055335 |
| hsa_circ_0059066 | hsa_circ_0055332 |
| hsa_circ_00591 | hsa_circ_00044 |

| | |
|---|---|
| 07 | 20 |
| hsa_circ_0064024 | hsa_circ_0055422 |
| hsa_circ_0064025 | hsa_circ_0055423 |
| hsa_circ_0064026 | hsa_circ_0055421 |
| hsa_circ_0002453 | hsa_circ_0055438 |
| hsa_circ_0064098 | hsa_circ_0055550 |
| hsa_circ_0064100 | hsa_circ_0055625 |
| hsa_circ_0064101 | hsa_circ_0055633 |
| hsa_circ_0015177 | hsa_circ_0055634 |
| hsa_circ_0005867 | hsa_circ_0055635 |
| hsa_circ_0064193 | hsa_circ_0055636 |
| hsa_circ_0064196 | hsa_circ_0055637 |
| hsa_circ_0064197 | hsa_circ_0055638 |
| hsa_circ_0064198 | hsa_circ_0055639 |
| hsa_c | hsa_c |

| | |
|---|---|
| irc_0064201 | irc_0055643 |
| hsa_circ_0064210 | hsa_circ_0055640 |
| hsa_circ_0064212 | hsa_circ_0055641 |
| hsa_circ_0064217 | hsa_circ_0055642 |
| hsa_circ_0064225 | hsa_circ_0055693 |
| hsa_circ_0064227 | hsa_circ_0055761 |
| hsa_circ_0064229 | hsa_circ_0055821 |
| hsa_circ_0064235 | hsa_circ_0055893 |
| hsa_circ_0064236 | hsa_circ_0055894 |
| hsa_circ_0064249 | hsa_circ_0055895 |
| hsa_circ_00692 | hsa_circ_0055896 |
| hsa_circ_0064271 | hsa_circ_0055897 |
| hsa_circ_0064272 | hsa_circ_0055898 |
| hsa_circ_0064335 | hsa_circ_0055899 |

| | |
|---|---|
| hsa_circ_0064336 | hsa_circ_0055912 |
| hsa_circ_0064337 | hsa_circ_0055929 |
| hsa_circ_0064338 | hsa_circ_0007197 |
| hsa_circ_0064339 | hsa_circ_0003581 |
| hsa_circ_0064357 | hsa_circ_0007724 |
| hsa_circ_0064391 | hsa_circ_0008364 |
| hsa_circ_0064392 | hsa_circ_0009078 |
| hsa_circ_0064394 | hsa_circ_0055987 |
| hsa_circ_0064395 | hsa_circ_0002728 |
| hsa_circ_0064396 | hsa_circ_0002937 |
| hsa_circ_0064397 | hsa_circ_0004923 |
| hsa_circ_0064398 | hsa_circ_0003382 |
| hsa_circ_0064399 | hsa_circ_0007774 |
| hsa_circ_00644 | hsa_circ_00560 |

| | |
|---|---|
| 00 | 06 |
| hsa_circ_006448 7 | hsa_circ_000737 3 |
| hsa_circ_006448 8 | hsa_circ_000436 0 |
| hsa_circ_006448 9 | hsa_circ_000307 2 |
| hsa_circ_006449 8 | hsa_circ_000713 3 |
| hsa_circ_006454 1 | hsa_circ_000857 0 |
| hsa_circ_006454 2 | hsa_circ_000484 8 |
| hsa_circ_006458 9 | hsa_circ_005604 4 |
| hsa_circ_006459 0 | hsa_circ_005613 5 |
| hsa_circ_006459 1 | hsa_circ_005613 6 |
| hsa_circ_006459 2 | hsa_circ_005613 8 |
| hsa_circ_006460 0 | hsa_circ_005613 9 |
| hsa_circ_006460 1 | hsa_circ_005614 0 |
| hsa_circ_006462 7 | hsa_circ_000401 2 |
| hsa_c | hsa_c |

| | |
|---|---|
| irc_0064659 | irc_0056144 |
| hsa_circ_0064660 | hsa_circ_0056145 |
| hsa_circ_0064661 | hsa_circ_0056142 |
| hsa_circ_0064662 | hsa_circ_0056153 |
| hsa_circ_0064673 | hsa_circ_0056155 |
| hsa_circ_0064703 | hsa_circ_0056151 |
| hsa_circ_0064704 | hsa_circ_0056222 |
| hsa_circ_0064705 | hsa_circ_0056223 |
| hsa_circ_0064707 | hsa_circ_0056224 |
| hsa_circ_0064708 | hsa_circ_0056221 |
| hsa_circ_0064709 | hsa_circ_0056232 |
| hsa_circ_0064711 | hsa_circ_0056234 |
| hsa_circ_0064712 | hsa_circ_0056242 |
| hsa_circ_0064713 | hsa_circ_0056236 |

| | |
|---|---|
| hsa_circ_0064714 | hsa_circ_0056237 |
| hsa_circ_0064722 | hsa_circ_0056238 |
| hsa_circ_0064817 | hsa_circ_0056239 |
| hsa_circ_0064893 | hsa_circ_0056240 |
| hsa_circ_0064895 | hsa_circ_0056241 |
| hsa_circ_0064896 | hsa_circ_0007517 |
| hsa_circ_0064899 | hsa_circ_0056286 |
| hsa_circ_0065012 | hsa_circ_0056288 |
| hsa_circ_0065063 | hsa_circ_0056303 |
| hsa_circ_0065064 | hsa_circ_0056363 |
| hsa_circ_0065065 | hsa_circ_0056441 |
| hsa_circ_0065104 | hsa_circ_0056457 |
| hsa_circ_0065105 | hsa_circ_0056458 |
| hsa_circ_00651 | hsa_circ_00564 |

| | |
|---|---|
| 06 | 59 |
| hsa_circ_0065187 | hsa_circ_0056456 |
| hsa_circ_0065240 | hsa_circ_0056510 |
| hsa_circ_0065385 | hsa_circ_0056509 |
| hsa_circ_0065386 | hsa_circ_0056622 |
| hsa_circ_0065479 | hsa_circ_0056623 |
| hsa_circ_0065481 | hsa_circ_0056624 |
| hsa_circ_0065482 | hsa_circ_0056625 |
| hsa_circ_0065483 | hsa_circ_0056626 |
| hsa_circ_0065484 | hsa_circ_0056627 |
| hsa_circ_0065485 | hsa_circ_0056628 |
| hsa_circ_0065486 | hsa_circ_0056629 |
| hsa_circ_0065499 | hsa_circ_0056632 |
| hsa_circ_0065525 | hsa_circ_0056630 |
| hsa_c | hsa_c |

| | |
|---|---|
| irc_0065526 | irc_0056631 |
| hsa_circ_0065527 | hsa_circ_0056690 |
| hsa_circ_0065554 | hsa_circ_0056727 |
| hsa_circ_0065555 | hsa_circ_0056769 |
| hsa_circ_0002561 | hsa_circ_0056862 |
| hsa_circ_0065824 | hsa_circ_0056863 |
| hsa_circ_0065827 | hsa_circ_0056864 |
| hsa_circ_0065830 | hsa_circ_0007529 |
| hsa_circ_0065831 | hsa_circ_0007941 |
| hsa_circ_0065832 | hsa_circ_0057139 |
| hsa_circ_0065852 | hsa_circ_0057140 |
| hsa_circ_0066037 | hsa_circ_0057170 |
| hsa_circ_0066038 | hsa_circ_0004328 |
| hsa_circ_0066039 | hsa_circ_0057308 |

| | |
|---|---|
| hsa_circ_0066040 | hsa_circ_0057345 |
| hsa_circ_0066221 | hsa_circ_0057416 |
| hsa_circ_0066222 | hsa_circ_0057417 |
| hsa_circ_0066223 | hsa_circ_0057421 |
| hsa_circ_0066224 | hsa_circ_0057539 |
| hsa_circ_001831 | hsa_circ_0057720 |
| hsa_circ_0066287 | hsa_circ_0057722 |
| hsa_circ_0066288 | hsa_circ_0057771 |
| hsa_circ_0066292 | hsa_circ_0057836 |
| hsa_circ_0066293 | hsa_circ_0057837 |
| hsa_circ_0066294 | hsa_circ_0057843 |
| hsa_circ_0066295 | hsa_circ_0057849 |
| hsa_circ_0066296 | hsa_circ_0057854 |
| hsa_circ_00662 | hsa_circ_00578 |

| | |
|---|---|
| 97 | 55 |
| hsa_circ_0066298 | hsa_circ_0057859 |
| hsa_circ_0066299 | hsa_circ_0057860 |
| hsa_circ_0066300 | hsa_circ_0057861 |
| hsa_circ_0066301 | hsa_circ_0058004 |
| hsa_circ_0066302 | hsa_circ_0058018 |
| hsa_circ_0066304 | hsa_circ_0058020 |
| hsa_circ_0066306 | hsa_circ_0058022 |
| hsa_circ_0066307 | hsa_circ_0058023 |
| hsa_circ_0066308 | hsa_circ_0058024 |
| hsa_circ_0066309 | hsa_circ_0058160 |
| hsa_circ_0066311 | hsa_circ_0058190 |
| hsa_circ_0066312 | hsa_circ_0058229 |
| hsa_circ_0066313 | hsa_circ_0058230 |
| hsa_c | hsa_c |

| | |
|---|---|
| irc_006631 4 | irc_005824 2 |
| hsa_circ_0066315 | hsa_circ_0058243 |
| hsa_circ_0066316 | hsa_circ_0058282 |
| hsa_circ_0066317 | hsa_circ_0058284 |
| hsa_circ_0066318 | hsa_circ_0058285 |
| hsa_circ_0066425 | hsa_circ_0058286 |
| hsa_circ_0066420 | hsa_circ_0058311 |
| hsa_circ_0066432 | hsa_circ_0058314 |
| hsa_circ_0066436 | hsa_circ_0058316 |
| hsa_circ_0066504 | hsa_circ_0058317 |
| hsa_circ_0066506 | hsa_circ_0058356 |
| hsa_circ_0066507 | hsa_circ_0058357 |
| hsa_circ_0066508 | hsa_circ_0058358 |
| hsa_circ_0066606 | hsa_circ_0058359 |

| | |
|---|---|
| hsa_circ_0066610 | hsa_circ_0058361 |
| hsa_circ_0066611 | hsa_circ_0058366 |
| hsa_circ_0066635 | hsa_circ_0058368 |
| hsa_circ_0007601 | hsa_circ_0058370 |
| hsa_circ_0066636 | hsa_circ_0058371 |
| hsa_circ_0066637 | hsa_circ_0058373 |
| hsa_circ_0066638 | hsa_circ_0058381 |
| hsa_circ_0066639 | hsa_circ_0003591 |
| hsa_circ_0066640 | hsa_circ_0058487 |
| hsa_circ_0066687 | hsa_circ_0058491 |
| hsa_circ_0066690 | hsa_circ_0058498 |
| hsa_circ_0066691 | hsa_circ_0002054 |
| hsa_circ_0066712 | hsa_circ_001283 |
| hsa_circ_00667 | hsa_circ_00586 |

| | |
|---|---|
| 73 | 65 |
| hsa_circ_0066774 | hsa_circ_0058696 |
| hsa_circ_0066833 | hsa_circ_0058709 |
| hsa_circ_0066836 | hsa_circ_0058710 |
| hsa_circ_0066835 | hsa_circ_0058711 |
| hsa_circ_0066972 | hsa_circ_0058712 |
| hsa_circ_0066976 | hsa_circ_0058713 |
| hsa_circ_0067004 | hsa_circ_0058714 |
| hsa_circ_0067038 | hsa_circ_0058715 |
| hsa_circ_0067039 | hsa_circ_0058716 |
| hsa_circ_0067040 | hsa_circ_0006121 |
| hsa_circ_0067041 | hsa_circ_0058885 |
| hsa_circ_0067089 | hsa_circ_0058883 |
| hsa_circ_0067092 | hsa_circ_0058908 |
| hsa_c | hsa_c |

| | |
|---|---|
| irc_0006245 | irc_0058951 |
| hsa_circ_0067117 | hsa_circ_0058979 |
| hsa_circ_0067118 | hsa_circ_0058976 |
| hsa_circ_0067119 | hsa_circ_0059004 |
| hsa_circ_0006346 | hsa_circ_0064024 |
| hsa_circ_0008923 | hsa_circ_0064025 |
| hsa_circ_0067232 | hsa_circ_0064026 |
| hsa_circ_0067234 | hsa_circ_0064094 |
| hsa_circ_0067236 | hsa_circ_0064095 |
| hsa_circ_0067237 | hsa_circ_0064096 |
| hsa_circ_0067239 | hsa_circ_0064097 |
| hsa_circ_0067240 | hsa_circ_0064098 |
| hsa_circ_0011355 | hsa_circ_0064150 |
| hsa_circ_0067318 | hsa_circ_0064153 |

| | |
|---|---|
| hsa_circ_0067319 | hsa_circ_0064146 |
| hsa_circ_0067374 | hsa_circ_0064160 |
| hsa_circ_0067394 | hsa_circ_0064164 |
| hsa_circ_0067395 | hsa_circ_0064162 |
| hsa_circ_0067398 | hsa_circ_0064159 |
| hsa_circ_0067399 | hsa_circ_0064184 |
| hsa_circ_0067400 | hsa_circ_0064183 |
| hsa_circ_0067440 | hsa_circ_0064198 |
| hsa_circ_0067441 | hsa_circ_0002865 |
| hsa_circ_0067442 | hsa_circ_0064329 |
| hsa_circ_0067450 | hsa_circ_0007278 |
| hsa_circ_0067485 | hsa_circ_0064350 |
| hsa_circ_0067544 | hsa_circ_0064498 |
| hsa_circ_0675 | hsa_circ_0645 |

| | |
|---|---|
| 45 | 28 |
| hsa_circ_0067546 | hsa_circ_0064529 |
| hsa_circ_0067547 | hsa_circ_0064530 |
| hsa_circ_0067548 | hsa_circ_0064531 |
| hsa_circ_0067549 | hsa_circ_0064615 |
| hsa_circ_0067550 | hsa_circ_0064616 |
| hsa_circ_0067551 | hsa_circ_0064618 |
| hsa_circ_0067552 | hsa_circ_0064619 |
| hsa_circ_0067553 | hsa_circ_0064620 |
| hsa_circ_0067554 | hsa_circ_0064621 |
| hsa_circ_0067565 | hsa_circ_0064622 |
| hsa_circ_0067570 | hsa_circ_0002901 |
| hsa_circ_0067571 | hsa_circ_0064673 |
| hsa_circ_0067666 | hsa_circ_0064684 |
| hsa_c | hsa_c |

| | |
|---|---|
| irc_0067681 | irc_0064817 |
| hsa_circ_0067699 | hsa_circ_0064875 |
| hsa_circ_0003502 | hsa_circ_0064874 |
| hsa_circ_0004565 | hsa_circ_0064895 |
| hsa_circ_0005342 | hsa_circ_0064896 |
| hsa_circ_0006801 | hsa_circ_0064893 |
| hsa_circ_0067718 | hsa_circ_0064927 |
| hsa_circ_0067720 | hsa_circ_0064973 |
| hsa_circ_0067721 | hsa_circ_0064970 |
| hsa_circ_0067722 | hsa_circ_0065025 |
| hsa_circ_0067723 | hsa_circ_0065024 |
| hsa_circ_0067724 | hsa_circ_0065023 |
| hsa_circ_0067725 | hsa_circ_0065063 |
| hsa_circ_0067726 | hsa_circ_0065064 |

| | |
|---|---|
| hsa_circ_0067727 | hsa_circ_0065065 |
| hsa_circ_0018891 | hsa_circ_0065133 |
| hsa_circ_0067863 | hsa_circ_0005435 |
| hsa_circ_0067884 | hsa_circ_0065240 |
| hsa_circ_0020000 | hsa_circ_0065246 |
| hsa_circ_0019222 | hsa_circ_0065247 |
| hsa_circ_0067949 | hsa_circ_0065254 |
| hsa_circ_0067979 | hsa_circ_0065268 |
| hsa_circ_0067988 | hsa_circ_0065279 |
| hsa_circ_0068009 | hsa_circ_0065282 |
| hsa_circ_0068038 | hsa_circ_0065273 |
| hsa_circ_0068039 | hsa_circ_0005367 |
| hsa_circ_0068040 | hsa_circ_0012996 |
| hsa_circ_00680 | hsa_circ_00653 |

| | |
|---|---|
| 41 | 49 |
| hsa_circ_0068042 | hsa_circ_0065348 |
| hsa_circ_0068043 | hsa_circ_0003091 |
| hsa_circ_0068045 | hsa_circ_0065391 |
| hsa_circ_0068047 | hsa_circ_0065390 |
| hsa_circ_0068048 | hsa_circ_0065392 |
| hsa_circ_0068049 | hsa_circ_0065482 |
| hsa_circ_0068050 | hsa_circ_0065483 |
| hsa_circ_0068051 | hsa_circ_0065484 |
| hsa_circ_0068052 | hsa_circ_0065479 |
| hsa_circ_0068053 | hsa_circ_0065486 |
| hsa_circ_0068068 | hsa_circ_0065503 |
| hsa_circ_0068074 | hsa_circ_0065510 |
| hsa_circ_0068145 | hsa_circ_0065511 |
| hsa_c | hsa_c |

| | |
|---|---|
| irc_0068188 | irc_0065515 |
| hsa_circ_0068237 | hsa_circ_0065509 |
| hsa_circ_0068386 | hsa_circ_0065551 |
| hsa_circ_0068387 | hsa_circ_0065552 |
| hsa_circ_0068393 | hsa_circ_0065557 |
| hsa_circ_0068394 | hsa_circ_0065558 |
| hsa_circ_0068395 | hsa_circ_0065559 |
| hsa_circ_0068418 | hsa_circ_0065560 |
| hsa_circ_0068487 | hsa_circ_0065561 |
| hsa_circ_0068490 | hsa_circ_0065562 |
| hsa_circ_0068498 | hsa_circ_0065614 |
| hsa_circ_0068499 | hsa_circ_0065615 |
| hsa_circ_0068500 | hsa_circ_0065616 |
| hsa_circ_0068512 | hsa_circ_0065620 |

| | |
|---|---|
| hsa_circ_0007347 | hsa_circ_0065621 |
| hsa_circ_0068569 | hsa_circ_0065627 |
| hsa_circ_0068570 | hsa_circ_0065628 |
| hsa_circ_0068572 | hsa_circ_0065629 |
| hsa_circ_0068573 | hsa_circ_0065630 |
| hsa_circ_0068575 | hsa_circ_0065632 |
| hsa_circ_0068576 | hsa_circ_0004581 |
| hsa_circ_0068781 | hsa_circ_0065659 |
| hsa_circ_0068858 | hsa_circ_0065660 |
| hsa_circ_0068860 | hsa_circ_0065670 |
| hsa_circ_0068863 | hsa_circ_0065662 |
| hsa_circ_0068864 | hsa_circ_0065663 |
| hsa_circ_0068866 | hsa_circ_0065664 |
| hsa_circ_0688 | hsa_circ_0656 |

| | |
|---|---|
| 67 | 65 |
| hsa_circ_006887 8 | hsa_circ_006566 6 |
| hsa_circ_006887 9 | hsa_circ_006567 1 |
| hsa_circ_006888 0 | hsa_circ_006567 2 |
| hsa_circ_006888 1 | hsa_circ_006572 3 |
| hsa_circ_006888 2 | hsa_circ_006572 6 |
| hsa_circ_006889 3 | hsa_circ_006572 7 |
| hsa_circ_006912 4 | hsa_circ_006573 1 |
| hsa_circ_006912 5 | hsa_circ_006573 6 |
| hsa_circ_006919 4 | hsa_circ_006573 7 |
| hsa_circ_006919 5 | hsa_circ_006573 8 |
| hsa_circ_006919 6 | hsa_circ_006571 5 |
| hsa_circ_006919 7 | hsa_circ_006574 1 |
| hsa_circ_006922 2 | hsa_circ_006574 2 |

| | |
|---|---|
| hsa_circ_0069223 | hsa_circ_0065740 |
| hsa_circ_0069283 | hsa_circ_0065746 |
| hsa_circ_0069317 | hsa_circ_0065747 |
| hsa_circ_0007107 | hsa_circ_0065749 |
| hsa_circ_0069364 | hsa_circ_0065750 |
| hsa_circ_0069369 | hsa_circ_0065751 |
| hsa_circ_0069372 | hsa_circ_0065752 |
| hsa_circ_0069373 | hsa_circ_0065753 |
| hsa_circ_0069374 | hsa_circ_0065754 |
| hsa_circ_0069375 | hsa_circ_0065827 |
| hsa_circ_0069376 | hsa_circ_0065831 |
| hsa_circ_0069393 | hsa_circ_0065838 |
| hsa_circ_0069441 | hsa_circ_0065853 |
| hsa_circ_00694 | hsa_circ_00658 |

| | |
|---|---|
| 43 | 65 |
| hsa_circ_0069444 | hsa_circ_0065866 |
| hsa_circ_0069445 | hsa_circ_0005449 |
| hsa_circ_0069446 | hsa_circ_0065922 |
| hsa_circ_0069447 | hsa_circ_0065924 |
| hsa_circ_0069448 | hsa_circ_0065925 |
| hsa_circ_0069449 | hsa_circ_0005416 |
| hsa_circ_0069475 | hsa_circ_0065955 |
| hsa_circ_0069476 | hsa_circ_0065956 |
| hsa_circ_0069478 | hsa_circ_0065954 |
| hsa_circ_0069549 | hsa_circ_0065962 |
| hsa_circ_0069616 | hsa_circ_0065975 |
| hsa_circ_0069620 | hsa_circ_0066036 |
| hsa_circ_0069621 | hsa_circ_0066037 |
| hsa_c | hsa_c |

| | |
|---|---|
| irc_0002021 | irc_0066038 |
| hsa_circ_0069686 | hsa_circ_0066039 |
| hsa_circ_0069698 | hsa_circ_0066071 |
| hsa_circ_0069699 | hsa_circ_0066072 |
| hsa_circ_0069705 | hsa_circ_0066073 |
| hsa_circ_0069706 | hsa_circ_0066074 |
| hsa_circ_0069707 | hsa_circ_0002801 |
| hsa_circ_0069708 | hsa_circ_0066082 |
| hsa_circ_0069709 | hsa_circ_0066091 |
| hsa_circ_0069710 | hsa_circ_0066094 |
| hsa_circ_001301 | hsa_circ_0066095 |
| hsa_circ_0069852 | hsa_circ_0066123 |
| hsa_circ_0069867 | hsa_circ_0066124 |
| hsa_circ_0069920 | hsa_circ_0066128 |

| | |
|---|---|
| hsa_circ_0069932 | hsa_circ_0066129 |
| hsa_circ_0069933 | hsa_circ_0066127 |
| hsa_circ_0069934 | hsa_circ_0066130 |
| hsa_circ_0069935 | hsa_circ_0066131 |
| hsa_circ_0070014 | hsa_circ_0066139 |
| hsa_circ_0070047 | hsa_circ_0066138 |
| hsa_circ_0070050 | hsa_circ_0066141 |
| hsa_circ_0070049 | hsa_circ_0066142 |
| hsa_circ_0070094 | hsa_circ_0066145 |
| hsa_circ_0070126 | hsa_circ_0066147 |
| hsa_circ_0070162 | hsa_circ_0066148 |
| hsa_circ_0070177 | hsa_circ_0066149 |
| hsa_circ_0070182 | hsa_circ_0066389 |
| hsa_circ_00090 | hsa_circ_00664 |

| | |
|---|---|
| 36 | 20 |
| hsa_circ_0070373 | hsa_circ_0066424 |
| hsa_circ_0000009 | hsa_circ_0066419 |
| hsa_circ_0070393 | hsa_circ_0066451 |
| hsa_circ_0070416 | hsa_circ_0066454 |
| hsa_circ_0070424 | hsa_circ_0066456 |
| hsa_circ_0070426 | hsa_circ_0066457 |
| hsa_circ_0070429 | hsa_circ_0066458 |
| hsa_circ_0005233 | hsa_circ_0066468 |
| hsa_circ_0070466 | hsa_circ_0003210 |
| hsa_circ_0070468 | hsa_circ_0003806 |
| hsa_circ_0070472 | hsa_circ_0005452 |
| hsa_circ_0070473 | hsa_circ_0066593 |
| hsa_circ_0070474 | hsa_circ_0066595 |
| hsa_c | hsa_c |

| | |
|---|---|
| irc_0 | irc_0 |
| 0130 | 0666 |
| 5 | 06 |
| hsa_c | hsa_c |
| irc_0 | irc_0 |
| 0704 | 0666 |
| 90 | 35 |
| hsa_c | hsa_c |
| irc_0 | irc_0 |
| 0705 | 0049 |
| 79 | 33 |
| hsa_c | hsa_c |
| irc_0 | irc_0 |
| 0705 | 0667 |
| 80 | 03 |
| hsa_c | hsa_c |
| irc_0 | irc_0 |
| 0705 | 0668 |
| 81 | 27 |
| hsa_c | hsa_c |
| irc_0 | irc_0 |
| 0705 | 0668 |
| 82 | 28 |
| hsa_c | hsa_c |
| irc_0 | irc_0 |
| 0705 | 0668 |
| 83 | 29 |
| hsa_c | hsa_c |
| irc_0 | irc_0 |
| 0708 | 0668 |
| 44 | 32 |
| hsa_c | hsa_c |
| irc_0 | irc_0 |
| 0708 | 0668 |
| 52 | 33 |
| hsa_c | hsa_c |
| irc_0 | irc_0 |
| 0708 | 0668 |
| 53 | 35 |
| hsa_c | hsa_c |
| irc_0 | irc_0 |
| 0708 | 0668 |
| 54 | 38 |
| hsa_c | hsa_c |
| irc_0 | irc_0 |
| 0708 | 0668 |
| 56 | 39 |
| hsa_c | hsa_c |
| irc_0 | irc_0 |
| 0708 | 0668 |
| 59 | 37 |
| hsa_c | hsa_c |
| irc_0 | irc_0 |
| 0708 | 0668 |
| 62 | 79 |

| | |
|---|---|
| hsa_circ_0070863 | hsa_circ_0066913 |
| hsa_circ_0070864 | hsa_circ_0066910 |
| hsa_circ_0070865 | hsa_circ_0006560 |
| hsa_circ_0070887 | hsa_circ_0067084 |
| hsa_circ_0070896 | hsa_circ_0067089 |
| hsa_circ_0070897 | hsa_circ_0067092 |
| hsa_circ_0070898 | hsa_circ_0067128 |
| hsa_circ_0070920 | hsa_circ_0067138 |
| hsa_circ_0070947 | hsa_circ_0067181 |
| hsa_circ_0070948 | hsa_circ_0067194 |
| hsa_circ_0070949 | hsa_circ_0067220 |
| hsa_circ_0070952 | hsa_circ_0067242 |
| hsa_circ_0070969 | hsa_circ_0067244 |
| hsa_circ_00709 | hsa_circ_00672 |

| | |
|---|---|
| 70 | 47 |
| hsa_circ_0071004 | hsa_circ_0067248 |
| hsa_circ_0071005 | hsa_circ_0067292 |
| hsa_circ_0071006 | hsa_circ_0067303 |
| hsa_circ_0071007 | hsa_circ_0067299 |
| hsa_circ_0071008 | hsa_circ_0067305 |
| hsa_circ_0071009 | hsa_circ_0067306 |
| hsa_circ_0071040 | hsa_circ_0067307 |
| hsa_circ_0071041 | hsa_circ_0067308 |
| hsa_circ_0071079 | hsa_circ_0067298 |
| hsa_circ_0071171 | hsa_circ_0067391 |
| hsa_circ_0071182 | hsa_circ_0067443 |
| hsa_circ_0071183 | hsa_circ_0067598 |
| hsa_circ_0071184 | hsa_circ_0067599 |
| hsa_c | hsa_c |

| | |
|---|---|
| irc_0071202 | irc_0067600 |
| hsa_circ_0071203 | hsa_circ_0067601 |
| hsa_circ_0071221 | hsa_circ_0067602 |
| hsa_circ_0071222 | hsa_circ_0067603 |
| hsa_circ_0071228 | hsa_circ_0067604 |
| hsa_circ_0071229 | hsa_circ_0067597 |
| hsa_circ_0071280 | hsa_circ_0067629 |
| hsa_circ_0071282 | hsa_circ_0067630 |
| hsa_circ_0071283 | hsa_circ_0067633 |
| hsa_circ_0071284 | hsa_circ_0067639 |
| hsa_circ_0071319 | hsa_circ_0067640 |
| hsa_circ_0071320 | hsa_circ_0067642 |
| hsa_circ_0071334 | hsa_circ_0067643 |
| hsa_circ_0071335 | hsa_circ_0067644 |

| | |
|---|---|
| hsa_circ_0071336 | hsa_circ_0067625 |
| hsa_circ_0071341 | hsa_circ_0006731 |
| hsa_circ_0071337 | hsa_circ_0067680 |
| hsa_circ_0071338 | hsa_circ_0003756 |
| hsa_circ_0071339 | hsa_circ_0067681 |
| hsa_circ_0071340 | hsa_circ_0067689 |
| hsa_circ_0071436 | hsa_circ_0067844 |
| hsa_circ_0071437 | hsa_circ_0067884 |
| hsa_circ_0002212 | hsa_circ_0067972 |
| hsa_circ_0004181 | hsa_circ_0067966 |
| hsa_circ_0005274 | hsa_circ_0068042 |
| hsa_circ_0006680 | hsa_circ_0068063 |
| hsa_circ_0071493 | hsa_circ_0068263 |
| hsa_circ_00714 | hsa_circ_00682 |

| | |
|---|---|
| 94 | 66 |
| hsa_circ_0071508 | hsa_circ_0068267 |
| hsa_circ_0071511 | hsa_circ_0068269 |
| hsa_circ_0071512 | hsa_circ_0068307 |
| hsa_circ_0071513 | hsa_circ_0068314 |
| hsa_circ_0071514 | hsa_circ_0068315 |
| hsa_circ_0071515 | hsa_circ_0068313 |
| hsa_circ_0071558 | hsa_circ_0068386 |
| hsa_circ_0071563 | hsa_circ_0068387 |
| hsa_circ_0071568 | hsa_circ_0068392 |
| hsa_circ_0071571 | hsa_circ_0068393 |
| hsa_circ_0071572 | hsa_circ_0068394 |
| hsa_circ_0071585 | hsa_circ_0068395 |
| hsa_circ_0071637 | hsa_circ_0068511 |
| hsa_c | hsa_c |

| | |
|---|---|
| irc_0071662 | irc_0068529 |
| hsa_circ_0071743 | hsa_circ_0068528 |
| hsa_circ_0071745 | hsa_circ_0068533 |
| hsa_circ_0071871 | hsa_circ_0068535 |
| hsa_circ_0008136 | hsa_circ_0068536 |
| hsa_circ_0017100 | hsa_circ_0068538 |
| hsa_circ_0072168 | hsa_circ_0068539 |
| hsa_circ_0072169 | hsa_circ_0068541 |
| hsa_circ_0072170 | hsa_circ_0007863 |
| hsa_circ_0072171 | hsa_circ_0005447 |
| hsa_circ_0072172 | hsa_circ_0068586 |
| hsa_circ_0072175 | hsa_circ_0068589 |
| hsa_circ_0072176 | hsa_circ_0009937 |
| hsa_circ_00721 | hsa_circ_00685 |

| | |
|---|---|
| 81 | 91 |
| hsa_circ_007218 2 | hsa_circ_006859 2 |
| hsa_circ_007218 8 | hsa_circ_006859 3 |
| hsa_circ_001898 | hsa_circ_006859 4 |
| hsa_circ_007220 7 | hsa_circ_006867 8 |
| hsa_circ_007220 8 | hsa_circ_006867 9 |
| hsa_circ_007220 9 | hsa_circ_006878 1 |
| hsa_circ_007229 8 | hsa_circ_006879 0 |
| hsa_circ_007229 9 | hsa_circ_006879 2 |
| hsa_circ_007230 0 | hsa_circ_006879 3 |
| hsa_circ_007231 7 | hsa_circ_006882 8 |
| hsa_circ_007232 0 | hsa_circ_006882 9 |
| hsa_circ_007232 4 | hsa_circ_006883 0 |
| hsa_circ_007232 5 | hsa_circ_006883 1 |
| hsa_c | hsa_c |

| | |
|---|---|
| irc_0072327 | irc_0068885 |
| hsa_circ_0072328 | hsa_circ_0068882 |
| hsa_circ_0072330 | hsa_circ_0068952 |
| hsa_circ_0072331 | hsa_circ_0069158 |
| hsa_circ_0072332 | hsa_circ_0069159 |
| hsa_circ_0072333 | hsa_circ_0069161 |
| hsa_circ_0072366 | hsa_circ_0069162 |
| hsa_circ_0072367 | hsa_circ_0069157 |
| hsa_circ_0072368 | hsa_circ_0004173 |
| hsa_circ_0072369 | hsa_circ_0069223 |
| hsa_circ_0072381 | hsa_circ_0069222 |
| hsa_circ_0072382 | hsa_circ_0069237 |
| hsa_circ_0072383 | hsa_circ_0008003 |
| hsa_circ_0072484 | hsa_circ_0009006 |

| | |
|---|---|
| hsa_circ_0072487 | hsa_circ_0069241 |
| hsa_circ_0072488 | hsa_circ_0069242 |
| hsa_circ_0072599 | hsa_circ_0069243 |
| hsa_circ_0072600 | hsa_circ_0069244 |
| hsa_circ_0072791 | hsa_circ_0069319 |
| hsa_circ_0072792 | hsa_circ_0069401 |
| hsa_circ_0072883 | hsa_circ_0069443 |
| hsa_circ_0072884 | hsa_circ_0069444 |
| hsa_circ_0072886 | hsa_circ_0069445 |
| hsa_circ_0072887 | hsa_circ_0069446 |
| hsa_circ_0072888 | hsa_circ_0069447 |
| hsa_circ_0072890 | hsa_circ_0069448 |
| hsa_circ_0072891 | hsa_circ_0069449 |
| hsa_circ_00728 | hsa_circ_00694 |

| | |
|---|---|
| 92 | 41 |
| hsa_circ_0072895 | hsa_circ_0069458 |
| hsa_circ_0072894 | hsa_circ_0069499 |
| hsa_circ_0072896 | hsa_circ_0069500 |
| hsa_circ_0072897 | hsa_circ_0069501 |
| hsa_circ_0072907 | hsa_circ_0069502 |
| hsa_circ_0072951 | hsa_circ_0069503 |
| hsa_circ_0006859 | hsa_circ_0069505 |
| hsa_circ_0073010 | hsa_circ_0069507 |
| hsa_circ_0073011 | hsa_circ_0069508 |
| hsa_circ_0073034 | hsa_circ_0069509 |
| hsa_circ_0073043 | hsa_circ_0008312 |
| hsa_circ_0002186 | hsa_circ_0069549 |
| hsa_circ_0073110 | hsa_circ_0069670 |
| hsa_c | hsa_c |

| | | | |
|---|---|---|---|
| | irc_0073111 | irc_0069671 | |
| | hsa_circ_0073113 | hsa_circ_0069672 | |
| | hsa_circ_0073114 | hsa_circ_0069673 | |
| | hsa_circ_0073123 | hsa_circ_0069674 | |
| | hsa_circ_0073124 | hsa_circ_0069675 | |
| | hsa_circ_0073125 | hsa_circ_0069676 | |
| | hsa_circ_0073126 | hsa_circ_0069677 | |
| | hsa_circ_0002260 | hsa_circ_0069678 | |
| | hsa_circ_0073135 | hsa_circ_0069858 | |
| | hsa_circ_0073139 | hsa_circ_0070041 | |
| | hsa_circ_0073140 | hsa_circ_0070091 | |
| | hsa_circ_0073141 | hsa_circ_0070094 | |
| | hsa_circ_0073144 | hsa_circ_0070104 | |
| | hsa_circ_0073145 | hsa_circ_0070105 | |

| | |
|---|---|
| hsa_circ_0073147 | hsa_circ_0070112 |
| hsa_circ_0073148 | hsa_circ_0070116 |
| hsa_circ_0073149 | hsa_circ_0070120 |
| hsa_circ_0073150 | hsa_circ_0070117 |
| hsa_circ_0073151 | hsa_circ_0070118 |
| hsa_circ_0073152 | hsa_circ_0070119 |
| hsa_circ_0073153 | hsa_circ_0070299 |
| hsa_circ_0073154 | hsa_circ_0070313 |
| hsa_circ_0073234 | hsa_circ_0070318 |
| hsa_circ_0073235 | hsa_circ_0070326 |
| hsa_circ_0073236 | hsa_circ_0070333 |
| hsa_circ_0073279 | hsa_circ_0070335 |
| hsa_circ_0073280 | hsa_circ_0070336 |
| hsa_circ_00732 | hsa_circ_00703 |

| | |
|---|---|
| 81 | 40 |
| hsa_circ_0073282 | hsa_circ_0070342 |
| hsa_circ_0073283 | hsa_circ_0070344 |
| hsa_circ_0073285 | hsa_circ_0008412 |
| hsa_circ_0073287 | hsa_circ_0070424 |
| hsa_circ_0073355 | hsa_circ_0070426 |
| hsa_circ_0073356 | hsa_circ_0070429 |
| hsa_circ_0073357 | hsa_circ_0070472 |
| hsa_circ_0073358 | hsa_circ_0003818 |
| hsa_circ_0073431 | hsa_circ_0004998 |
| hsa_circ_0073432 | hsa_circ_0070515 |
| hsa_circ_0073485 | hsa_circ_0070671 |
| hsa_circ_0073486 | hsa_circ_0070676 |
| hsa_circ_0073487 | hsa_circ_0070677 |
| hsa_c | hsa_c |

| | |
|---|---|
| irc_0073494 | irc_0070678 |
| hsa_circ_0003032 | hsa_circ_0070679 |
| hsa_circ_0003772 | hsa_circ_0070680 |
| hsa_circ_0006425 | hsa_circ_0070681 |
| hsa_circ_0073522 | hsa_circ_0070682 |
| hsa_circ_0073523 | hsa_circ_0070683 |
| hsa_circ_0073525 | hsa_circ_0070737 |
| hsa_circ_0073527 | hsa_circ_0070828 |
| hsa_circ_0073528 | hsa_circ_0070843 |
| hsa_circ_0073544 | hsa_circ_0070844 |
| hsa_circ_0073561 | hsa_circ_0070846 |
| hsa_circ_0073576 | hsa_circ_0070848 |
| hsa_circ_0073577 | hsa_circ_0070849 |
| hsa_circ_0004629 | hsa_circ_0070851 |

| | |
|---|---|
| hsa_circ_0009054 | hsa_circ_0070852 |
| hsa_circ_0073613 | hsa_circ_0070853 |
| hsa_circ_0073614 | hsa_circ_0070854 |
| hsa_circ_0073615 | hsa_circ_0070856 |
| hsa_circ_0073639 | hsa_circ_0070920 |
| hsa_circ_0073655 | hsa_circ_0070999 |
| hsa_circ_0073656 | hsa_circ_0071000 |
| hsa_circ_0073657 | hsa_circ_0071001 |
| hsa_circ_0073661 | hsa_circ_0007329 |
| hsa_circ_0073662 | hsa_circ_0071021 |
| hsa_circ_0073663 | hsa_circ_0071022 |
| hsa_circ_0073664 | hsa_circ_0071023 |
| hsa_circ_0073665 | hsa_circ_0071024 |
| hsa_circ_00737 | hsa_circ_00710 |

| | |
|---|---|
| 22 | 25 |
| hsa_circ_0073739 | hsa_circ_0071026 |
| hsa_circ_0073742 | hsa_circ_0071027 |
| hsa_circ_0073756 | hsa_circ_0071028 |
| hsa_circ_0073778 | hsa_circ_0006552 |
| hsa_circ_0073779 | hsa_circ_0071040 |
| hsa_circ_0073781 | hsa_circ_0071063 |
| hsa_circ_0073782 | hsa_circ_0071064 |
| hsa_circ_0073783 | hsa_circ_0071065 |
| hsa_circ_0073784 | hsa_circ_0071068 |
| hsa_circ_0073785 | hsa_circ_0071067 |
| hsa_circ_0073786 | hsa_circ_0071097 |
| hsa_circ_0073787 | hsa_circ_0071142 |
| hsa_circ_0073788 | hsa_circ_0071146 |
| hsa_c | hsa_c |

| | |
|---|---|
| irc_0073789 | irc_0071150 |
| hsa_circ_0073791 | hsa_circ_0071152 |
| hsa_circ_0073792 | hsa_circ_0071156 |
| hsa_circ_0074058 | hsa_circ_0006867 |
| hsa_circ_0006616 | hsa_circ_0071287 |
| hsa_circ_0074197 | hsa_circ_0071288 |
| hsa_circ_0007420 | hsa_circ_0071289 |
| hsa_circ_0074303 | hsa_circ_0071290 |
| hsa_circ_0074304 | hsa_circ_0006811 |
| hsa_circ_0074305 | hsa_circ_0071299 |
| hsa_circ_0074306 | hsa_circ_0071297 |
| hsa_circ_0074307 | hsa_circ_0071324 |
| hsa_circ_0074308 | hsa_circ_0071325 |
| hsa_circ_0074309 | hsa_circ_0071329 |

| | |
|---|---|
| hsa_circ_0074310 | hsa_circ_0071365 |
| hsa_circ_0074311 | hsa_circ_0071437 |
| hsa_circ_0074312 | hsa_circ_0071436 |
| hsa_circ_0074650 | hsa_circ_0071461 |
| hsa_circ_0074656 | hsa_circ_0071462 |
| hsa_circ_0074693 | hsa_circ_0071463 |
| hsa_circ_0074694 | hsa_circ_0004160 |
| hsa_circ_0074696 | hsa_circ_0004496 |
| hsa_circ_0074697 | hsa_circ_0004540 |
| hsa_circ_0074698 | hsa_circ_0005333 |
| hsa_circ_0074699 | hsa_circ_0071466 |
| hsa_circ_0074700 | hsa_circ_0002942 |
| hsa_circ_0074755 | hsa_circ_0071507 |
| hsa_circ_0748 | hsa_circ_0715 |

| | |
|---|---|
| 37 | 08 |
| hsa_circ_0074856 | hsa_circ_0071509 |
| hsa_circ_0074984 | hsa_circ_0071510 |
| hsa_circ_0075041 | hsa_circ_0071511 |
| hsa_circ_0075062 | hsa_circ_0071512 |
| hsa_circ_0075063 | hsa_circ_0071513 |
| hsa_circ_0075064 | hsa_circ_0071528 |
| hsa_circ_0075094 | hsa_circ_0071529 |
| hsa_circ_0075095 | hsa_circ_0071530 |
| hsa_circ_0075097 | hsa_circ_0071531 |
| hsa_circ_001525 | hsa_circ_0071532 |
| hsa_circ_0075111 | hsa_circ_0071557 |
| hsa_circ_0075112 | hsa_circ_0071558 |
| hsa_circ_0075114 | hsa_circ_0071562 |
| hsa_c | hsa_c |

| | |
|---|---|
| irc_0075148 | irc_0071563 |
| hsa_circ_0075150 | hsa_circ_0071568 |
| hsa_circ_0075151 | hsa_circ_0071588 |
| hsa_circ_0075152 | hsa_circ_0071592 |
| hsa_circ_0075153 | hsa_circ_0071593 |
| hsa_circ_0075189 | hsa_circ_0071595 |
| hsa_circ_0075327 | hsa_circ_0071596 |
| hsa_circ_0075328 | hsa_circ_0071598 |
| hsa_circ_0075329 | hsa_circ_0071599 |
| hsa_circ_0075330 | hsa_circ_0071603 |
| hsa_circ_0075331 | hsa_circ_0071602 |
| hsa_circ_0075466 | hsa_circ_0071604 |
| hsa_circ_0075467 | hsa_circ_0071625 |
| hsa_circ_0075468 | hsa_circ_0071626 |

| | |
|---|---|
| hsa_circ_0075469 | hsa_circ_0071629 |
| hsa_circ_0075505 | hsa_circ_0071631 |
| hsa_circ_0075506 | hsa_circ_0071632 |
| hsa_circ_0075507 | hsa_circ_0071637 |
| hsa_circ_0075592 | hsa_circ_0071683 |
| hsa_circ_0075632 | hsa_circ_0071685 |
| hsa_circ_0075633 | hsa_circ_0071743 |
| hsa_circ_0075634 | hsa_circ_0071744 |
| hsa_circ_0075640 | hsa_circ_0071745 |
| hsa_circ_0075684 | hsa_circ_0071873 |
| hsa_circ_0075847 | hsa_circ_0072106 |
| hsa_circ_0075907 | hsa_circ_0004807 |
| hsa_circ_0075932 | hsa_circ_0072118 |
| hsa_circ_00759 | hsa_circ_00721 |

| | |
|---|---|
| 44 | 19 |
| hsa_circ_0076149 | hsa_circ_0072120 |
| hsa_circ_0076150 | hsa_circ_0072121 |
| hsa_circ_0076151 | hsa_circ_0072122 |
| hsa_circ_0076192 | hsa_circ_0072123 |
| hsa_circ_0076415 | hsa_circ_0072124 |
| hsa_circ_0076704 | hsa_circ_0072125 |
| hsa_circ_0076705 | hsa_circ_0072149 |
| hsa_circ_0076706 | hsa_circ_0072152 |
| hsa_circ_0076707 | hsa_circ_0072209 |
| hsa_circ_0076785 | hsa_circ_0072211 |
| hsa_circ_0076786 | hsa_circ_0072212 |
| hsa_circ_0076787 | hsa_circ_0072213 |
| hsa_circ_0076788 | hsa_circ_0072214 |
| hsa_c | hsa_c |

| | |
|---|---|
| irc_0076789 | irc_0072366 |
| hsa_circ_0076790 | hsa_circ_0072367 |
| hsa_circ_0076791 | hsa_circ_0072368 |
| hsa_circ_0076867 | hsa_circ_0072369 |
| hsa_circ_0076868 | hsa_circ_0008621 |
| hsa_circ_0076869 | hsa_circ_0072382 |
| hsa_circ_0076870 | hsa_circ_0072383 |
| hsa_circ_0076871 | hsa_circ_0072385 |
| hsa_circ_0076872 | hsa_circ_0072386 |
| hsa_circ_0076962 | hsa_circ_0072387 |
| hsa_circ_0077028 | hsa_circ_0072388 |
| hsa_circ_0077032 | hsa_circ_0072389 |
| hsa_circ_0077042 | hsa_circ_0072390 |
| hsa_circ_0077047 | hsa_circ_0072391 |

| | |
|---|---|
| hsa_circ_0077054 | hsa_circ_0072401 |
| hsa_circ_0077058 | hsa_circ_0072402 |
| hsa_circ_0077059 | hsa_circ_0072403 |
| hsa_circ_0077060 | hsa_circ_0072404 |
| hsa_circ_0077094 | hsa_circ_0072405 |
| hsa_circ_0077233 | hsa_circ_0072410 |
| hsa_circ_0077234 | hsa_circ_0072412 |
| hsa_circ_0077266 | hsa_circ_0072414 |
| hsa_circ_0077290 | hsa_circ_0072415 |
| hsa_circ_0077300 | hsa_circ_0072417 |
| hsa_circ_0077301 | hsa_circ_0072465 |
| hsa_circ_0077302 | hsa_circ_0072509 |
| hsa_circ_0077303 | hsa_circ_0072552 |
| hsa_circ_00773 | hsa_circ_00725 |

| | |
|---|---|
| 37 | 53 |
| hsa_circ_0077338 | hsa_circ_0072574 |
| hsa_circ_0077339 | hsa_circ_0072573 |
| hsa_circ_0077340 | hsa_circ_0072585 |
| hsa_circ_0077342 | hsa_circ_0072586 |
| hsa_circ_0077343 | hsa_circ_0072652 |
| hsa_circ_0077344 | hsa_circ_0072704 |
| hsa_circ_0077345 | hsa_circ_0003174 |
| hsa_circ_0077346 | hsa_circ_0003801 |
| hsa_circ_0077347 | hsa_circ_0004932 |
| hsa_circ_0077348 | hsa_circ_0005146 |
| hsa_circ_0077325 | hsa_circ_0007498 |
| hsa_circ_0077326 | hsa_circ_0008723 |
| hsa_circ_0077327 | hsa_circ_0005896 |
| hsa_c | |

| | |
|---|---|
| irc_0077328 | hsa_circ_0007025 |
| hsa_circ_0077330 | hsa_circ_0007447 |
| hsa_circ_0077331 | hsa_circ_0072804 |
| hsa_circ_0077332 | hsa_circ_0072827 |
| hsa_circ_0077333 | hsa_circ_0072829 |
| hsa_circ_0077334 | hsa_circ_0005159 |
| hsa_circ_0077336 | hsa_circ_0004545 |
| hsa_circ_0077310 | hsa_circ_0072810 |
| hsa_circ_0077311 | hsa_circ_0072811 |
| hsa_circ_0077315 | hsa_circ_0072813 |
| hsa_circ_0077316 | hsa_circ_0072846 |
| hsa_circ_0077318 | hsa_circ_0072907 |
| hsa_circ_0077319 | hsa_circ_0072964 |
| hsa_circ_00773 | hsa_circ_00729 |

| | |
|---|---|
| 20 | 70 |
| hsa_circ_0077321 | hsa_circ_0072971 |
| hsa_circ_0077322 | hsa_circ_0072973 |
| hsa_circ_0077324 | hsa_circ_0072974 |
| hsa_circ_0077434 | hsa_circ_0072975 |
| hsa_circ_0077581 | hsa_circ_0072976 |
| hsa_circ_0077582 | hsa_circ_0072977 |
| hsa_circ_0077642 | hsa_circ_0072979 |
| hsa_circ_0077644 | hsa_circ_0072990 |
| hsa_circ_0077647 | hsa_circ_0006859 |
| hsa_circ_0077649 | hsa_circ_0073010 |
| hsa_circ_0077651 | hsa_circ_0073011 |
| hsa_circ_0077652 | hsa_circ_0073031 |
| hsa_circ_0077665 | hsa_circ_0073033 |
| hsa_c | hsa_c |

| | |
|---|---|
| irc_0 0776 66 | irc_0 0730 34 |
| hsa_c irc_0 0776 68 | hsa_c irc_0 0730 42 |
| hsa_c irc_0 0777 53 | hsa_c irc_0 0730 66 |
| hsa_c irc_0 0777 55 | hsa_c irc_0 0731 35 |
| hsa_c irc_0 0778 05 | hsa_c irc_0 0731 60 |
| hsa_c irc_0 0778 23 | hsa_c irc_0 0731 61 |
| hsa_c irc_0 0778 98 | hsa_c irc_0 0732 25 |
| hsa_c irc_0 0779 53 | hsa_c irc_0 0732 27 |
| hsa_c irc_0 0781 02 | hsa_c irc_0 0732 31 |
| hsa_c irc_0 0781 12 | hsa_c irc_0 0732 32 |
| hsa_c irc_0 0781 33 | hsa_c irc_0 0035 61 |
| hsa_c irc_0 0782 20 | hsa_c irc_0 0734 32 |
| hsa_c irc_0 0782 21 | hsa_c irc_0 0734 36 |
| hsa_c irc_0 0782 22 | hsa_c irc_0 0734 42 |

| | |
|---|---|
| hsa_circ_0078299 | hsa_circ_0073443 |
| hsa_circ_0078382 | hsa_circ_0073445 |
| hsa_circ_0002016 | hsa_circ_0073450 |
| hsa_circ_0078572 | hsa_circ_0073452 |
| hsa_circ_0078573 | hsa_circ_0073453 |
| hsa_circ_0078575 | hsa_circ_0073485 |
| hsa_circ_0078576 | hsa_circ_0073486 |
| hsa_circ_0078578 | hsa_circ_0073541 |
| hsa_circ_0078579 | hsa_circ_0073542 |
| hsa_circ_0078582 | hsa_circ_0073543 |
| hsa_circ_0078583 | hsa_circ_0073544 |
| hsa_circ_0078584 | hsa_circ_0073549 |
| hsa_circ_0078585 | hsa_circ_0073550 |

| | |
|---|---|
| hsa_circ_0078587 | hsa_circ_0073551 |
| hsa_circ_0078588 | hsa_circ_0073552 |
| hsa_circ_0078589 | hsa_circ_0073554 |
| hsa_circ_0078590 | hsa_circ_0005381 |
| hsa_circ_0078591 | hsa_circ_0073656 |
| hsa_circ_0078592 | hsa_circ_0073657 |
| hsa_circ_0078593 | hsa_circ_0073663 |
| hsa_circ_0078641 | hsa_circ_0073664 |
| hsa_circ_0078684 | hsa_circ_0073665 |
| hsa_circ_0078687 | hsa_circ_0073666 |
| hsa_circ_0078688 | hsa_circ_0073667 |
| hsa_circ_0079071 | hsa_circ_0073668 |
| hsa_circ_0079153 | hsa_circ_0073669 |
| hsa_circ_0048 | hsa_circ_0736 |

| 69 | 70 |
|---|---|
| hsa_circ_0079161 | hsa_circ_0073756 |
| hsa_circ_0079162 | hsa_circ_0073776 |
| hsa_circ_0079163 | hsa_circ_0073777 |
| hsa_circ_0079164 | hsa_circ_0073778 |
| hsa_circ_0079165 | hsa_circ_0073838 |
| hsa_circ_0007319 | hsa_circ_0073839 |
| hsa_circ_0079503 | hsa_circ_0073840 |
| hsa_circ_0001971 | hsa_circ_0073841 |
| hsa_circ_0003825 | hsa_circ_0073842 |
| hsa_circ_0005251 | hsa_circ_0073843 |
| hsa_circ_0006307 | hsa_circ_0073844 |
| hsa_circ_0008951 | hsa_circ_0073845 |
| hsa_circ_0079561 | hsa_circ_0073846 |
| hsa_c | hsa_c |

| | |
|---|---|
| irc_0079562 | irc_0073847 |
| hsa_circ_0079563 | hsa_circ_0073871 |
| hsa_circ_0079564 | hsa_circ_0073906 |
| hsa_circ_0079711 | hsa_circ_0074012 |
| hsa_circ_0079712 | hsa_circ_0074014 |
| hsa_circ_0079719 | hsa_circ_0074016 |
| hsa_circ_0079721 | hsa_circ_0074017 |
| hsa_circ_0079722 | hsa_circ_0074028 |
| hsa_circ_0079726 | hsa_circ_0074085 |
| hsa_circ_0079727 | hsa_circ_0074087 |
| hsa_circ_0079728 | hsa_circ_0074089 |
| hsa_circ_0079729 | hsa_circ_0074187 |
| hsa_circ_0079763 | hsa_circ_0008595 |
| hsa_circ_0079864 | hsa_circ_0074180 |

| | |
|---|---|
| hsa_circ_0079865 | hsa_circ_0074184 |
| hsa_circ_0079942 | hsa_circ_0074240 |
| hsa_circ_0002033 | hsa_circ_0074279 |
| hsa_circ_0080214 | hsa_circ_0074280 |
| hsa_circ_0080233 | hsa_circ_0074281 |
| hsa_circ_0003086 | hsa_circ_0074294 |
| hsa_circ_0007469 | hsa_circ_0074329 |
| hsa_circ_0008770 | hsa_circ_0074330 |
| hsa_circ_0005979 | hsa_circ_0074332 |
| hsa_circ_0006442 | hsa_circ_0074334 |
| hsa_circ_001947 | hsa_circ_0074335 |
| hsa_circ_0080355 | hsa_circ_0074336 |
| hsa_circ_0080357 | hsa_circ_0074354 |
| hsa_circ_00804 | hsa_circ_00743 |

| | |
|---|---|
| 48 | 55 |
| hsa_circ_0080450 | hsa_circ_0074356 |
| hsa_circ_0080456 | hsa_circ_0074417 |
| hsa_circ_0008167 | hsa_circ_0074418 |
| hsa_circ_0003317 | hsa_circ_0074419 |
| hsa_circ_0006821 | hsa_circ_0074427 |
| hsa_circ_0009129 | hsa_circ_0074428 |
| hsa_circ_0006372 | hsa_circ_0074420 |
| hsa_circ_0008576 | hsa_circ_0074421 |
| hsa_circ_0080478 | hsa_circ_0074422 |
| hsa_circ_0080479 | hsa_circ_0074423 |
| hsa_circ_0080482 | hsa_circ_0074424 |
| hsa_circ_0080485 | hsa_circ_0074425 |
| hsa_circ_0080486 | hsa_circ_0074447 |
| hsa_c | hsa_c |

| | |
|---|---|
| irc_0080487 | irc_0074448 |
| hsa_circ_0080489 | hsa_circ_0074449 |
| hsa_circ_0080490 | hsa_circ_0074450 |
| hsa_circ_0080661 | hsa_circ_0074451 |
| hsa_circ_0080684 | hsa_circ_0074452 |
| hsa_circ_0080685 | hsa_circ_0074493 |
| hsa_circ_0080686 | hsa_circ_0074494 |
| hsa_circ_0006766 | hsa_circ_0074495 |
| hsa_circ_0008422 | hsa_circ_0074571 |
| hsa_circ_0080916 | hsa_circ_0074572 |
| hsa_circ_0080977 | hsa_circ_0074695 |
| hsa_circ_0081020 | hsa_circ_0074696 |
| hsa_circ_0081030 | hsa_circ_0074697 |
| hsa_circ_0081174 | hsa_circ_0074698 |

| | |
|---|---|
| hsa_circ_0081179 | hsa_circ_0074699 |
| hsa_circ_0081227 | hsa_circ_0074700 |
| hsa_circ_0081228 | hsa_circ_0074801 |
| hsa_circ_0081229 | hsa_circ_0074803 |
| hsa_circ_0081230 | hsa_circ_0074804 |
| hsa_circ_0081231 | hsa_circ_0074838 |
| hsa_circ_0081232 | hsa_circ_0074856 |
| hsa_circ_0081233 | hsa_circ_0074901 |
| hsa_circ_0081234 | hsa_circ_0074908 |
| hsa_circ_0081235 | hsa_circ_0074909 |
| hsa_circ_0081318 | hsa_circ_0074911 |
| hsa_circ_002103 | hsa_circ_0074912 |
| hsa_circ_0081526 | hsa_circ_0074918 |
| hsa_circ_00815 | hsa_circ_00749 |

| | |
|---|---|
| 29 | 19 |
| hsa_circ_0007639 | hsa_circ_0074922 |
| hsa_circ_001182 | hsa_circ_0074923 |
| hsa_circ_0081755 | hsa_circ_0074924 |
| hsa_circ_0081756 | hsa_circ_0002745 |
| hsa_circ_0081757 | hsa_circ_0003696 |
| hsa_circ_0081759 | hsa_circ_0004527 |
| hsa_circ_0081760 | hsa_circ_0074974 |
| hsa_circ_0081761 | hsa_circ_0074975 |
| hsa_circ_0081815 | hsa_circ_0074976 |
| hsa_circ_0081876 | hsa_circ_0074979 |
| hsa_circ_0003932 | hsa_circ_0074980 |
| hsa_circ_0081907 | hsa_circ_0074981 |
| hsa_circ_0081918 | hsa_circ_0074982 |
| hsa_c | hsa_c |

| | |
|---|---|
| irc_0081925 | irc_0075063 |
| hsa_circ_0081926 | hsa_circ_0075064 |
| hsa_circ_0081934 | hsa_circ_0002171 |
| hsa_circ_0081938 | hsa_circ_0075092 |
| hsa_circ_0081939 | hsa_circ_0075147 |
| hsa_circ_0081940 | hsa_circ_0075187 |
| hsa_circ_0081941 | hsa_circ_0075188 |
| hsa_circ_0081942 | hsa_circ_0075189 |
| hsa_circ_0081968 | hsa_circ_0075190 |
| hsa_circ_0081969 | hsa_circ_0075191 |
| hsa_circ_0081970 | hsa_circ_0075305 |
| hsa_circ_0082004 | hsa_circ_0075306 |
| hsa_circ_0082005 | hsa_circ_0075307 |
| hsa_circ_0082006 | hsa_circ_0075371 |

| | |
|---|---|
| hsa_circ_0082007 | hsa_circ_0075407 |
| hsa_circ_0006252 | hsa_circ_0075408 |
| hsa_circ_0082103 | hsa_circ_0075466 |
| hsa_circ_0082107 | hsa_circ_0075467 |
| hsa_circ_0082108 | hsa_circ_0075468 |
| hsa_circ_0082111 | hsa_circ_0075469 |
| hsa_circ_0082112 | hsa_circ_0075500 |
| hsa_circ_0082114 | hsa_circ_0075549 |
| hsa_circ_0082115 | hsa_circ_0075640 |
| hsa_circ_0082118 | hsa_circ_0075676 |
| hsa_circ_0082119 | hsa_circ_0007370 |
| hsa_circ_0082120 | hsa_circ_0075702 |
| hsa_circ_0082121 | hsa_circ_0075703 |
| hsa_circ_00821 | hsa_circ_00757 |

| | |
|---|---|
| 22 | 04 |
| hsa_circ_0082123 | hsa_circ_0075705 |
| hsa_circ_0082125 | hsa_circ_0075706 |
| hsa_circ_0082126 | hsa_circ_0075707 |
| hsa_circ_0082127 | hsa_circ_0075710 |
| hsa_circ_0082128 | hsa_circ_0075711 |
| hsa_circ_0082129 | hsa_circ_0075714 |
| hsa_circ_0082130 | hsa_circ_0075785 |
| hsa_circ_0082132 | hsa_circ_0075812 |
| hsa_circ_0082133 | hsa_circ_0075815 |
| hsa_circ_0082134 | hsa_circ_000744 |
| hsa_circ_0082135 | hsa_circ_0075893 |
| hsa_circ_0082136 | hsa_circ_0075895 |
| hsa_circ_0082137 | hsa_circ_0075921 |
| hsa_c | hsa_c |

| | |
|---|---|
| irc_0082138 | irc_0075922 |
| hsa_circ_0082139 | hsa_circ_0075923 |
| hsa_circ_0082140 | hsa_circ_0075938 |
| hsa_circ_0082141 | hsa_circ_0075943 |
| hsa_circ_0082142 | hsa_circ_0075953 |
| hsa_circ_0082143 | hsa_circ_0075960 |
| hsa_circ_0082144 | hsa_circ_0075974 |
| hsa_circ_0082145 | hsa_circ_0075975 |
| hsa_circ_0082146 | hsa_circ_0075976 |
| hsa_circ_0082147 | hsa_circ_0075977 |
| hsa_circ_0082369 | hsa_circ_0075978 |
| hsa_circ_0082371 | hsa_circ_0075980 |
| hsa_circ_0082372 | hsa_circ_0075983 |
| hsa_circ_0082373 | hsa_circ_0075984 |

| | |
|---|---|
| hsa_circ_0082374 | hsa_circ_0075985 |
| hsa_circ_0082375 | hsa_circ_0075986 |
| hsa_circ_0082376 | hsa_circ_0076042 |
| hsa_circ_0082561 | hsa_circ_0076050 |
| hsa_circ_0082562 | hsa_circ_0076051 |
| hsa_circ_0082621 | hsa_circ_0076120 |
| hsa_circ_0082665 | hsa_circ_0076121 |
| hsa_circ_0082666 | hsa_circ_0076122 |
| hsa_circ_0082701 | hsa_circ_0076194 |
| hsa_circ_0082702 | hsa_circ_0005407 |
| hsa_circ_0082703 | hsa_circ_0076293 |
| hsa_circ_002135 | hsa_circ_0076294 |
| hsa_circ_0082785 | hsa_circ_0076329 |
| hsa_circ_00827 | hsa_circ_00763 |

| | |
|---|---|
| 86 | 72 |
| hsa_circ_0082843 | hsa_circ_0076513 |
| hsa_circ_0082899 | hsa_circ_0076514 |
| hsa_circ_0083091 | hsa_circ_0076515 |
| hsa_circ_0083095 | hsa_circ_0076516 |
| hsa_circ_0083104 | hsa_circ_0076517 |
| hsa_circ_0083120 | hsa_circ_0076518 |
| hsa_circ_0083170 | hsa_circ_0076519 |
| hsa_circ_0083174 | hsa_circ_0076520 |
| hsa_circ_0083181 | hsa_circ_0076532 |
| hsa_circ_0083203 | hsa_circ_0076533 |
| hsa_circ_0083469 | hsa_circ_0076540 |
| hsa_circ_0083471 | hsa_circ_0076542 |
| hsa_circ_0083472 | hsa_circ_0076543 |
| hsa_c | hsa_c |

| | |
|---|---|
| irc_0083576 | irc_0076544 |
| hsa_circ_0083575 | hsa_circ_0076545 |
| hsa_circ_0083574 | hsa_circ_0076546 |
| hsa_circ_0083577 | hsa_circ_0076547 |
| hsa_circ_0002131 | hsa_circ_0076549 |
| hsa_circ_0083743 | hsa_circ_0076550 |
| hsa_circ_0083745 | hsa_circ_0076551 |
| hsa_circ_0083749 | hsa_circ_0076608 |
| hsa_circ_0083751 | hsa_circ_0076629 |
| hsa_circ_0083752 | hsa_circ_0076862 |
| hsa_circ_0083796 | hsa_circ_0076867 |
| hsa_circ_0083797 | hsa_circ_0077071 |
| hsa_circ_0083800 | hsa_circ_0077110 |
| hsa_circ_0083809 | hsa_circ_0002041 |

| | |
|---|---|
| hsa_circ_0083942 | hsa_circ_0077173 |
| hsa_circ_0083968 | hsa_circ_0077175 |
| hsa_circ_0084059 | hsa_circ_0077177 |
| hsa_circ_0084060 | hsa_circ_0077178 |
| hsa_circ_0084061 | hsa_circ_0077179 |
| hsa_circ_0084067 | hsa_circ_0077202 |
| hsa_circ_0084186 | hsa_circ_0077204 |
| hsa_circ_0084187 | hsa_circ_0077222 |
| hsa_circ_0084647 | hsa_circ_0077223 |
| hsa_circ_0084685 | hsa_circ_0077289 |
| hsa_circ_0084791 | hsa_circ_0077382 |
| hsa_circ_0084793 | hsa_circ_0077385 |
| hsa_circ_0084794 | hsa_circ_0077388 |
| hsa_circ_00848 | hsa_circ_00774 |

| | |
|---|---|
| 45 | 12 |
| hsa_circ_0084847 | hsa_circ_0077413 |
| hsa_circ_0084848 | hsa_circ_0008321 |
| hsa_circ_0084849 | hsa_circ_0077441 |
| hsa_circ_0084850 | hsa_circ_0077444 |
| hsa_circ_0084851 | hsa_circ_0077445 |
| hsa_circ_0084852 | hsa_circ_0077446 |
| hsa_circ_0084853 | hsa_circ_0077451 |
| hsa_circ_0005934 | hsa_circ_0077452 |
| hsa_circ_0085179 | hsa_circ_0077453 |
| hsa_circ_0085337 | hsa_circ_0077454 |
| hsa_circ_0085455 | hsa_circ_0077458 |
| hsa_circ_0085457 | hsa_circ_0077507 |
| hsa_circ_0085461 | hsa_circ_0077508 |
| hsa_c | hsa_c |

| | |
|---|---|
| irc_0085462 | irc_0077525 |
| hsa_circ_0085464 | hsa_circ_0077529 |
| hsa_circ_0085466 | hsa_circ_0077593 |
| hsa_circ_0085467 | hsa_circ_0077596 |
| hsa_circ_0085468 | hsa_circ_0077598 |
| hsa_circ_0085469 | hsa_circ_0077599 |
| hsa_circ_0085483 | hsa_circ_0077600 |
| hsa_circ_0085484 | hsa_circ_0077642 |
| hsa_circ_0085489 | hsa_circ_0077644 |
| hsa_circ_0085541 | hsa_circ_0077647 |
| hsa_circ_0006447 | hsa_circ_0077649 |
| hsa_circ_0085645 | hsa_circ_0077651 |
| hsa_circ_0085708 | hsa_circ_0077652 |
| hsa_circ_0085709 | hsa_circ_0077672 |

| | |
|---|---|
| hsa_circ_0085710 | hsa_circ_0077673 |
| hsa_circ_0002062 | hsa_circ_0077674 |
| hsa_circ_0005259 | hsa_circ_0077678 |
| hsa_circ_0085775 | hsa_circ_0077679 |
| hsa_circ_0085777 | hsa_circ_0077680 |
| hsa_circ_0085779 | hsa_circ_0077681 |
| hsa_circ_0085780 | hsa_circ_0077765 |
| hsa_circ_0085781 | hsa_circ_0077766 |
| hsa_circ_0085782 | hsa_circ_0077805 |
| hsa_circ_0085783 | hsa_circ_0077806 |
| hsa_circ_0085784 | hsa_circ_0077807 |
| hsa_circ_0085785 | hsa_circ_0077808 |
| hsa_circ_0085872 | hsa_circ_0077900 |
| hsa_circ_00858 | hsa_circ_00779 |

| | |
|---|---|
| 73 | 59 |
| hsa_circ_0085874 | hsa_circ_0077960 |
| hsa_circ_0085875 | hsa_circ_0077961 |
| hsa_circ_0085876 | hsa_circ_0077962 |
| hsa_circ_0085897 | hsa_circ_0077963 |
| hsa_circ_0085898 | hsa_circ_0077964 |
| hsa_circ_0086034 | hsa_circ_0077965 |
| hsa_circ_0086036 | hsa_circ_0006071 |
| hsa_circ_0086038 | hsa_circ_0078031 |
| hsa_circ_0086039 | hsa_circ_0078049 |
| hsa_circ_0086179 | hsa_circ_0078065 |
| hsa_circ_0086180 | hsa_circ_0078066 |
| hsa_circ_0086181 | hsa_circ_0078068 |
| hsa_circ_0086182 | hsa_circ_0078069 |
| hsa_c | hsa_c |

| | |
|---|---|
| irc_0086197 | irc_0078070 |
| hsa_circ_0086198 | hsa_circ_0078071 |
| hsa_circ_0086199 | hsa_circ_0078155 |
| hsa_circ_0086243 | hsa_circ_0078156 |
| hsa_circ_0086244 | hsa_circ_0078165 |
| hsa_circ_0005890 | hsa_circ_0078166 |
| hsa_circ_0086289 | hsa_circ_0078170 |
| hsa_circ_0086290 | hsa_circ_0078174 |
| hsa_circ_0086616 | hsa_circ_0078177 |
| hsa_circ_0086617 | hsa_circ_0078179 |
| hsa_circ_0086618 | hsa_circ_0078180 |
| hsa_circ_0086619 | hsa_circ_0078181 |
| hsa_circ_0086620 | hsa_circ_0078262 |
| hsa_circ_0086621 | hsa_circ_0078312 |

| | |
|---|---|
| hsa_circ_0086632 | hsa_circ_0078313 |
| hsa_circ_0086633 | hsa_circ_0078338 |
| hsa_circ_0086634 | hsa_circ_0078341 |
| hsa_circ_0086635 | hsa_circ_0078342 |
| hsa_circ_0086636 | hsa_circ_0078357 |
| hsa_circ_0086639 | hsa_circ_0078358 |
| hsa_circ_0086642 | hsa_circ_0078359 |
| hsa_circ_0086643 | hsa_circ_0078360 |
| hsa_circ_0087010 | hsa_circ_0078382 |
| hsa_circ_0004398 | hsa_circ_0003004 |
| hsa_circ_0087040 | hsa_circ_0078437 |
| hsa_circ_0087041 | hsa_circ_0078438 |
| hsa_circ_0087044 | hsa_circ_0078439 |
| hsa_circ_00870 | hsa_circ_00785 |

| | |
|---|---|
| 46 | 73 |
| hsa_circ_0087047 | hsa_circ_0078575 |
| hsa_circ_0087048 | hsa_circ_0078576 |
| hsa_circ_0087050 | hsa_circ_0078578 |
| hsa_circ_0087051 | hsa_circ_0078579 |
| hsa_circ_0087052 | hsa_circ_0078582 |
| hsa_circ_0087053 | hsa_circ_0078584 |
| hsa_circ_0087054 | hsa_circ_0078585 |
| hsa_circ_0087055 | hsa_circ_0078587 |
| hsa_circ_0087056 | hsa_circ_0078588 |
| hsa_circ_0087154 | hsa_circ_0078603 |
| hsa_circ_0087155 | hsa_circ_0078641 |
| hsa_circ_0087156 | hsa_circ_0078647 |
| hsa_circ_0087157 | hsa_circ_0078684 |
| hsa_c | hsa_c |

| | |
|---|---|
| irc_008718 8 | irc_007876 9 |
| hsa_circ_008719 4 | hsa_circ_007877 0 |
| hsa_circ_008719 5 | hsa_circ_007877 1 |
| hsa_circ_008721 3 | hsa_circ_007877 3 |
| hsa_circ_008723 6 | hsa_circ_007905 1 |
| hsa_circ_008723 7 | hsa_circ_007905 2 |
| hsa_circ_008723 8 | hsa_circ_007905 3 |
| hsa_circ_008725 1 | hsa_circ_007905 7 |
| hsa_circ_008725 2 | hsa_circ_007905 8 |
| hsa_circ_008725 3 | hsa_circ_007905 9 |
| hsa_circ_000803 8 | hsa_circ_007906 0 |
| hsa_circ_008734 2 | hsa_circ_007906 1 |
| hsa_circ_008734 3 | hsa_circ_001717 |
| hsa_circ_008734 4 | hsa_circ_007909 0 |

| | |
|---|---|
| hsa_circ_0087345 | hsa_circ_0079107 |
| hsa_circ_0087346 | hsa_circ_0079109 |
| hsa_circ_0003453 | hsa_circ_0079111 |
| hsa_circ_0087363 | hsa_circ_0079112 |
| hsa_circ_0007162 | hsa_circ_0079113 |
| hsa_circ_0087382 | hsa_circ_0079114 |
| hsa_circ_0087383 | hsa_circ_0079115 |
| hsa_circ_0087384 | hsa_circ_0079116 |
| hsa_circ_0087386 | hsa_circ_0079142 |
| hsa_circ_0087387 | hsa_circ_0079150 |
| hsa_circ_0087388 | hsa_circ_0004961 |
| hsa_circ_0009 9 | hsa_circ_0007319 |
| hsa_circ_0005826 | hsa_circ_0079264 |
| hsa_circ_0065 | hsa_circ_0792 |

| | |
|---|---|
| 70 | 65 |
| hsa_circ_0002794 | hsa_circ_0079266 |
| hsa_circ_0007060 | hsa_circ_0079267 |
| hsa_circ_0087531 | hsa_circ_0079269 |
| hsa_circ_0087532 | hsa_circ_0079270 |
| hsa_circ_0087533 | hsa_circ_0079271 |
| hsa_circ_0087534 | hsa_circ_0079272 |
| hsa_circ_0087535 | hsa_circ_0079273 |
| hsa_circ_0087536 | hsa_circ_0006574 |
| hsa_circ_0087537 | hsa_circ_0079284 |
| hsa_circ_0087538 | hsa_circ_0079285 |
| hsa_circ_0087541 | hsa_circ_0079331 |
| hsa_circ_0087542 | hsa_circ_0079338 |
| hsa_circ_0087543 | hsa_circ_0079339 |
| hsa_c | hsa_c |

| | |
|---|---|
| irc_0087544 | irc_0079367 |
| hsa_circ_0087547 | hsa_circ_0079502 |
| hsa_circ_0087548 | hsa_circ_0079497 |
| hsa_circ_0087549 | hsa_circ_0004671 |
| hsa_circ_0087550 | hsa_circ_0005519 |
| hsa_circ_0087551 | hsa_circ_0079507 |
| hsa_circ_0087552 | hsa_circ_0079509 |
| hsa_circ_0003092 | hsa_circ_0079510 |
| hsa_circ_0087567 | hsa_circ_0079512 |
| hsa_circ_0087568 | hsa_circ_0079513 |
| hsa_circ_0087569 | hsa_circ_0079514 |
| hsa_circ_0087570 | hsa_circ_0079515 |
| hsa_circ_0087571 | hsa_circ_0079517 |
| hsa_circ_0087572 | hsa_circ_0079533 |

| | |
|---|---|
| hsa_circ_0087573 | hsa_circ_0079578 |
| hsa_circ_0008720 | hsa_circ_0079579 |
| hsa_circ_0005306 | hsa_circ_0079580 |
| hsa_circ_0087652 | hsa_circ_0079581 |
| hsa_circ_0087696 | hsa_circ_0079582 |
| hsa_circ_0087702 | hsa_circ_0079583 |
| hsa_circ_0087703 | hsa_circ_0079584 |
| hsa_circ_0087820 | hsa_circ_0079699 |
| hsa_circ_0087823 | hsa_circ_0079698 |
| hsa_circ_0087825 | hsa_circ_0079710 |
| hsa_circ_0087826 | hsa_circ_0079711 |
| hsa_circ_0087829 | hsa_circ_0079712 |
| hsa_circ_0087832 | hsa_circ_0079715 |
| hsa_circ_00878 | hsa_circ_00797 |

| | |
|---|---|
| 34 | 17 |
| hsa_circ_0087836 | hsa_circ_0079718 |
| hsa_circ_0087837 | hsa_circ_0079719 |
| hsa_circ_0087845 | hsa_circ_0079720 |
| hsa_circ_0088033 | hsa_circ_0079721 |
| hsa_circ_0088063 | hsa_circ_0079763 |
| hsa_circ_0088064 | hsa_circ_0079805 |
| hsa_circ_0088148 | hsa_circ_0079823 |
| hsa_circ_0088150 | hsa_circ_0006083 |
| hsa_circ_0088152 | hsa_circ_0080020 |
| hsa_circ_0088155 | hsa_circ_0080023 |
| hsa_circ_0088433 | hsa_circ_0080083 |
| hsa_circ_0088434 | hsa_circ_0080086 |
| hsa_circ_0088528 | hsa_circ_0080087 |
| hsa_c | hsa_c |

| | |
|---|---|
| irc_0009007 | irc_0080089 |
| hsa_circ_0088554 | hsa_circ_0080091 |
| hsa_circ_0088555 | hsa_circ_0080092 |
| hsa_circ_0088557 | hsa_circ_0080093 |
| hsa_circ_0088558 | hsa_circ_0080110 |
| hsa_circ_0088559 | hsa_circ_0080111 |
| hsa_circ_0088560 | hsa_circ_0080112 |
| hsa_circ_0088561 | hsa_circ_0080113 |
| hsa_circ_0088562 | hsa_circ_0080298 |
| hsa_circ_0088908 | hsa_circ_0007469 |
| hsa_circ_0088909 | hsa_circ_0007470 |
| hsa_circ_0088910 | hsa_circ_0003086 |
| hsa_circ_0088923 | hsa_circ_0005979 |
| hsa_circ_0088924 | hsa_circ_0006442 |

| | |
|---|---|
| hsa_circ_0088926 | hsa_circ_0007502 |
| hsa_circ_0088927 | hsa_circ_0006562 |
| hsa_circ_0088928 | hsa_circ_0080357 |
| hsa_circ_0088929 | hsa_circ_0080355 |
| hsa_circ_0088930 | hsa_circ_0009077 |
| hsa_circ_0088931 | hsa_circ_0006372 |
| hsa_circ_0088932 | hsa_circ_0008576 |
| hsa_circ_0088934 | hsa_circ_0080506 |
| hsa_circ_0088935 | hsa_circ_0005283 |
| hsa_circ_0088936 | hsa_circ_0008167 |
| hsa_circ_0088937 | hsa_circ_0003603 |
| hsa_circ_0088938 | hsa_circ_0006821 |
| hsa_circ_0088939 | hsa_circ_0009129 |
| hsa_circ_00889 | hsa_circ_00033 |

| | |
|---|---|
| 40 | 17 |
| hsa_circ_0088941 | hsa_circ_0080510 |
| hsa_circ_0088942 | hsa_circ_0080521 |
| hsa_circ_0088943 | hsa_circ_0080524 |
| hsa_circ_0088944 | hsa_circ_0080527 |
| hsa_circ_0088945 | hsa_circ_0080528 |
| hsa_circ_0088946 | hsa_circ_0080529 |
| hsa_circ_0088947 | hsa_circ_0080530 |
| hsa_circ_0089111 | hsa_circ_0008971 |
| hsa_circ_0089112 | hsa_circ_0006368 |
| hsa_circ_0089116 | hsa_circ_0006766 |
| hsa_circ_0089118 | hsa_circ_0080750 |
| hsa_circ_0089119 | hsa_circ_0080755 |
| hsa_circ_0089141 | hsa_circ_0080752 |
| hsa_c | hsa_c |

| | |
|---|---|
| irc_0089192 | irc_0080756 |
| hsa_circ_0089193 | hsa_circ_0080753 |
| hsa_circ_0006825 | hsa_circ_0080758 |
| hsa_circ_0089286 | hsa_circ_0080759 |
| hsa_circ_0089295 | hsa_circ_01324 |
| hsa_circ_0089300 | hsa_circ_0080773 |
| hsa_circ_0089524 | hsa_circ_0080774 |
| hsa_circ_0089526 | hsa_circ_0080775 |
| hsa_circ_0089527 | hsa_circ_0080776 |
| hsa_circ_0089629 | hsa_circ_0080777 |
| hsa_circ_0089630 | hsa_circ_0080778 |
| hsa_circ_0089631 | hsa_circ_0080807 |
| hsa_circ_0089632 | hsa_circ_0080821 |
| hsa_circ_0089633 | hsa_circ_0080822 |

| | |
|---|---|
| hsa_circ_0089634 | hsa_circ_0080823 |
| hsa_circ_0017363 | hsa_circ_0080825 |
| hsa_circ_0017530 | hsa_circ_0080861 |
| hsa_circ_0017562 | hsa_circ_0080863 |
| hsa_circ_0017563 | hsa_circ_0080865 |
| hsa_circ_0017564 | hsa_circ_0080866 |
| hsa_circ_0017571 | hsa_circ_0080880 |
| hsa_circ_0017572 | hsa_circ_0080902 |
| hsa_circ_0017611 | hsa_circ_0080963 |
| hsa_circ_0017905 | hsa_circ_0080977 |
| hsa_circ_0017920 | hsa_circ_0002497 |
| hsa_circ_0017921 | hsa_circ_0081025 |
| hsa_circ_0017922 | hsa_circ_0081166 |
| hsa_circ_00179 | hsa_circ_00811 |

| | |
|---|---|
| 23 | 79 |
| hsa_circ_0017924 | hsa_circ_0081201 |
| hsa_circ_0017925 | hsa_circ_0081202 |
| hsa_circ_001980 | hsa_circ_0081203 |
| hsa_circ_0018067 | hsa_circ_0081204 |
| hsa_circ_0018069 | hsa_circ_0081205 |
| hsa_circ_0018230 | hsa_circ_002138 |
| hsa_circ_0018231 | hsa_circ_0081212 |
| hsa_circ_0018232 | hsa_circ_0081230 |
| hsa_circ_0018239 | hsa_circ_0081232 |
| hsa_circ_0002461 | hsa_circ_0081235 |
| hsa_circ_0003547 | hsa_circ_0081236 |
| hsa_circ_0008028 | hsa_circ_0081237 |
| hsa_circ_0005911 | hsa_circ_0081239 |
| hsa_c | hsa_c |

| | |
|---|---|
| irc_0006152 | irc_0081245 |
| hsa_circ_0018299 | hsa_circ_0081247 |
| hsa_circ_0007389 | hsa_circ_0081248 |
| hsa_circ_0002342 | hsa_circ_0081249 |
| hsa_circ_0018419 | hsa_circ_0081355 |
| hsa_circ_0018420 | hsa_circ_0081356 |
| hsa_circ_0018422 | hsa_circ_0081358 |
| hsa_circ_0018426 | hsa_circ_0081371 |
| hsa_circ_0018428 | hsa_circ_0081462 |
| hsa_circ_0018429 | hsa_circ_0081463 |
| hsa_circ_0018431 | hsa_circ_0081479 |
| hsa_circ_0018435 | hsa_circ_0081538 |
| hsa_circ_0018436 | hsa_circ_0081539 |
| hsa_circ_0018458 | hsa_circ_0081540 |

| | |
|---|---|
| hsa_circ_0018465 | hsa_circ_0081541 |
| hsa_circ_0008523 | hsa_circ_0081611 |
| hsa_circ_0018564 | sa_circ_0081633 |
| hsa_circ_0018565 | hsa_circ_0081675 |
| hsa_circ_0018566 | hsa_circ_0081676 |
| hsa_circ_0018567 | hsa_circ_0081677 |
| hsa_circ_0018568 | hsa_circ_0081678 |
| hsa_circ_0018569 | hsa_circ_0081878 |
| hsa_circ_0018570 | hsa_circ_0081879 |
| hsa_circ_0018571 | hsa_circ_0003932 |
| hsa_circ_0018572 | hsa_circ_0081907 |
| hsa_circ_0018573 | hsa_circ_0081917 |
| hsa_circ_0018574 | hsa_circ_0081918 |
| hsa_circ_0187 | hsa_circ_0819 |

| | |
|---|---|
| 52 | 25 |
| hsa_circ_0018816 | hsa_circ_0081926 |
| hsa_circ_0018817 | hsa_circ_0081929 |
| hsa_circ_0018819 | hsa_circ_0081932 |
| hsa_circ_0018820 | hsa_circ_0081934 |
| hsa_circ_0008374 | hsa_circ_0081935 |
| hsa_circ_0018940 | hsa_circ_0081937 |
| hsa_circ_0018962 | hsa_circ_0081968 |
| hsa_circ_0018964 | hsa_circ_0081969 |
| hsa_circ_0019038 | hsa_circ_0081970 |
| hsa_circ_0019039 | hsa_circ_0081972 |
| hsa_circ_0019042 | hsa_circ_0081974 |
| hsa_circ_0019118 | hsa_circ_0081975 |
| hsa_circ_0019181 | hsa_circ_0081978 |
| hsa_c | hsa_c |

| | |
|---|---|
| irc_0019183 | irc_0082016 |
| hsa_circ_0019184 | hsa_circ_0006252 |
| hsa_circ_0019186 | hsa_circ_0082168 |
| hsa_circ_0019187 | hsa_circ_0082169 |
| hsa_circ_0019188 | hsa_circ_0082185 |
| hsa_circ_0019189 | hsa_circ_0082192 |
| hsa_circ_0019191 | hsa_circ_0082199 |
| hsa_circ_0019192 | hsa_circ_0082201 |
| hsa_circ_0019265 | hsa_circ_0082202 |
| hsa_circ_0019266 | hsa_circ_0082208 |
| hsa_circ_0019269 | hsa_circ_0082216 |
| hsa_circ_0019270 | hsa_circ_0082217 |
| hsa_circ_0019271 | hsa_circ_0082220 |
| hsa_circ_0006198 | hsa_circ_0082221 |

| | |
|---|---|
| hsa_circ_0019374 | hsa_circ_0082222 |
| hsa_circ_0019732 | hsa_circ_0082233 |
| hsa_circ_0019733 | hsa_circ_0082234 |
| hsa_circ_0019769 | hsa_circ_0082235 |
| hsa_circ_0019771 | hsa_circ_0082339 |
| hsa_circ_0019772 | hsa_circ_0082342 |
| hsa_circ_0019778 | hsa_circ_0082346 |
| hsa_circ_0019780 | hsa_circ_0082351 |
| hsa_circ_0019782 | hsa_circ_0082352 |
| hsa_circ_0019783 | hsa_circ_0082353 |
| hsa_circ_0019785 | hsa_circ_0082354 |
| hsa_circ_0019786 | hsa_circ_0082355 |
| hsa_circ_0019975 | hsa_circ_0082356 |
| hsa_circ_00200 | hsa_circ_00823 |

| | |
|---|---|
| 13 | 57 |
| hsa_circ_0020014 | hsa_circ_0082369 |
| hsa_circ_0020046 | hsa_circ_0082371 |
| hsa_circ_0020119 | hsa_circ_0082372 |
| hsa_circ_0020120 | hsa_circ_0082373 |
| hsa_circ_0020257 | hsa_circ_0082374 |
| hsa_circ_0020258 | hsa_circ_0082375 |
| hsa_circ_0020259 | hsa_circ_0082376 |
| hsa_circ_0003462 | hsa_circ_0082561 |
| hsa_circ_0020486 | hsa_circ_0082562 |
| hsa_circ_0020487 | hsa_circ_0082570 |
| hsa_circ_0020488 | hsa_circ_0082611 |
| hsa_circ_0020555 | hsa_circ_0082618 |
| hsa_circ_0020556 | hsa_circ_0082619 |
| hsa_c | hsa_c |

| | |
|---|---|
| irc_0020559 | irc_0082621 |
| hsa_circ_0020669 | hsa_circ_0082639 |
| hsa_circ_0020731 | hsa_circ_0082642 |
| hsa_circ_0020732 | hsa_circ_0082643 |
| hsa_circ_0021205 | hsa_circ_0082644 |
| hsa_circ_0021295 | hsa_circ_0082645 |
| hsa_circ_0021343 | hsa_circ_0082646 |
| hsa_circ_0021354 | hsa_circ_0082651 |
| hsa_circ_0021355 | hsa_circ_0082652 |
| hsa_circ_0021356 | hsa_circ_0082653 |
| hsa_circ_0021357 | hsa_circ_0082654 |
| hsa_circ_0021358 | hsa_circ_0082660 |
| hsa_circ_0021359 | hsa_circ_0082791 |
| hsa_circ_0021360 | hsa_circ_0082824 |

| | |
|---|---|
| hsa_circ_0021361 | hsa_circ_0082825 |
| hsa_circ_0021362 | hsa_circ_0082826 |
| hsa_circ_0021363 | hsa_circ_0004930 |
| hsa_circ_0021364 | hsa_circ_0082866 |
| hsa_circ_0021365 | hsa_circ_0082868 |
| hsa_circ_0021578 | hsa_circ_0002659 |
| hsa_circ_0021580 | hsa_circ_0082916 |
| hsa_circ_0021581 | hsa_circ_0082957 |
| hsa_circ_0021629 | hsa_circ_0082959 |
| hsa_circ_0021649 | hsa_circ_0082960 |
| hsa_circ_0021716 | hsa_circ_0082961 |
| hsa_circ_0021723 | hsa_circ_0082963 |
| hsa_circ_0021732 | hsa_circ_0082964 |
| hsa_circ_00217 | hsa_circ_00829 |

| | |
|---|---|
| 33 | 66 |
| hsa_circ_0021736 | hsa_circ_0082967 |
| hsa_circ_0021740 | hsa_circ_0082968 |
| hsa_circ_0021741 | hsa_circ_0082970 |
| hsa_circ_0021742 | hsa_circ_0083120 |
| hsa_circ_0021743 | hsa_circ_0083133 |
| hsa_circ_0021765 | hsa_circ_0083137 |
| hsa_circ_0021766 | hsa_circ_0083298 |
| hsa_circ_0021768 | hsa_circ_0083432 |
| hsa_circ_0021776 | hsa_circ_0083433 |
| hsa_circ_0021822 | hsa_circ_0083434 |
| hsa_circ_0021823 | hsa_circ_0016100 |
| hsa_circ_0021837 | hsa_circ_0083596 |
| hsa_circ_0021838 | hsa_circ_0083607 |
| hsa_c | hsa_c |

| | |
|---|---|
| irc_0021958 | irc_0083608 |
| hsa_circ_0022129 | hsa_circ_0083611 |
| hsa_circ_0022131 | hsa_circ_0083612 |
| hsa_circ_0022134 | hsa_circ_0083614 |
| hsa_circ_0022135 | hsa_circ_0002081 |
| hsa_circ_0022156 | hsa_circ_0006320 |
| hsa_circ_0022157 | hsa_circ_0083619 |
| hsa_circ_0022158 | hsa_circ_0083620 |
| hsa_circ_0022426 | hsa_circ_0083621 |
| hsa_circ_0022427 | hsa_circ_0083622 |
| hsa_circ_0022428 | hsa_circ_0083623 |
| hsa_circ_0022429 | hsa_circ_0083624 |
| hsa_circ_0022454 | hsa_circ_0083647 |
| hsa_circ_0022457 | hsa_circ_0083650 |

| | |
|---|---|
| hsa_circ_0022458 | hsa_circ_0083651 |
| hsa_circ_0022459 | hsa_circ_0083728 |
| hsa_circ_0022461 | hsa_circ_0083771 |
| hsa_circ_0022464 | hsa_circ_0083773 |
| hsa_circ_0003843 | hsa_circ_0083774 |
| hsa_circ_0022601 | hsa_circ_0083776 |
| hsa_circ_0003812 | hsa_circ_0083778 |
| hsa_circ_0002791 | hsa_circ_0007800 |
| hsa_circ_0006417 | hsa_circ_0083848 |
| hsa_circ_0007185 | hsa_circ_0083852 |
| hsa_circ_0005929 | hsa_circ_0083853 |
| hsa_circ_0005915 | hsa_circ_0083903 |
| hsa_circ_0022832 | hsa_circ_0083904 |
| hsa_circ_0228 | hsa_circ_0839 |

| | |
|---|---|
| 62 | 05 |
| hsa_circ_0022863 | hsa_circ_0083942 |
| hsa_circ_0022864 | hsa_circ_0083979 |
| hsa_circ_0022865 | hsa_circ_0083980 |
| hsa_circ_0022866 | hsa_circ_0083981 |
| hsa_circ_0022867 | hsa_circ_0083982 |
| hsa_circ_0022926 | hsa_circ_0084064 |
| hsa_circ_0002440 | hsa_circ_0084073 |
| hsa_circ_0023012 | hsa_circ_0084075 |
| hsa_circ_0023014 | hsa_circ_0084178 |
| hsa_circ_0023187 | hsa_circ_0084179 |
| hsa_circ_0023247 | hsa_circ_0008481 |
| hsa_circ_0023251 | hsa_circ_001449 |
| hsa_circ_0023252 | hsa_circ_0084496 |
| hsa_c | hsa_c |

| | |
|---|---|
| irc_0023254 | irc_0084525 |
| hsa_circ_0023257 | hsa_circ_0084526 |
| hsa_circ_0023258 | hsa_circ_0084587 |
| hsa_circ_0023259 | hsa_circ_0084588 |
| hsa_circ_0023335 | hsa_circ_0084589 |
| hsa_circ_0023367 | hsa_circ_0084590 |
| hsa_circ_0023373 | hsa_circ_0084591 |
| hsa_circ_0023375 | hsa_circ_0084593 |
| hsa_circ_0023376 | hsa_circ_0084594 |
| hsa_circ_0023377 | hsa_circ_0084623 |
| hsa_circ_0023414 | hsa_circ_0084650 |
| hsa_circ_0023415 | hsa_circ_0084651 |
| hsa_circ_0023416 | hsa_circ_0084652 |
| hsa_circ_0023474 | hsa_circ_0084778 |

| | |
|---|---|
| hsa_circ_0023476 | hsa_circ_0084791 |
| hsa_circ_001748 | hsa_circ_0084793 |
| hsa_circ_0023531 | hsa_circ_0084794 |
| hsa_circ_0023840 | hsa_circ_0084803 |
| hsa_circ_0023841 | hsa_circ_0084928 |
| hsa_circ_0023842 | hsa_circ_0008608 |
| hsa_circ_0023850 | hsa_circ_0084955 |
| hsa_circ_0023851 | hsa_circ_0085002 |
| hsa_circ_0023853 | hsa_circ_0085004 |
| hsa_circ_0023854 | hsa_circ_0085005 |
| hsa_circ_0023857 | hsa_circ_0085007 |
| hsa_circ_0023860 | hsa_circ_0085008 |
| hsa_circ_0023866 | hsa_circ_0005934 |
| hsa_circ_00238 | hsa_circ_00851 |

| | |
|---|---|
| 67 | 71 |
| hsa_circ_0023868 | hsa_circ_0085174 |
| hsa_circ_0023869 | hsa_circ_0085175 |
| hsa_circ_0023870 | hsa_circ_0085288 |
| hsa_circ_0023880 | hsa_circ_0085289 |
| hsa_circ_0009016 | hsa_circ_0085290 |
| hsa_circ_0024055 | hsa_circ_0085291 |
| hsa_circ_0024056 | hsa_circ_0085386 |
| hsa_circ_0024060 | hsa_circ_0002663 |
| hsa_circ_0024069 | hsa_circ_0007779 |
| hsa_circ_0024070 | hsa_circ_0085452 |
| hsa_circ_0024071 | hsa_circ_0002788 |
| hsa_circ_0024072 | hsa_circ_0085483 |
| hsa_circ_0024073 | hsa_circ_0085484 |
| hsa_c | hsa_c |

| | |
|---|---|
| irc_0 0243 10 | irc_0 0854 88 |
| hsa_c irc_0 0243 11 | hsa_c irc_0 0854 89 |
| hsa_c irc_0 0243 12 | hsa_c irc_0 0854 90 |
| hsa_c irc_0 0244 06 | hsa_c irc_0 0854 91 |
| hsa_c irc_0 0244 07 | hsa_c irc_0 0855 22 |
| hsa_c irc_0 0244 08 | hsa_c irc_0 0855 27 |
| hsa_c irc_0 0244 09 | hsa_c irc_0 0855 28 |
| hsa_c irc_0 0244 10 | hsa_c irc_0 0855 30 |
| hsa_c irc_0 0244 41 | hsa_c irc_0 0855 31 |
| hsa_c irc_0 0244 50 | hsa_c irc_0 0855 32 |
| hsa_c irc_0 0244 51 | hsa_c irc_0 0081 47 |
| hsa_c irc_0 0244 52 | hsa_c irc_0 0855 60 |
| hsa_c irc_0 0244 58 | hsa_c irc_0 0045 15 |
| hsa_c irc_0 0244 59 | hsa_c irc_0 0053 90 |

| | |
|---|---|
| hsa_circ_0024462 | hsa_circ_0085641 |
| hsa_circ_0024475 | hsa_circ_0085677 |
| hsa_circ_0024563 | hsa_circ_0085678 |
| hsa_circ_0024564 | hsa_circ_0085679 |
| hsa_circ_0024565 | hsa_circ_0085680 |
| hsa_circ_0024566 | hsa_circ_0085791 |
| hsa_circ_0024567 | hsa_circ_0085836 |
| hsa_circ_0024568 | hsa_circ_0085897 |
| hsa_circ_0024569 | hsa_circ_0085898 |
| hsa_circ_0024570 | hsa_circ_0086053 |
| hsa_circ_001455 | hsa_circ_0086054 |
| hsa_circ_0024609 | hsa_circ_0086055 |
| hsa_circ_0024610 | hsa_circ_0086056 |
| hsa_circ_00246 | hsa_circ_00860 |

| | |
|---|---|
| 11 | 57 |
| hsa_circ_024612 | hsa_circ_086058 |
| hsa_circ_024613 | hsa_circ_086059 |
| hsa_circ_024614 | hsa_circ_086060 |
| hsa_circ_024615 | hsa_circ_086061 |
| hsa_circ_024618 | hsa_circ_086062 |
| hsa_circ_024620 | hsa_circ_086152 |
| hsa_circ_024802 | hsa_circ_086243 |
| hsa_circ_024803 | hsa_circ_086244 |
| hsa_circ_024804 | hsa_circ_005890 |
| hsa_circ_024805 | hsa_circ_086285 |
| hsa_circ_024806 | hsa_circ_086307 |
| hsa_circ_024993 | hsa_circ_086334 |
| hsa_circ_025018 | hsa_circ_007170 |
| hsa_c | hsa_c |

| | |
|---|---|
| irc_0025023 | irc_0008273 |
| hsa_circ_0025049 | hsa_circ_0086452 |
| hsa_circ_0025055 | hsa_circ_0086453 |
| hsa_circ_0025057 | hsa_circ_0086454 |
| hsa_circ_0025059 | hsa_circ_0086584 |
| hsa_circ_0007377 | hsa_circ_0086586 |
| hsa_circ_0006957 | hsa_circ_0003306 |
| hsa_circ_0008221 | hsa_circ_0086765 |
| hsa_circ_0009010 | hsa_circ_0086766 |
| hsa_circ_00554 | hsa_circ_0086770 |
| hsa_circ_0025468 | hsa_circ_0005833 |
| hsa_circ_0025502 | hsa_circ_0087105 |
| hsa_circ_0025545 | hsa_circ_0087185 |
| hsa_circ_0025546 | hsa_circ_0087186 |

| | |
|---|---|
| hsa_circ_0025547 | hsa_circ_0087187 |
| hsa_circ_0025548 | hsa_circ_0087198 |
| hsa_circ_0025549 | hsa_circ_0087200 |
| hsa_circ_0025550 | hsa_circ_0087204 |
| hsa_circ_0004149 | hsa_circ_0087217 |
| hsa_circ_0005824 | hsa_circ_0087330 |
| hsa_circ_0007478 | hsa_circ_0087331 |
| hsa_circ_0025699 | hsa_circ_0087332 |
| hsa_circ_0025700 | hsa_circ_0087333 |
| hsa_circ_0025705 | hsa_circ_0005432 |
| hsa_circ_0025706 | hsa_circ_0005433 |
| hsa_circ_0025707 | hsa_circ_00770 |
| hsa_circ_0025708 | hsa_circ_0087469 |
| hsa_circ_00257 | hsa_circ_00874 |

| | |
|---|---|
| 09 | 70 |
| hsa_circ_0005234 | hsa_circ_0087471 |
| hsa_circ_0025738 | hsa_circ_0087472 |
| hsa_circ_0025824 | hsa_circ_0087473 |
| hsa_circ_0025926 | hsa_circ_0087474 |
| hsa_circ_0025927 | hsa_circ_0087475 |
| hsa_circ_0025928 | hsa_circ_0087476 |
| hsa_circ_0025952 | hsa_circ_0087477 |
| hsa_circ_0025953 | hsa_circ_0087478 |
| hsa_circ_0025954 | hsa_circ_0087479 |
| hsa_circ_0025955 | hsa_circ_0087480 |
| hsa_circ_0025957 | hsa_circ_0008720 |
| hsa_circ_0025958 | hsa_circ_0004605 |
| hsa_circ_0025975 | hsa_circ_0087613 |
| hsa_c | hsa_c |

| | |
|---|---|
| irc_0003360 | irc_0087650 |
| hsa_circ_0026057 | hsa_circ_0087665 |
| hsa_circ_0026070 | hsa_circ_0087666 |
| hsa_circ_0026071 | hsa_circ_0087748 |
| hsa_circ_0026072 | hsa_circ_0087788 |
| hsa_circ_0026073 | hsa_circ_0087789 |
| hsa_circ_0026074 | hsa_circ_0087790 |
| hsa_circ_0026148 | hsa_circ_0087791 |
| hsa_circ_0026298 | hsa_circ_0087792 |
| hsa_circ_0026299 | hsa_circ_0087832 |
| hsa_circ_0026314 | hsa_circ_0087834 |
| hsa_circ_0026624 | hsa_circ_0087844 |
| hsa_circ_0026635 | hsa_circ_0087845 |
| hsa_circ_0026672 | hsa_circ_0087849 |

| | |
|---|---|
| hsa_circ_0026671 | hsa_circ_0087864 |
| hsa_circ_0026989 | hsa_circ_0087948 |
| hsa_circ_0026990 | hsa_circ_0088104 |
| hsa_circ_0027246 | hsa_circ_0088127 |
| hsa_circ_0027341 | hsa_circ_0088130 |
| hsa_circ_0027342 | hsa_circ_0088131 |
| hsa_circ_0027344 | hsa_circ_0088143 |
| hsa_circ_0027345 | hsa_circ_0088167 |
| hsa_circ_0027350 | hsa_circ_002004 |
| hsa_circ_0027360 | hsa_circ_0088191 |
| hsa_circ_0027361 | hsa_circ_0088192 |
| hsa_circ_0027404 | hsa_circ_0088196 |
| hsa_circ_0027407 | hsa_circ_0088197 |
| hsa_circ_00274 | hsa_circ_00882 |

| | |
|---|---|
| 08 | 00 |
| hsa_circ_0027409 | hsa_circ_0005456 |
| hsa_circ_0027410 | hsa_circ_0088208 |
| hsa_circ_0027411 | hsa_circ_0088209 |
| hsa_circ_0027412 | hsa_circ_0088210 |
| hsa_circ_0027413 | hsa_circ_0088211 |
| hsa_circ_0027414 | hsa_circ_0088212 |
| hsa_circ_0027415 | hsa_circ_0088262 |
| hsa_circ_0027416 | hsa_circ_0088263 |
| hsa_circ_0027417 | hsa_circ_0088266 |
| hsa_circ_0027418 | hsa_circ_0088304 |
| hsa_circ_0027419 | hsa_circ_0088303 |
| hsa_circ_0027453 | hsa_circ_0088315 |
| hsa_circ_0027454 | hsa_circ_0088354 |
| hsa_c | hsa_c |

| | |
|---|---|
| irc_0027455 | irc_0088353 |
| hsa_circ_0027456 | hsa_circ_0088371 |
| hsa_circ_0027457 | hsa_circ_0088372 |
| hsa_circ_0027517 | hsa_circ_0088373 |
| hsa_circ_0027572 | hsa_circ_0088376 |
| hsa_circ_0027573 | hsa_circ_0088378 |
| hsa_circ_0027599 | hsa_circ_0088379 |
| hsa_circ_0027600 | hsa_circ_0088380 |
| hsa_circ_0027679 | hsa_circ_0088381 |
| hsa_circ_0027689 | hsa_circ_0088382 |
| hsa_circ_0027690 | hsa_circ_0088383 |
| hsa_circ_0027693 | hsa_circ_0088430 |
| hsa_circ_0027694 | hsa_circ_0088434 |
| hsa_circ_0027697 | hsa_circ_0088436 |

| | |
|---|---|
| hsa_circ_002769 6 | hsa_circ_008843 7 |
| hsa_circ_002771 7 | hsa_circ_008843 8 |
| hsa_circ_002771 8 | hsa_circ_008843 9 |
| hsa_circ_000719 9 | hsa_circ_008844 0 |
| hsa_circ_000720 0 | hsa_circ_008844 4 |
| hsa_circ_000720 1 | hsa_circ_008844 2 |
| hsa_circ_002779 3 | hsa_circ_008844 3 |
| hsa_circ_002779 4 | hsa_circ_008850 6 |
| hsa_circ_002779 5 | hsa_circ_008856 6 |
| hsa_circ_002792 6 | hsa_circ_008856 9 |
| hsa_circ_002793 3 | hsa_circ_008857 0 |
| hsa_circ_002793 4 | hsa_circ_008857 1 |
| hsa_circ_002793 7 | hsa_circ_008857 2 |
| hsa_circ_00279 | hsa_circ_00885 |

| | |
|---|---|
| 38 | 73 |
| hsa_circ_0027939 | hsa_circ_0088574 |
| hsa_circ_0027940 | hsa_circ_0088600 |
| hsa_circ_0027941 | hsa_circ_0088601 |
| hsa_circ_0028062 | hsa_circ_0088595 |
| hsa_circ_0028063 | hsa_circ_0088704 |
| hsa_circ_0028064 | hsa_circ_0088736 |
| hsa_circ_0028302 | hsa_circ_0088791 |
| hsa_circ_0028303 | hsa_circ_0088789 |
| hsa_circ_0028304 | hsa_circ_0088902 |
| hsa_circ_0028327 | hsa_circ_0088919 |
| hsa_circ_0028328 | hsa_circ_0088911 |
| hsa_circ_0028354 | hsa_circ_0089043 |
| hsa_circ_0028355 | hsa_circ_0002543 |
| hsa_c | hsa_c |

| | |
|---|---|
| irc_0028356 | irc_0089078 |
| hsa_circ_0028357 | hsa_circ_0089080 |
| hsa_circ_0028358 | hsa_circ_0089081 |
| hsa_circ_0028359 | hsa_circ_0089082 |
| hsa_circ_0028360 | hsa_circ_0089092 |
| hsa_circ_0028361 | hsa_circ_0089093 |
| hsa_circ_0028362 | hsa_circ_0089095 |
| hsa_circ_0028363 | hsa_circ_0089096 |
| hsa_circ_0028364 | hsa_circ_0089097 |
| hsa_circ_0028365 | hsa_circ_0089098 |
| hsa_circ_0028366 | hsa_circ_0089099 |
| hsa_circ_0028367 | hsa_circ_0089100 |
| hsa_circ_0028368 | hsa_circ_0089101 |
| hsa_circ_0028369 | hsa_circ_0089102 |

| | |
|---|---|
| hsa_circ_0028370 | hsa_circ_0089145 |
| hsa_circ_0028371 | hsa_circ_0089146 |
| hsa_circ_0028372 | hsa_circ_0089148 |
| hsa_circ_0028373 | hsa_circ_0089149 |
| hsa_circ_0028448 | hsa_circ_0089151 |
| hsa_circ_0028589 | hsa_circ_0089143 |
| hsa_circ_0028590 | hsa_circ_0089193 |
| hsa_circ_0028881 | hsa_circ_0089192 |
| hsa_circ_0028923 | hsa_circ_0089251 |
| hsa_circ_0028924 | hsa_circ_0089305 |
| hsa_circ_0028925 | hsa_circ_0089306 |
| hsa_circ_0029174 | hsa_circ_0089307 |
| hsa_circ_0029177 | hsa_circ_0089373 |
| hsa_circ_00291 | hsa_circ_00895 |

| | |
|---|---|
| 79 | 17 |
| hsa_circ_0029180 | hsa_circ_0089528 |
| hsa_circ_0029182 | hsa_circ_0089529 |
| hsa_circ_0029183 | hsa_circ_0089530 |
| hsa_circ_0029185 | hsa_circ_0089531 |
| hsa_circ_0029188 | hsa_circ_0089535 |
| hsa_circ_0029189 | hsa_circ_0089539 |
| hsa_circ_0029190 | hsa_circ_0089542 |
| hsa_circ_0029191 | hsa_circ_0089543 |
| hsa_circ_0029192 | hsa_circ_0089544 |
| hsa_circ_0029193 | hsa_circ_0089547 |
| hsa_circ_0029194 | hsa_circ_0089548 |
| hsa_circ_0029195 | hsa_circ_0089552 |
| hsa_circ_0029244 | hsa_circ_0089649 |
| hsa_c | hsa_c |

| | |
|---|---|
| irc_0029245 | irc_0089655 |
| hsa_circ_0029290 | hsa_circ_0089654 |
| hsa_circ_0029291 | hsa_circ_0089694 |
| hsa_circ_0029292 | hsa_circ_0089695 |
| hsa_circ_0029294 | hsa_circ_0002647 |
| hsa_circ_0029295 | hsa_circ_0004101 |
| hsa_circ_0029296 | hsa_circ_0007462 |
| hsa_circ_0029297 | hsa_circ_0089706 |
| hsa_circ_0029298 | hsa_circ_0089707 |
| hsa_circ_0029483 | hsa_circ_0089708 |
| hsa_circ_0029542 | hsa_circ_0089709 |
| hsa_circ_0029555 | hsa_circ_0089710 |
| hsa_circ_0029557 | hsa_circ_0089711 |
| hsa_circ_0029558 | hsa_circ_0089712 |

| | |
|---|---|
| hsa_circ_0029559 | hsa_circ_0017473 |
| hsa_circ_0029560 | hsa_circ_0017474 |
| hsa_circ_0029582 | hsa_circ_0017475 |
| hsa_circ_0029583 | hsa_circ_0017476 |
| hsa_circ_0029584 | hsa_circ_0017477 |
| hsa_circ_0029603 | hsa_circ_0017478 |
| hsa_circ_0006481 | hsa_circ_0017785 |
| hsa_circ_0006833 | hsa_circ_0017603 |
| hsa_circ_0029668 | hsa_circ_0017606 |
| hsa_circ_0029675 | hsa_circ_0017612 |
| hsa_circ_0029679 | hsa_circ_0017613 |
| hsa_circ_0029680 | hsa_circ_0017614 |
| hsa_circ_0029684 | hsa_circ_0017615 |
| hsa_circ_00296 | hsa_circ_00176 |

| | |
|---|---|
| 98 | 16 |
| hsa_circ_0029699 | hsa_circ_0017617 |
| hsa_circ_0029700 | hsa_circ_0017618 |
| hsa_circ_0029701 | hsa_circ_0017619 |
| hsa_circ_0029715 | hsa_circ_0017620 |
| hsa_circ_0029734 | hsa_circ_0003978 |
| hsa_circ_0029908 | hsa_circ_0006664 |
| hsa_circ_0030021 | hsa_circ_0004201 |
| hsa_circ_0030142 | hsa_circ_0005697 |
| hsa_circ_0030143 | hsa_circ_0017827 |
| hsa_circ_0030204 | hsa_circ_0017983 |
| hsa_circ_0030205 | hsa_circ_0017984 |
| hsa_circ_0030208 | hsa_circ_0017985 |
| hsa_circ_0030210 | hsa_circ_0017986 |
| hsa_c | hsa_c |

| | |
|---|---|
| irc_0030211 | irc_0017987 |
| hsa_circ_0030212 | hsa_circ_0018002 |
| hsa_circ_0030215 | hsa_circ_0018007 |
| hsa_circ_0030216 | hsa_circ_0005125 |
| hsa_circ_0030217 | hsa_circ_0018024 |
| hsa_circ_0030234 | hsa_circ_0018025 |
| hsa_circ_0030258 | hsa_circ_0018026 |
| hsa_circ_0030325 | hsa_circ_0018067 |
| hsa_circ_0030554 | hsa_circ_0004126 |
| hsa_circ_0030555 | hsa_circ_0008203 |
| hsa_circ_0030566 | hsa_circ_0018087 |
| hsa_circ_0030657 | hsa_circ_001552 |
| hsa_circ_0030658 | hsa_circ_00782 |
| hsa_circ_0030659 | hsa_circ_0018178 |

| | |
|---|---|
| hsa_circ_0030660 | hsa_circ_0018209 |
| hsa_circ_0030661 | hsa_circ_0018246 |
| hsa_circ_0030662 | hsa_circ_0018280 |
| hsa_circ_0030709 | hsa_circ_0002461 |
| hsa_circ_0030712 | hsa_circ_0003547 |
| hsa_circ_0031149 | hsa_circ_0008028 |
| hsa_circ_0031152 | hsa_circ_0005911 |
| hsa_circ_0031153 | hsa_circ_0006152 |
| hsa_circ_0031170 | hsa_circ_0004097 |
| hsa_circ_0031171 | hsa_circ_0005048 |
| hsa_circ_0031172 | hsa_circ_0004986 |
| hsa_circ_0031173 | hsa_circ_0018299 |
| hsa_circ_0031174 | hsa_circ_0007389 |
| hsa_circ_00311 | hsa_circ_00023 |

| | |
|---|---|
| 75 | 42 |
| hsa_circ_0031176 | hsa_circ_0008931 |
| hsa_circ_0031180 | hsa_circ_0003083 |
| hsa_circ_0031181 | hsa_circ_0002010 |
| hsa_circ_0031182 | hsa_circ_0018334 |
| hsa_circ_0031183 | hsa_circ_0018335 |
| hsa_circ_0031403 | hsa_circ_0018336 |
| hsa_circ_0031404 | hsa_circ_0018337 |
| hsa_circ_0031500 | hsa_circ_0018338 |
| hsa_circ_0031501 | hsa_circ_0018339 |
| hsa_circ_0031502 | hsa_circ_0018364 |
| hsa_circ_0031503 | hsa_circ_0018368 |
| hsa_circ_0031504 | hsa_circ_0018369 |
| hsa_circ_0031505 | hsa_circ_0018371 |
| hsa_c | hsa_c |

| | |
|---|---|
| irc_0031506 | irc_0005448 |
| hsa_circ_0031578 | hsa_circ_0003833 |
| hsa_circ_0031751 | hsa_circ_0018409 |
| hsa_circ_0031752 | hsa_circ_0018420 |
| hsa_circ_0031753 | hsa_circ_0018419 |
| hsa_circ_0002819 | hsa_circ_0018436 |
| hsa_circ_0003616 | hsa_circ_0018435 |
| hsa_circ_0003689 | hsa_circ_0018444 |
| hsa_circ_0005215 | hsa_circ_0018467 |
| hsa_circ_0006066 | hsa_circ_0018507 |
| hsa_circ_0008088 | hsa_circ_0018540 |
| hsa_circ_0008845 | hsa_circ_0018537 |
| hsa_circ_0009080 | hsa_circ_0018653 |
| hsa_circ_0031772 | hsa_circ_0018655 |

| | |
|---|---|
| hsa_circ_0031816 | hsa_circ_0018662 |
| hsa_circ_0002949 | hsa_circ_0018663 |
| hsa_circ_0031856 | hsa_circ_0018660 |
| hsa_circ_0031857 | hsa_circ_0018696 |
| hsa_circ_0031858 | hsa_circ_0018697 |
| hsa_circ_0031859 | hsa_circ_0018698 |
| hsa_circ_0031860 | hsa_circ_0018699 |
| hsa_circ_0031861 | hsa_circ_0018706 |
| hsa_circ_0031876 | hsa_circ_0018707 |
| hsa_circ_0004742 | hsa_circ_0018716 |
| hsa_circ_0008176 | hsa_circ_0018717 |
| hsa_circ_0031905 | hsa_circ_0018720 |
| hsa_circ_0031906 | hsa_circ_0018721 |
| hsa_circ_00319 | hsa_circ_00187 |

| | |
|---|---|
| 11 | 29 |
| hsa_circ_0031912 | hsa_circ_0018755 |
| hsa_circ_0031914 | hsa_circ_0018756 |
| hsa_circ_0031915 | hsa_circ_0018757 |
| hsa_circ_0031966 | hsa_circ_0018758 |
| hsa_circ_0031973 | hsa_circ_001747 |
| hsa_circ_0031986 | hsa_circ_000094 |
| hsa_circ_0002612 | hsa_circ_0018866 |
| hsa_circ_0031989 | hsa_circ_0018874 |
| hsa_circ_0031990 | hsa_circ_001838 |
| hsa_circ_0031991 | hsa_circ_0018941 |
| hsa_circ_0031992 | hsa_circ_0018942 |
| hsa_circ_0032024 | hsa_circ_0019002 |
| hsa_circ_0032026 | hsa_circ_0019003 |
| hsa_c | hsa_c |

| | |
|---|---|
| irc_0032034 | irc_0019004 |
| hsa_circ_0032067 | hsa_circ_0019005 |
| hsa_circ_0032068 | hsa_circ_0019006 |
| hsa_circ_0032069 | hsa_circ_0019007 |
| hsa_circ_0032070 | hsa_circ_0019008 |
| hsa_circ_0032071 | hsa_circ_0019009 |
| hsa_circ_0032072 | hsa_circ_0019038 |
| hsa_circ_0032112 | hsa_circ_0019039 |
| hsa_circ_0032113 | hsa_circ_0019042 |
| hsa_circ_0032124 | hsa_circ_0019045 |
| hsa_circ_0032157 | hsa_circ_0008629 |
| hsa_circ_0032158 | hsa_circ_0019064 |
| hsa_circ_0032215 | hsa_circ_0019065 |
| hsa_circ_0032217 | hsa_circ_0019069 |

| | |
|---|---|
| hsa_circ_0032219 | hsa_circ_0019152 |
| hsa_circ_0032220 | hsa_circ_0019153 |
| hsa_circ_0032221 | hsa_circ_0019154 |
| hsa_circ_0032223 | hsa_circ_0019155 |
| hsa_circ_0032224 | hsa_circ_0019224 |
| hsa_circ_0032225 | hsa_circ_0019227 |
| hsa_circ_0032226 | hsa_circ_0019228 |
| hsa_circ_0032262 | hsa_circ_0019235 |
| hsa_circ_0032311 | hsa_circ_0019236 |
| hsa_circ_0032312 | hsa_circ_0019237 |
| hsa_circ_0032343 | hsa_circ_0019238 |
| hsa_circ_0032344 | hsa_circ_0019239 |
| hsa_circ_00594 | hsa_circ_0019240 |
| hsa_circ_0067 | hsa_circ_0192 |

| | |
|---|---|
| 83 | 46 |
| hsa_circ_0006671 | hsa_circ_0019282 |
| hsa_circ_0032558 | hsa_circ_0006198 |
| hsa_circ_0032559 | hsa_circ_0019379 |
| hsa_circ_0032560 | hsa_circ_0019380 |
| hsa_circ_0032561 | hsa_circ_0019418 |
| hsa_circ_0032562 | hsa_circ_0019419 |
| hsa_circ_0032563 | hsa_circ_0019420 |
| hsa_circ_0032564 | hsa_circ_0019430 |
| hsa_circ_0032565 | hsa_circ_0019444 |
| hsa_circ_0032622 | hsa_circ_0019432 |
| hsa_circ_0032623 | hsa_circ_0019433 |
| hsa_circ_0032624 | hsa_circ_0019434 |
| hsa_circ_0010600 | hsa_circ_0019451 |
| hsa_c | hsa_c |

| | |
|---|---|
| irc_0003819 | irc_0019453 |
| hsa_circ_0005212 | hsa_circ_0019457 |
| hsa_circ_0005252 | hsa_circ_0019460 |
| hsa_circ_0005267 | hsa_circ_0019462 |
| hsa_circ_0032734 | hsa_circ_0019463 |
| hsa_circ_0032735 | hsa_circ_0019484 |
| hsa_circ_0032736 | hsa_circ_0019485 |
| hsa_circ_0032737 | hsa_circ_0019487 |
| hsa_circ_0032738 | hsa_circ_0019488 |
| hsa_circ_0032739 | hsa_circ_0019489 |
| hsa_circ_0032740 | hsa_circ_0019490 |
| hsa_circ_0032741 | hsa_circ_0019491 |
| hsa_circ_0032742 | hsa_circ_0019511 |
| hsa_circ_0032755 | hsa_circ_0019513 |

| | |
|---|---|
| hsa_circ_0004187 | hsa_circ_0019514 |
| hsa_circ_0006706 | hsa_circ_0019519 |
| hsa_circ_0007782 | hsa_circ_0019527 |
| hsa_circ_0032787 | hsa_circ_0019542 |
| hsa_circ_0032788 | hsa_circ_0019544 |
| hsa_circ_0032789 | hsa_circ_0019551 |
| hsa_circ_0032790 | hsa_circ_0019575 |
| hsa_circ_0032791 | hsa_circ_0019576 |
| hsa_circ_0032806 | hsa_circ_0019577 |
| hsa_circ_0032807 | hsa_circ_0019578 |
| hsa_circ_0032808 | hsa_circ_0019580 |
| hsa_circ_0032915 | hsa_circ_0019579 |
| hsa_circ_0032941 | hsa_circ_0019600 |
| hsa_circ_00329 | hsa_circ_00196 |

| | |
|---|---|
| 77 | 01 |
| hsa_circ_0033075 | hsa_circ_0019602 |
| hsa_circ_0033076 | hsa_circ_0019603 |
| hsa_circ_0033077 | hsa_circ_0019604 |
| hsa_circ_0033078 | hsa_circ_0019605 |
| hsa_circ_0033079 | hsa_circ_0019606 |
| hsa_circ_0033080 | hsa_circ_0019607 |
| hsa_circ_0033081 | hsa_circ_0002218 |
| hsa_circ_0033565 | hsa_circ_0002523 |
| hsa_circ_0033566 | hsa_circ_0019642 |
| hsa_circ_0033567 | hsa_circ_0019651 |
| hsa_circ_0033568 | hsa_circ_0019653 |
| hsa_circ_0033569 | hsa_circ_0019654 |
| hsa_circ_0033570 | hsa_circ_0019656 |
| hsa_c | hsa_c |

| | |
|---|---|
| irc_0033571 | irc_0019657 |
| hsa_circ_0033572 | hsa_circ_0019658 |
| hsa_circ_0034092 | hsa_circ_0019659 |
| hsa_circ_0034116 | hsa_circ_0005599 |
| hsa_circ_0034118 | hsa_circ_0019661 |
| hsa_circ_0034120 | hsa_circ_0019662 |
| hsa_circ_0034121 | hsa_circ_0019663 |
| hsa_circ_0034122 | hsa_circ_0019664 |
| hsa_circ_0034123 | hsa_circ_0019666 |
| hsa_circ_0034124 | hsa_circ_0019667 |
| hsa_circ_0034125 | hsa_circ_0019668 |
| hsa_circ_0034126 | hsa_circ_0019721 |
| hsa_circ_0034127 | hsa_circ_0019732 |
| hsa_circ_0034128 | hsa_circ_0019733 |

| | |
|---|---|
| hsa_circ_0034129 | hsa_circ_0019769 |
| hsa_circ_0034132 | hsa_circ_0019771 |
| hsa_circ_0034133 | hsa_circ_0019772 |
| hsa_circ_0034130 | hsa_circ_0019776 |
| hsa_circ_0034131 | hsa_circ_0019778 |
| hsa_circ_0034137 | hsa_circ_0019806 |
| hsa_circ_0034135 | hsa_circ_0019987 |
| hsa_circ_0092367 | hsa_circ_0019988 |
| hsa_circ_0034138 | hsa_circ_0019989 |
| hsa_circ_0034139 | hsa_circ_0019990 |
| hsa_circ_001566 | hsa_circ_0019991 |
| hsa_circ_0034140 | hsa_circ_0019992 |
| hsa_circ_0034321 | hsa_circ_0020025 |
| hsa_circ_00343 | hsa_circ_00200 |

| | |
|---|---|
| 22 | 35 |
| hsa_circ_0034338 | hsa_circ_0020037 |
| hsa_circ_0005001 | hsa_circ_0020085 |
| hsa_circ_0034446 | hsa_circ_0020126 |
| hsa_circ_0034461 | hsa_circ_0020127 |
| hsa_circ_0034479 | hsa_circ_0020128 |
| hsa_circ_0034484 | hsa_circ_0020213 |
| hsa_circ_0034494 | hsa_circ_0020215 |
| hsa_circ_0034509 | hsa_circ_0020216 |
| hsa_circ_0034515 | hsa_circ_0020217 |
| hsa_circ_0034526 | hsa_circ_0020219 |
| hsa_circ_0034531 | hsa_circ_0020220 |
| hsa_circ_0034537 | hsa_circ_0020222 |
| hsa_circ_0034539 | hsa_circ_0020224 |
| hsa_c | hsa_c |

| | |
|---|---|
| irc_0034542 | irc_0020225 |
| hsa_circ_0034544 | hsa_circ_0020226 |
| hsa_circ_0034545 | hsa_circ_0020303 |
| hsa_circ_0034548 | hsa_circ_0020304 |
| hsa_circ_0034549 | hsa_circ_0020306 |
| hsa_circ_0034955 | hsa_circ_0020307 |
| hsa_circ_0034956 | hsa_circ_0020308 |
| hsa_circ_0034961 | hsa_circ_0019001 |
| hsa_circ_0034963 | hsa_circ_0020316 |
| hsa_circ_0034964 | hsa_circ_0004451 |
| hsa_circ_0034965 | hsa_circ_0020375 |
| hsa_circ_0034985 | hsa_circ_0020376 |
| hsa_circ_0034990 | hsa_circ_0020570 |
| hsa_circ_0034993 | hsa_circ_0020571 |

| | |
|---|---|
| hsa_circ_0034995 | hsa_circ_0020572 |
| hsa_circ_0034976 | hsa_circ_0020594 |
| hsa_circ_0034996 | hsa_circ_0020595 |
| hsa_circ_0034997 | hsa_circ_0020596 |
| hsa_circ_0034998 | hsa_circ_0020793 |
| hsa_circ_0034999 | hsa_circ_0020808 |
| hsa_circ_0035069 | hsa_circ_0020809 |
| hsa_circ_0035070 | hsa_circ_0020850 |
| hsa_circ_0035071 | hsa_circ_0020851 |
| hsa_circ_0035076 | hsa_circ_0020854 |
| hsa_circ_0035078 | hsa_circ_0020855 |
| hsa_circ_0035079 | hsa_circ_0020856 |
| hsa_circ_0350 | hsa_circ_0208 |

| | |
|---|---|
| 80 | 58 |
| hsa_circ_0035081 | hsa_circ_0020860 |
| hsa_circ_0035082 | hsa_circ_0020861 |
| hsa_circ_0035083 | hsa_circ_0020862 |
| hsa_circ_0035084 | hsa_circ_0020863 |
| hsa_circ_0035341 | hsa_circ_0020921 |
| hsa_circ_0035342 | hsa_circ_0020924 |
| hsa_circ_0035343 | hsa_circ_0020923 |
| hsa_circ_0035344 | hsa_circ_0020970 |
| hsa_circ_0035345 | hsa_circ_0021038 |
| hsa_circ_0035428 | hsa_circ_0021042 |
| hsa_circ_0035430 | hsa_circ_0021043 |
| hsa_circ_0035431 | hsa_circ_0021056 |
| hsa_circ_0035432 | hsa_circ_0021057 |
| hsa_c | hsa_c |

| | |
|---|---|
| irc_0035433 | irc_0021058 |
| hsa_circ_0001982 | hsa_circ_0005944 |
| hsa_circ_0035472 | hsa_circ_0008486 |
| hsa_circ_0035473 | hsa_circ_0021094 |
| hsa_circ_0035474 | hsa_circ_0021095 |
| hsa_circ_0035475 | hsa_circ_0021096 |
| hsa_circ_0035493 | hsa_circ_0021098 |
| hsa_circ_0035494 | hsa_circ_0021100 |
| hsa_circ_0035495 | hsa_circ_0021102 |
| hsa_circ_0035496 | hsa_circ_0021103 |
| hsa_circ_0035497 | hsa_circ_0021104 |
| hsa_circ_0035498 | hsa_circ_0021242 |
| hsa_circ_0035499 | hsa_circ_0021243 |
| hsa_circ_0035500 | hsa_circ_0021245 |

| | |
|---|---|
| hsa_circ_0035501 | hsa_circ_0021246 |
| hsa_circ_00201 1 | hsa_circ_0021247 |
| hsa_circ_0035635 | hsa_circ_0021248 |
| hsa_circ_0035636 | hsa_circ_0021249 |
| hsa_circ_0035637 | hsa_circ_0021250 |
| hsa_circ_0035638 | hsa_circ_0021251 |
| hsa_circ_0035639 | hsa_circ_0021280 |
| hsa_circ_0035824 | hsa_circ_0021328 |
| hsa_circ_0035825 | hsa_circ_0021345 |
| hsa_circ_0035826 | hsa_circ_0021358 |
| hsa_circ_0035856 | hsa_circ_0021359 |
| hsa_circ_0035858 | hsa_circ_0021360 |
| hsa_circ_0036012 | hsa_circ_0021361 |
| hsa_circ_00050 | hsa_circ_00213 |

| | |
|---|---|
| 65 | 62 |
| hsa_circ_0036063 | hsa_circ_0021363 |
| hsa_circ_0036072 | hsa_circ_0021364 |
| hsa_circ_0036082 | hsa_circ_0021365 |
| hsa_circ_0036187 | hsa_circ_0021367 |
| hsa_circ_0036188 | hsa_circ_0021366 |
| hsa_circ_0036189 | hsa_circ_0021479 |
| hsa_circ_0036192 | hsa_circ_0021484 |
| hsa_circ_0036193 | hsa_circ_0021485 |
| hsa_circ_0036195 | hsa_circ_0021487 |
| hsa_circ_0036196 | hsa_circ_0021488 |
| hsa_circ_0036200 | hsa_circ_0021489 |
| hsa_circ_0036204 | hsa_circ_0021518 |
| hsa_circ_0036227 | hsa_circ_0021519 |
| hsa_c | hsa_c |

| | |
|---|---|
| irc_0036233 | irc_0021607 |
| hsa_circ_0036235 | hsa_circ_0021608 |
| hsa_circ_0036236 | hsa_circ_0021610 |
| hsa_circ_0036237 | hsa_circ_0021611 |
| hsa_circ_0036238 | hsa_circ_0021612 |
| hsa_circ_0036245 | hsa_circ_0021754 |
| hsa_circ_0036260 | hsa_circ_0021769 |
| hsa_circ_0036358 | hsa_circ_0021793 |
| hsa_circ_0036421 | hsa_circ_0021822 |
| hsa_circ_0036451 | hsa_circ_0022016 |
| hsa_circ_0036450 | hsa_circ_0022017 |
| hsa_circ_0036600 | hsa_circ_0022021 |
| hsa_circ_0036602 | hsa_circ_0022022 |
| hsa_circ_0036604 | hsa_circ_0022023 |

| | |
|---|---|
| hsa_circ_003667 5 | hsa_circ_002202 6 |
| hsa_circ_003667 8 | hsa_circ_002202 7 |
| hsa_circ_003668 0 | hsa_circ_002202 8 |
| hsa_circ_003668 2 | hsa_circ_002202 9 |
| hsa_circ_003668 3 | hsa_circ_002203 0 |
| hsa_circ_003668 4 | hsa_circ_002207 4 |
| hsa_circ_003668 5 | hsa_circ_002207 9 |
| hsa_circ_003668 6 | hsa_circ_002209 2 |
| hsa_circ_003668 7 | hsa_circ_002209 5 |
| hsa_circ_003676 0 | hsa_circ_002209 6 |
| hsa_circ_003676 1 | hsa_circ_002209 7 |
| hsa_circ_003676 6 | hsa_circ_002210 0 |
| hsa_circ_003676 7 | hsa_circ_002210 2 |
| hsa_circ_0 | hsa_circ_0 |

| | |
|---|---|
| 036768 | 022103 |
| hsa_circ_0036769 | hsa_circ_0022104 |
| hsa_circ_0036770 | hsa_circ_0022129 |
| hsa_circ_0036771 | hsa_circ_0022136 |
| hsa_circ_0036772 | hsa_circ_0022137 |
| hsa_circ_001909 | hsa_circ_0022138 |
| hsa_circ_0036782 | hsa_circ_0022159 |
| hsa_circ_0036783 | hsa_circ_0004988 |
| hsa_circ_0036784 | hsa_circ_0022201 |
| hsa_circ_0036955 | hsa_circ_0022202 |
| hsa_circ_0037024 | hsa_circ_0022342 |
| hsa_circ_0037025 | hsa_circ_0022343 |
| hsa_circ_0037027 | hsa_circ_0022344 |
| hsa_circ_0037028 | hsa_circ_0022345 |

| | |
|---|---|
| hsa_circ_0037032 | hsa_circ_0022346 |
| hsa_circ_0037033 | hsa_circ_0022382 |
| hsa_circ_0037034 | hsa_circ_0022383 |
| hsa_circ_0037035 | hsa_circ_0022384 |
| hsa_circ_0037036 | hsa_circ_0022398 |
| hsa_circ_0037037 | hsa_circ_0022399 |
| hsa_circ_0037046 | hsa_circ_0022400 |
| hsa_circ_0037199 | hsa_circ_0022401 |
| hsa_circ_0037201 | hsa_circ_0022402 |
| hsa_circ_0037328 | hsa_circ_0022403 |
| hsa_circ_0037542 | hsa_circ_0022404 |
| hsa_circ_0037598 | hsa_circ_0022405 |
| hsa_circ_0037621 | hsa_circ_0022470 |
| hsa_circ_00376 | hsa_circ_00225 |

| | |
|---|---|
| 22 | 01 |
| hsa_c<br>irc_0<br>0376<br>23 | hsa_c<br>irc_0<br>0225<br>02 |
| hsa_c<br>irc_0<br>0376<br>24 | hsa_c<br>irc_0<br>0225<br>03 |
| hsa_c<br>irc_0<br>0376<br>25 | hsa_c<br>irc_0<br>0225<br>04 |
| hsa_c<br>irc_0<br>0376<br>26 | hsa_c<br>irc_0<br>0225<br>00 |
| hsa_c<br>irc_0<br>0376<br>27 | hsa_c<br>irc_0<br>0023<br>40 |
| hsa_c<br>irc_0<br>0376<br>28 | hsa_c<br>irc_0<br>0226<br>06 |
| hsa_c<br>irc_0<br>0376<br>29 | hsa_c<br>irc_0<br>0226<br>08 |
| hsa_c<br>irc_0<br>0376<br>30 | hsa_c<br>irc_0<br>0226<br>11 |
| hsa_c<br>irc_0<br>0376<br>31 | hsa_c<br>irc_0<br>0226<br>12 |
| hsa_c<br>irc_0<br>0376<br>32 | hsa_c<br>irc_0<br>0227<br>16 |
| hsa_c<br>irc_0<br>0376<br>33 | hsa_c<br>irc_0<br>0227<br>17 |
| hsa_c<br>irc_0<br>0376<br>34 | hsa_c<br>irc_0<br>0227<br>25 |
| hsa_c<br>irc_0<br>0376<br>35 | hsa_c<br>irc_0<br>0227<br>26 |
| hsa_c | hsa_c |

| | |
|---|---|
| irc_0037636 | irc_0022727 |
| hsa_circ_0037637 | hsa_circ_0022728 |
| hsa_circ_0037638 | hsa_circ_0022729 |
| hsa_circ_0037652 | hsa_circ_0022731 |
| hsa_circ_0037663 | hsa_circ_0022732 |
| hsa_circ_0037664 | hsa_circ_0022733 |
| hsa_circ_0037666 | hsa_circ_0022734 |
| hsa_circ_0037669 | hsa_circ_0022747 |
| hsa_circ_0037670 | hsa_circ_0022752 |
| hsa_circ_0037671 | hsa_circ_0022753 |
| hsa_circ_0037672 | hsa_circ_0022754 |
| hsa_circ_0037673 | hsa_circ_0022782 |
| hsa_circ_0037675 | hsa_circ_0022818 |
| hsa_circ_0037676 | hsa_circ_0022819 |

| | |
|---|---|
| hsa_circ_0037677 | hsa_circ_0022820 |
| hsa_circ_0037694 | hsa_circ_0022821 |
| hsa_circ_0037939 | hsa_circ_0022822 |
| hsa_circ_0037940 | hsa_circ_0022823 |
| hsa_circ_0037943 | hsa_circ_0003812 |
| hsa_circ_0037944 | hsa_circ_0006417 |
| hsa_circ_0037945 | hsa_circ_0007185 |
| hsa_circ_0005155 | hsa_circ_0002791 |
| hsa_circ_0037951 | hsa_circ_0022832 |
| hsa_circ_0037952 | hsa_circ_0022831 |
| hsa_circ_0037953 | hsa_circ_0022859 |
| hsa_circ_0037954 | hsa_circ_0022862 |
| hsa_circ_0037955 | hsa_circ_0022870 |
| hsa_circ_00379 | hsa_circ_00228 |

| | |
|---|---|
| 56 | 79 |
| hsa_circ_0037957 | hsa_circ_0022882 |
| hsa_circ_0037958 | hsa_circ_0022883 |
| hsa_circ_0037959 | hsa_circ_0022884 |
| hsa_circ_0003427 | hsa_circ_0022885 |
| hsa_circ_0003420 | hsa_circ_0022909 |
| hsa_circ_0005409 | hsa_circ_0022913 |
| hsa_circ_0007965 | hsa_circ_0022911 |
| hsa_circ_0038239 | hsa_circ_0022912 |
| hsa_circ_0038262 | hsa_circ_0022934 |
| hsa_circ_0038267 | hsa_circ_0022979 |
| hsa_circ_0038268 | hsa_circ_0022978 |
| hsa_circ_0038271 | hsa_circ_0022988 |
| hsa_circ_0038279 | hsa_circ_0023019 |
| hsa_c | hsa_c |

| | |
|---|---|
| irc_0038286 | irc_0023020 |
| hsa_circ_0038288 | hsa_circ_0023021 |
| hsa_circ_0038290 | hsa_circ_0023022 |
| hsa_circ_0038291 | hsa_circ_0023023 |
| hsa_circ_0038292 | hsa_circ_0023049 |
| hsa_circ_0038373 | hsa_circ_0023066 |
| hsa_circ_0038411 | hsa_circ_0023085 |
| hsa_circ_0038412 | hsa_circ_0023089 |
| hsa_circ_0038414 | hsa_circ_0023090 |
| hsa_circ_0005343 | hsa_circ_0023227 |
| hsa_circ_0038447 | hsa_circ_0023228 |
| hsa_circ_0038448 | hsa_circ_0023251 |
| hsa_circ_0038450 | hsa_circ_0023252 |
| hsa_circ_0038503 | hsa_circ_0023258 |

| | |
|---|---|
| hsa_circ_0038504 | hsa_circ_0023254 |
| hsa_circ_0038505 | hsa_circ_0023262 |
| hsa_circ_0038506 | hsa_circ_0023263 |
| hsa_circ_0038507 | hsa_circ_0023269 |
| hsa_circ_0038508 | hsa_circ_0023268 |
| hsa_circ_0038509 | hsa_circ_0023286 |
| hsa_circ_0038510 | hsa_circ_0023287 |
| hsa_circ_0038532 | hsa_circ_0023288 |
| hsa_circ_0038533 | hsa_circ_0023289 |
| hsa_circ_0038539 | hsa_circ_0023290 |
| hsa_circ_0038546 | hsa_circ_0023291 |
| hsa_circ_0038547 | hsa_circ_0023314 |
| hsa_circ_0038624 | hsa_circ_0023318 |
| hsa_circ_00386 | hsa_circ_00233 |

| | |
|---|---|
| 25 | 16 |
| hsa_circ_038677 | hsa_circ_023320 |
| hsa_circ_038681 | hsa_circ_023321 |
| hsa_circ_038712 | hsa_circ_023418 |
| hsa_circ_038730 | hsa_circ_023419 |
| hsa_circ_038731 | hsa_circ_023428 |
| hsa_circ_038732 | hsa_circ_023431 |
| hsa_circ_038733 | hsa_circ_023430 |
| hsa_circ_038734 | hsa_circ_023447 |
| hsa_circ_038735 | hsa_circ_023448 |
| hsa_circ_038736 | hsa_circ_023449 |
| hsa_circ_003096 | hsa_circ_023450 |
| hsa_circ_039021 | hsa_circ_007506 |
| hsa_circ_004082 | hsa_circ_023453 |
| hsa_c | hsa_c |

| | |
|---|---|
| irc_0038892 | irc_0023461 |
| hsa_circ_0038893 | hsa_circ_0023462 |
| hsa_circ_0038894 | hsa_circ_0002383 |
| hsa_circ_0038895 | hsa_circ_0023470 |
| hsa_circ_0038896 | hsa_circ_0023471 |
| hsa_circ_0038907 | hsa_circ_0023579 |
| hsa_circ_0038908 | hsa_circ_0023580 |
| hsa_circ_0038909 | hsa_circ_0023581 |
| hsa_circ_0038910 | hsa_circ_0023668 |
| hsa_circ_0007742 | hsa_circ_0023673 |
| hsa_circ_0038964 | hsa_circ_0023674 |
| hsa_circ_0039036 | hsa_circ_0023718 |
| hsa_circ_0039037 | hsa_circ_0023720 |
| hsa_circ_0039088 | hsa_circ_0023721 |

| | |
|---|---|
| hsa_circ_0039095 | hsa_circ_0023722 |
| hsa_circ_0039098 | hsa_circ_0023731 |
| hsa_circ_0039109 | hsa_circ_0023723 |
| hsa_circ_0039123 | hsa_circ_0023730 |
| hsa_circ_0039124 | hsa_circ_0023725 |
| hsa_circ_0039192 | hsa_circ_0023726 |
| hsa_circ_0039213 | hsa_circ_0023727 |
| hsa_circ_0039354 | hsa_circ_0023754 |
| hsa_circ_0039355 | hsa_circ_0003615 |
| hsa_circ_0039356 | hsa_circ_0007767 |
| hsa_circ_0039357 | hsa_circ_0023763 |
| hsa_circ_0039358 | hsa_circ_0023764 |
| hsa_circ_0039359 | hsa_circ_0023765 |
| hsa_circ_00394 | hsa_circ_00237 |

| | |
|---|---|
| 67 | 66 |
| hsa_circ_0039468 | hsa_circ_0023767 |
| hsa_circ_0039469 | hsa_circ_0023768 |
| hsa_circ_0039470 | hsa_circ_0023769 |
| hsa_circ_0039602 | hsa_circ_0023773 |
| hsa_circ_0039723 | hsa_circ_0009016 |
| hsa_circ_0039724 | hsa_circ_0023997 |
| hsa_circ_0009167 | hsa_circ_0023998 |
| hsa_circ_0039858 | hsa_circ_0023999 |
| hsa_circ_0039861 | hsa_circ_0024000 |
| hsa_circ_0039973 | hsa_circ_0024001 |
| hsa_circ_0039974 | hsa_circ_0024002 |
| hsa_circ_0040153 | hsa_circ_0024003 |
| hsa_circ_0040247 | hsa_circ_0024010 |
| hsa_c | hsa_c |

| | |
|---|---|
| irc_0040250 | irc_0024011 |
| hsa_circ_0040261 | hsa_circ_0024013 |
| hsa_circ_0040277 | hsa_circ_0024014 |
| hsa_circ_0040281 | hsa_circ_0024089 |
| hsa_circ_0040293 | hsa_circ_0024149 |
| hsa_circ_0040295 | hsa_circ_0024150 |
| hsa_circ_0040337 | hsa_circ_0024151 |
| hsa_circ_0040365 | hsa_circ_0024268 |
| hsa_circ_0003315 | hsa_circ_0024269 |
| hsa_circ_0040456 | hsa_circ_0024270 |
| hsa_circ_0040459 | hsa_circ_0024271 |
| hsa_circ_0040460 | hsa_circ_0024272 |
| hsa_circ_0040461 | hsa_circ_0024331 |
| hsa_circ_0040462 | hsa_circ_0024332 |

| | |
|---|---|
| hsa_circ_0040463 | hsa_circ_0008918 |
| hsa_circ_0040474 | hsa_circ_0024497 |
| hsa_circ_0040475 | hsa_circ_0024499 |
| hsa_circ_0040476 | hsa_circ_0024500 |
| hsa_circ_0040477 | hsa_circ_0024501 |
| hsa_circ_0040478 | hsa_circ_0024502 |
| hsa_circ_0040479 | hsa_circ_0024540 |
| hsa_circ_0040504 | hsa_circ_0024543 |
| hsa_circ_0040508 | hsa_circ_0024544 |
| hsa_circ_0040509 | hsa_circ_0003391 |
| hsa_circ_0040510 | hsa_circ_0007575 |
| hsa_circ_0040511 | hsa_circ_0024697 |
| hsa_circ_0040512 | hsa_circ_0024698 |
| hsa_circ_0405 | hsa_circ_0246 |

| | |
|---|---|
| 66 | 99 |
| hsa_circ_0040659 | hsa_circ_0024700 |
| hsa_circ_0040660 | hsa_circ_0024701 |
| hsa_circ_0040734 | hsa_circ_0024702 |
| hsa_circ_0040735 | hsa_circ_0024703 |
| hsa_circ_0040737 | hsa_circ_0024704 |
| hsa_circ_0040775 | hsa_circ_0004798 |
| hsa_circ_0040776 | hsa_circ_0006239 |
| hsa_circ_0040778 | hsa_circ_0024724 |
| hsa_circ_0040779 | hsa_circ_0024725 |
| hsa_circ_0040780 | hsa_circ_0024726 |
| hsa_circ_0040892 | hsa_circ_0024727 |
| hsa_circ_0040893 | hsa_circ_0024728 |
| hsa_circ_0040894 | hsa_circ_0024793 |
| hsa_c | hsa_c |

| | |
|---|---|
| irc_0040895 | irc_0002019 |
| hsa_circ_0040923 | hsa_circ_0002020 |
| hsa_circ_0040925 | hsa_circ_0024821 |
| hsa_circ_0040922 | hsa_circ_0002984 |
| hsa_circ_0040978 | hsa_circ_0005790 |
| hsa_circ_0040979 | hsa_circ_0024838 |
| hsa_circ_0004931 | hsa_circ_0024841 |
| hsa_circ_0041177 | hsa_circ_0024842 |
| hsa_circ_0041180 | hsa_circ_0024843 |
| hsa_circ_0007919 | hsa_circ_0024886 |
| hsa_circ_0041181 | hsa_circ_0024887 |
| hsa_circ_0041183 | hsa_circ_0024888 |
| hsa_circ_0041275 | hsa_circ_0024921 |
| hsa_circ_0041276 | hsa_circ_0024922 |

| | |
|---|---|
| hsa_circ_0041277 | hsa_circ_0007035 |
| hsa_circ_0041282 | hsa_circ_0025048 |
| hsa_circ_0041286 | hsa_circ_0025078 |
| hsa_circ_0041287 | hsa_circ_0025080 |
| hsa_circ_0041289 | hsa_circ_0025081 |
| hsa_circ_0041292 | hsa_circ_0025082 |
| hsa_circ_0041294 | hsa_circ_0025084 |
| hsa_circ_0041305 | hsa_circ_0025085 |
| hsa_circ_0041307 | hsa_circ_0025086 |
| hsa_circ_0041308 | hsa_circ_0025087 |
| hsa_circ_0041309 | hsa_circ_0025088 |
| hsa_circ_0041311 | hsa_circ_0025091 |
| hsa_circ_0041312 | hsa_circ_0025149 |
| hsa_circ_00413 | hsa_circ_00251 |

| | |
|---|---|
| 13 | 50 |
| hsa_circ_0041314 | hsa_circ_0025155 |
| hsa_circ_0041315 | hsa_circ_0025157 |
| hsa_circ_0041316 | hsa_circ_0025158 |
| hsa_circ_0041342 | hsa_circ_0025159 |
| hsa_circ_0041343 | hsa_circ_0025160 |
| hsa_circ_0041344 | hsa_circ_0025161 |
| hsa_circ_0041367 | hsa_circ_0025162 |
| hsa_circ_0041423 | hsa_circ_0025163 |
| hsa_circ_0041430 | hsa_circ_0025214 |
| hsa_circ_0041434 | hsa_circ_0025260 |
| hsa_circ_0041435 | hsa_circ_0025261 |
| hsa_circ_0041436 | hsa_circ_0025262 |
| hsa_circ_0041440 | hsa_circ_0025263 |
| hsa_c | hsa_c |

| | |
|---|---|
| irc_0002899 | irc_0025325 |
| hsa_circ_0041483 | hsa_circ_0025327 |
| hsa_circ_0041484 | hsa_circ_0025338 |
| hsa_circ_0041522 | hsa_circ_0025339 |
| hsa_circ_0041620 | hsa_circ_0025340 |
| hsa_circ_0041621 | hsa_circ_0002373 |
| hsa_circ_0041622 | hsa_circ_0025411 |
| hsa_circ_0041623 | hsa_circ_0025412 |
| hsa_circ_0041624 | hsa_circ_0025413 |
| hsa_circ_0041625 | hsa_circ_0025414 |
| hsa_circ_0041628 | hsa_circ_0025495 |
| hsa_circ_0041629 | hsa_circ_0025504 |
| hsa_circ_0041649 | hsa_circ_0025505 |
| hsa_circ_0041650 | hsa_circ_0025506 |

| | |
|---|---|
| hsa_circ_0041741 | hsa_circ_0025507 |
| hsa_circ_0041742 | hsa_circ_0025604 |
| hsa_circ_0041743 | hsa_circ_0025624 |
| hsa_circ_0041841 | hsa_circ_0025635 |
| hsa_circ_0041842 | hsa_circ_0005234 |
| hsa_circ_0041852 | hsa_circ_0009017 |
| hsa_circ_0041893 | hsa_circ_0025733 |
| hsa_circ_0041894 | hsa_circ_0025734 |
| hsa_circ_0041895 | hsa_circ_0010442 |
| hsa_circ_0041896 | hsa_circ_0025738 |
| hsa_circ_0041897 | hsa_circ_0025740 |
| hsa_circ_0041898 | hsa_circ_0025741 |
| hsa_circ_0041995 | hsa_circ_0006742 |
| hsa_circ_00419 | hsa_circ_00202 |

| | |
|---|---|
| 98 | 7 |
| hsa_circ_0041999 | hsa_circ_0025749 |
| hsa_circ_0042001 | hsa_circ_0025756 |
| hsa_circ_0042002 | hsa_circ_0025757 |
| hsa_circ_0042003 | hsa_circ_0025758 |
| hsa_circ_0042005 | hsa_circ_0025759 |
| hsa_circ_0042006 | hsa_circ_0025760 |
| hsa_circ_0042113 | hsa_circ_0025761 |
| hsa_circ_0007832 | hsa_circ_0025762 |
| hsa_circ_0042119 | hsa_circ_0025763 |
| hsa_circ_0042120 | hsa_circ_0025764 |
| hsa_circ_0042146 | hsa_circ_0025783 |
| hsa_circ_0042147 | hsa_circ_0025785 |
| hsa_circ_0042148 | hsa_circ_0025795 |
| hsa_c | hsa_c |

| | |
|---|---|
| irc_0042149 | irc_001044 |
| hsa_circ_0042150 | hsa_circ_0025921 |
| hsa_circ_0042408 | hsa_circ_0025952 |
| hsa_circ_0042409 | hsa_circ_0025953 |
| hsa_circ_0042410 | hsa_circ_0025954 |
| hsa_circ_0042415 | hsa_circ_0025955 |
| hsa_circ_0042419 | hsa_circ_0025957 |
| hsa_circ_0042421 | hsa_circ_0025958 |
| hsa_circ_0042730 | hsa_circ_0025988 |
| hsa_circ_0042774 | hsa_circ_0025989 |
| hsa_circ_0003422 | hsa_circ_0025990 |
| hsa_circ_0004659 | hsa_circ_0025991 |
| hsa_circ_0007194 | hsa_circ_0025992 |
| hsa_circ_0005565 | hsa_circ_0025996 |

| | |
|---|---|
| hsa_circ_0043022 | hsa_circ_0025997 |
| hsa_circ_0043026 | hsa_circ_0025998 |
| hsa_circ_0043027 | hsa_circ_0026082 |
| hsa_circ_0043028 | hsa_circ_0026083 |
| hsa_circ_0043029 | hsa_circ_0026114 |
| hsa_circ_0043030 | hsa_circ_0026116 |
| hsa_circ_0043031 | hsa_circ_0026117 |
| hsa_circ_0043201 | hsa_circ_0026148 |
| hsa_circ_0043202 | hsa_circ_0026161 |
| hsa_circ_0043220 | hsa_circ_0026178 |
| hsa_circ_0043225 | hsa_circ_0026179 |
| hsa_circ_0043226 | hsa_circ_0026180 |
| hsa_circ_0043227 | hsa_circ_0026202 |
| hsa_circ_00432 | hsa_circ_00262 |

| | |
|---|---|
| 28 | 03 |
| hsa_circ_0043231 | hsa_circ_0026204 |
| hsa_circ_0043232 | hsa_circ_0026205 |
| hsa_circ_0043234 | hsa_circ_0026208 |
| hsa_circ_0043235 | hsa_circ_0026209 |
| hsa_circ_0043236 | hsa_circ_0026242 |
| hsa_circ_0043237 | hsa_circ_0026245 |
| hsa_circ_0043238 | hsa_circ_0026246 |
| hsa_circ_0043239 | hsa_circ_0026247 |
| hsa_circ_0043387 | hsa_circ_0026248 |
| hsa_circ_0043390 | hsa_circ_0026249 |
| hsa_circ_0043411 | hsa_circ_0026250 |
| hsa_circ_0043412 | hsa_circ_0026251 |
| hsa_circ_0043499 | hsa_circ_0026252 |
| hsa_c | hsa_c |

| | |
|---|---|
| irc_0043534 | irc_0026253 |
| hsa_circ_0043535 | hsa_circ_0026303 |
| hsa_circ_0043536 | hsa_circ_0026307 |
| hsa_circ_0043537 | hsa_circ_0026310 |
| hsa_circ_0043538 | hsa_circ_0026475 |
| hsa_circ_0043539 | hsa_circ_0026476 |
| hsa_circ_0043540 | hsa_circ_0026480 |
| hsa_circ_0043541 | hsa_circ_0026577 |
| hsa_circ_0043543 | hsa_circ_0026579 |
| hsa_circ_0043545 | hsa_circ_0026591 |
| hsa_circ_0043547 | hsa_circ_0026610 |
| hsa_circ_0043548 | hsa_circ_0026611 |
| hsa_circ_0043549 | hsa_circ_0026612 |
| hsa_circ_0007248 | hsa_circ_0026614 |

| | |
|---|---|
| hsa_circ_004369 2 | hsa_circ_002661 5 |
| hsa_circ_004369 3 | hsa_circ_002661 6 |
| hsa_circ_004369 4 | hsa_circ_002661 7 |
| hsa_circ_004369 5 | hsa_circ_002661 8 |
| hsa_circ_004369 6 | hsa_circ_002661 9 |
| hsa_circ_004380 0 | hsa_circ_002662 1 |
| hsa_circ_004380 1 | hsa_circ_002662 3 |
| hsa_circ_004380 2 | hsa_circ_002662 4 |
| hsa_circ_004380 3 | hsa_circ_002662 6 |
| hsa_circ_004380 5 | hsa_circ_002662 7 |
| hsa_circ_004380 6 | hsa_circ_002663 5 |
| hsa_circ_004380 7 | hsa_circ_002667 1 |
| hsa_circ_004380 8 | hsa_circ_002667 2 |
| hsa_circ_0 0438 | hsa_circ_0 0266 |

| | |
|---|---|
| 09 | 91 |
| hsa_circ_0043810 | hsa_circ_0026692 |
| hsa_circ_0043811 | hsa_circ_0026736 |
| hsa_circ_0043875 | hsa_circ_0026737 |
| hsa_circ_0043877 | hsa_circ_0026738 |
| hsa_circ_0043878 | hsa_circ_0026739 |
| hsa_circ_0043879 | hsa_circ_0026740 |
| hsa_circ_0043880 | hsa_circ_0026744 |
| hsa_circ_0043881 | hsa_circ_0026745 |
| hsa_circ_0043882 | hsa_circ_0026746 |
| hsa_circ_0043885 | hsa_circ_0026752 |
| hsa_circ_0043886 | hsa_circ_0026753 |
| hsa_circ_0043888 | hsa_circ_0026783 |
| hsa_circ_0043889 | hsa_circ_0026803 |
| hsa_c | hsa_c |

| | |
|---|---|
| irc_0043924 | irc_0026897 |
| hsa_circ_0043925 | hsa_circ_0026923 |
| hsa_circ_0043934 | hsa_circ_0026924 |
| hsa_circ_0043938 | hsa_circ_0026925 |
| hsa_circ_0043939 | hsa_circ_0026926 |
| hsa_circ_0043940 | hsa_circ_0026927 |
| hsa_circ_0043941 | hsa_circ_0026928 |
| hsa_circ_0043942 | hsa_circ_0026929 |
| hsa_circ_0043943 | hsa_circ_0026991 |
| hsa_circ_0043944 | hsa_circ_0026992 |
| hsa_circ_0043946 | hsa_circ_0026993 |
| hsa_circ_0043948 | hsa_circ_0026994 |
| hsa_circ_0043951 | hsa_circ_0026995 |
| hsa_circ_0043956 | hsa_circ_0026996 |

| | | |
|---|---|---|
| | hsa_circ_0043957 | hsa_circ_0026997 |
| | hsa_circ_0043958 | hsa_circ_0027100 |
| | hsa_circ_0043980 | hsa_circ_0027101 |
| | hsa_circ_0043990 | hsa_circ_0027105 |
| | hsa_circ_0043995 | hsa_circ_0027103 |
| | hsa_circ_0044142 | hsa_circ_0027104 |
| | hsa_circ_0044178 | hsa_circ_0027128 |
| | hsa_circ_0044179 | hsa_circ_0027242 |
| | hsa_circ_0044181 | sa_circ_0027245 |
| | hsa_circ_0002960 | hsa_circ_0027246 |
| | hsa_circ_0004422 | hsa_circ_0027251 |
| | hsa_circ_0005940 | hsa_circ_0027252 |
| | hsa_circ_0044182 | hsa_circ_0027253 |
| | hsa_circ_0044 | hsa_circ_0272 |

| | |
|---|---|
| 83 | 54 |
| hsa_circ_0044184 | hsa_circ_0027304 |
| hsa_circ_0044185 | hsa_circ_0027305 |
| hsa_circ_0044186 | hsa_circ_0027323 |
| hsa_circ_0044187 | hsa_circ_0027324 |
| hsa_circ_0044330 | hsa_circ_0027382 |
| hsa_circ_0007293 | hsa_circ_0027383 |
| hsa_circ_0007565 | hsa_circ_0027395 |
| hsa_circ_0044346 | hsa_circ_0004448 |
| hsa_circ_0044347 | hsa_circ_0027489 |
| hsa_circ_0044348 | hsa_circ_0027491 |
| hsa_circ_0044362 | hsa_circ_0027492 |
| hsa_circ_0044398 | hsa_circ_0027493 |
| hsa_circ_0044401 | hsa_circ_0027494 |
| hsa_c | hsa_c |

| | |
|---|---|
| irc_0044403 | irc_01371 |
| hsa_circ_0044404 | hsa_circ_027517 |
| hsa_circ_0044579 | hsa_circ_027519 |
| hsa_circ_0044580 | hsa_circ_027520 |
| hsa_circ_0044581 | hsa_circ_027521 |
| hsa_circ_0044597 | hsa_circ_027522 |
| hsa_circ_0044594 | hsa_circ_027523 |
| sa_circ_0044710 | hsa_circ_027524 |
| hsa_circ_0044712 | hsa_circ_027532 |
| hsa_circ_0044714 | hsa_circ_027533 |
| hsa_circ_0044715 | hsa_circ_027566 |
| hsa_circ_0044716 | hsa_circ_027567 |
| hsa_circ_0044717 | hsa_circ_027568 |
| hsa_circ_0044718 | hsa_circ_027573 |

| | |
|---|---|
| hsa_circ_0044719 | hsa_circ_0027576 |
| hsa_circ_0044723 | hsa_circ_0027673 |
| hsa_circ_0044732 | hsa_circ_0027680 |
| hsa_circ_0044733 | hsa_circ_0027689 |
| hsa_circ_0044749 | hsa_circ_0027690 |
| hsa_circ_0044750 | hsa_circ_0027692 |
| hsa_circ_0044751 | hsa_circ_0027693 |
| hsa_circ_0044752 | hsa_circ_0027694 |
| hsa_circ_0044753 | hsa_circ_0027696 |
| hsa_circ_0044757 | hsa_circ_0027704 |
| hsa_circ_0044855 | hsa_circ_0027711 |
| hsa_circ_0044862 | hsa_circ_0027771 |
| hsa_circ_0044864 | hsa_circ_0027780 |
| hsa_circ_00448 | hsa_circ_00277 |

| | |
|---|---|
| 65 | 82 |
| hsa_circ_004492 | hsa_circ_002829 |
| hsa_circ_004493 | hsa_circ_002830 |
| hsa_circ_004527 | hsa_circ_002832 |
| hsa_circ_004528 | hsa_circ_002833 |
| hsa_circ_004529 | hsa_circ_002881 |
| hsa_circ_004563 | hsa_circ_002884 |
| hsa_circ_004566 | hsa_circ_002888 |
| hsa_circ_004567 | hsa_circ_002889 |
| hsa_circ_004568 | hsa_circ_002890 |
| hsa_circ_004569 | hsa_circ_002891 |
| hsa_circ_004570 | hsa_circ_002893 |
| hsa_circ_004571 | hsa_circ_002895 |
| hsa_circ_004572 | hsa_circ_002892 |

| | |
|---|---|
| hsa_circ_0045073 | hsa_circ_0027965 |
| hsa_circ_0045074 | hsa_circ_0027988 |
| hsa_circ_0045076 | hsa_circ_0028023 |
| hsa_circ_0045077 | hsa_circ_0028047 |
| hsa_circ_0045078 | hsa_circ_0028049 |
| hsa_circ_0045079 | hsa_circ_0028051 |
| hsa_circ_0045080 | hsa_circ_0028052 |
| hsa_circ_0045081 | hsa_circ_0028053 |
| hsa_circ_0045082 | hsa_circ_0002678 |
| hsa_circ_0045083 | hsa_circ_0004715 |
| hsa_circ_0045084 | hsa_circ_0006852 |
| hsa_circ_0045085 | hsa_circ_0008092 |
| hsa_circ_0045221 | hsa_circ_0028062 |

| | |
|---|---|
| hsa_circ_0007534 | hsa_circ_0028063 |
| hsa_circ_0045222 | hsa_circ_0028064 |
| hsa_circ_0045223 | hsa_circ_0028065 |
| hsa_circ_0045224 | hsa_circ_0028066 |
| hsa_circ_0045225 | hsa_circ_0028067 |
| hsa_circ_0045226 | hsa_circ_0021667 |
| hsa_circ_0045335 | hsa_circ_0028102 |
| hsa_circ_0045369 | hsa_circ_0028108 |
| hsa_circ_0045370 | hsa_circ_0028110 |
| hsa_circ_0045391 | hsa_circ_0028111 |
| hsa_circ_0045423 | hsa_circ_0002628 |
| hsa_circ_0045426 | hsa_circ_0028170 |
| hsa_circ_0045427 | hsa_circ_0028173 |
| hsa_circ_00454 | hsa_circ_00281 |

| | |
|---|---|
| 28 | 74 |
| hsa_circ_0045437 | hsa_circ_0028175 |
| hsa_circ_0045442 | hsa_circ_0028176 |
| hsa_circ_0045444 | hsa_circ_0028177 |
| hsa_circ_0045445 | hsa_circ_0028309 |
| hsa_circ_0045487 | hsa_circ_0028311 |
| hsa_circ_0045521 | hsa_circ_0028312 |
| hsa_circ_0045617 | hsa_circ_0028313 |
| hsa_circ_0045622 | hsa_circ_0028314 |
| hsa_circ_0045623 | hsa_circ_0028315 |
| hsa_circ_0045624 | hsa_circ_0028316 |
| hsa_circ_0045667 | hsa_circ_0003425 |
| hsa_circ_0045670 | hsa_circ_0004186 |
| hsa_circ_0045672 | hsa_circ_0028417 |
| hsa_c | hsa_c |

| | |
|---|---|
| irc_0045673 | irc_0028418 |
| hsa_circ_0045674 | hsa_circ_0028419 |
| hsa_circ_0045675 | hsa_circ_0028420 |
| hsa_circ_0045782 | hsa_circ_0028421 |
| hsa_circ_0045830 | hsa_circ_0028422 |
| hsa_circ_0045918 | hsa_circ_0028423 |
| hsa_circ_0045919 | hsa_circ_0028424 |
| hsa_circ_0045945 | hsa_circ_0028471 |
| hsa_circ_0045967 | hsa_circ_0028532 |
| hsa_circ_0046045 | hsa_circ_0028549 |
| hsa_circ_0046096 | hsa_circ_0028550 |
| hsa_circ_0046139 | hsa_circ_0028551 |
| hsa_circ_0046138 | hsa_circ_0028552 |
| hsa_circ_0005221 | hsa_circ_0028558 |

| | |
|---|---|
| hsa_circ_0046158 | hsa_circ_0028559 |
| hsa_circ_0046159 | hsa_circ_0028553 |
| hsa_circ_0046160 | hsa_circ_0028554 |
| hsa_circ_0046161 | hsa_circ_0028555 |
| hsa_circ_0046169 | hsa_circ_0028556 |
| hsa_circ_0046170 | hsa_circ_0028557 |
| hsa_circ_0046171 | hsa_circ_0028589 |
| hsa_circ_0046172 | hsa_circ_0028590 |
| hsa_circ_0046173 | hsa_circ_0029024 |
| hsa_circ_0046174 | hsa_circ_0029203 |
| hsa_circ_0046175 | hsa_circ_0029204 |
| hsa_circ_0046176 | hsa_circ_0029289 |
| hsa_circ_0046179 | hsa_circ_0029438 |
| hsa_circ_00461 | hsa_circ_00294 |

| | |
|---|---|
| 80 | 39 |
| hsa_circ_0046181 | hsa_circ_0029440 |
| hsa_circ_0046184 | hsa_circ_0029441 |
| hsa_circ_0046185 | hsa_circ_0029455 |
| hsa_circ_0046186 | hsa_circ_0029456 |
| hsa_circ_0046187 | hsa_circ_0029457 |
| hsa_circ_0046188 | hsa_circ_0029458 |
| hsa_circ_0046189 | hsa_circ_0029482 |
| hsa_circ_0046190 | hsa_circ_0029483 |
| hsa_circ_0046191 | hsa_circ_0029489 |
| hsa_circ_0046194 | hsa_circ_0029490 |
| hsa_circ_0046195 | hsa_circ_0029491 |
| hsa_circ_0046196 | hsa_circ_0029492 |
| hsa_circ_0046197 | hsa_circ_0029493 |
| hsa_c | hsa_c |

| | |
|---|---|
| irc_0046198 | irc_0029494 |
| hsa_circ_0046199 | hsa_circ_0029495 |
| hsa_circ_0046200 | hsa_circ_0029496 |
| hsa_circ_0046201 | hsa_circ_0029497 |
| hsa_circ_0006333 | hsa_circ_0029498 |
| hsa_circ_0046295 | hsa_circ_0029499 |
| hsa_circ_0046296 | hsa_circ_0006481 |
| hsa_circ_0046297 | hsa_circ_0006833 |
| hsa_circ_0046331 | hsa_circ_0008007 |
| hsa_circ_0046332 | hsa_circ_0029649 |
| hsa_circ_0046333 | hsa_circ_0029650 |
| hsa_circ_0046334 | hsa_circ_0029651 |
| hsa_circ_0046335 | hsa_circ_0029652 |
| hsa_circ_0046336 | hsa_circ_0029653 |

| | |
|---|---|
| hsa_circ_0046337 | hsa_circ_0029655 |
| hsa_circ_0046338 | hsa_circ_0029657 |
| hsa_circ_0046339 | hsa_circ_0029658 |
| hsa_circ_0046342 | hsa_circ_0029661 |
| hsa_circ_0046343 | hsa_circ_0008763 |
| hsa_circ_0046344 | hsa_circ_0003408 |
| hsa_circ_0046345 | hsa_circ_0029715 |
| hsa_circ_0046412 | hsa_circ_0029716 |
| hsa_circ_0046413 | hsa_circ_0029717 |
| hsa_circ_0046423 | hsa_circ_0029718 |
| hsa_circ_0046424 | hsa_circ_0029783 |
| hsa_circ_0046425 | hsa_circ_0029784 |
| hsa_circ_0046426 | hsa_circ_0029785 |
| hsa_circ_00464 | hsa_circ_00297 |

| | |
|---|---|
| 27 | 86 |
| hsa_circ_004628 | hsa_circ_029787 |
| hsa_circ_004629 | hsa_circ_029788 |
| hsa_circ_004630 | hsa_circ_029789 |
| hsa_circ_001758 | hsa_circ_029796 |
| hsa_circ_001759 | hsa_circ_003489 |
| hsa_circ_007942 | hsa_circ_029807 |
| hsa_circ_046439 | hsa_circ_029891 |
| hsa_circ_046440 | hsa_circ_029895 |
| hsa_circ_046442 | hsa_circ_029900 |
| hsa_circ_046443 | hsa_circ_029906 |
| hsa_circ_046712 | hsa_circ_030021 |
| hsa_circ_046715 | hsa_circ_030044 |
| hsa_circ_046719 | hsa_circ_030043 |
| hsa_c | hsa_c |

| | |
|---|---|
| irc_0046734 | irc_0030126 |
| hsa_circ_0046735 | hsa_circ_0030194 |
| hsa_circ_0046737 | hsa_circ_0030232 |
| hsa_circ_0046738 | hsa_circ_0030277 |
| hsa_circ_0046741 | hsa_circ_0005980 |
| hsa_circ_0046742 | hsa_circ_0030286 |
| hsa_circ_0046743 | hsa_circ_0030287 |
| hsa_circ_0046744 | hsa_circ_0030288 |
| hsa_circ_0046847 | hsa_circ_001475 |
| hsa_circ_0046850 | hsa_circ_0030353 |
| hsa_circ_0046851 | hsa_circ_0030354 |
| hsa_circ_0046852 | hsa_circ_0030355 |
| hsa_circ_0006400 | hsa_circ_0030400 |
| hsa_circ_0046953 | hsa_circ_0030461 |

| | |
|---|---|
| hsa_circ_0046956 | hsa_circ_0030462 |
| hsa_circ_0046965 | hsa_circ_0030463 |
| hsa_circ_0046966 | hsa_circ_0030465 |
| hsa_circ_0046967 | hsa_circ_0030467 |
| hsa_circ_0047045 | hsa_circ_0030468 |
| hsa_circ_0047046 | hsa_circ_0030470 |
| hsa_circ_0047048 | hsa_circ_0030471 |
| hsa_circ_0047054 | hsa_circ_0030472 |
| hsa_circ_0047059 | hsa_circ_0030473 |
| hsa_circ_0047084 | hsa_circ_0030545 |
| hsa_circ_0047128 | hsa_circ_0030547 |
| hsa_circ_0047139 | hsa_circ_0030548 |
| hsa_circ_0047140 | hsa_circ_0030549 |
| hsa_circ_00471 | hsa_circ_00305 |

| | |
|---|---|
| 41 | 50 |
| hsa_circ_0047142 | hsa_circ_0030551 |
| hsa_circ_0003495 | hsa_circ_0030552 |
| hsa_circ_0047298 | hsa_circ_0030746 |
| hsa_circ_0047304 | hsa_circ_0030747 |
| hsa_circ_0047305 | hsa_circ_0030748 |
| hsa_circ_0047306 | hsa_circ_0030749 |
| hsa_circ_0047318 | hsa_circ_0030750 |
| hsa_circ_0047381 | hsa_circ_0030744 |
| hsa_circ_0047559 | hsa_circ_0030780 |
| hsa_circ_0047560 | hsa_circ_0030782 |
| hsa_circ_0047561 | hsa_circ_0030786 |
| hsa_circ_0047562 | hsa_circ_0030787 |
| hsa_circ_0047563 | hsa_circ_0008744 |
| hsa_c | hsa_c |

| | |
|---|---|
| irc_0047564 | irc_0030795 |
| hsa_circ_0047565 | hsa_circ_0030797 |
| hsa_circ_0047566 | hsa_circ_0030801 |
| hsa_circ_0047567 | hsa_circ_0030802 |
| hsa_circ_0047568 | hsa_circ_0030803 |
| hsa_circ_0047570 | hsa_circ_0030804 |
| hsa_circ_0006952 | hsa_circ_0030805 |
| hsa_circ_0047707 | hsa_circ_0030806 |
| hsa_circ_0047708 | hsa_circ_0030807 |
| hsa_circ_0047709 | hsa_circ_0007314 |
| hsa_circ_0047711 | hsa_circ_0030820 |
| hsa_circ_0003423 | hsa_circ_0030821 |
| hsa_circ_0004371 | hsa_circ_00828 |
| hsa_circ_0007098 | hsa_circ_0030834 |

| | |
|---|---|
| hsa_circ_0047809 | hsa_circ_0030855 |
| hsa_circ_0047810 | hsa_circ_0030856 |
| hsa_circ_0047812 | hsa_circ_0030857 |
| hsa_circ_0047813 | hsa_circ_0030864 |
| hsa_circ_0047815 | hsa_circ_0030869 |
| hsa_circ_0047816 | hsa_circ_0030873 |
| hsa_circ_0047818 | hsa_circ_0030874 |
| hsa_circ_0047819 | hsa_circ_0030877 |
| hsa_circ_0047820 | hsa_circ_0030878 |
| hsa_circ_0047821 | hsa_circ_0030920 |
| hsa_circ_0047822 | hsa_circ_0030938 |
| hsa_circ_0047823 | hsa_circ_0030939 |
| hsa_circ_0047827 | hsa_circ_0030940 |
| hsa_circ_00478 | hsa_circ_00309 |

| | |
|---|---|
| 28 | 42 |
| hsa_circ_0047890 | hsa_circ_0030944 |
| hsa_circ_0047893 | hsa_circ_0030945 |
| hsa_circ_0047894 | hsa_circ_0030946 |
| hsa_circ_0047895 | hsa_circ_0030947 |
| hsa_circ_0047896 | hsa_circ_0030948 |
| hsa_circ_0047902 | hsa_circ_0030949 |
| hsa_circ_0047903 | hsa_circ_0030993 |
| hsa_circ_0047904 | hsa_circ_0030997 |
| hsa_circ_0047905 | hsa_circ_0031000 |
| hsa_circ_0047906 | hsa_circ_0031002 |
| hsa_circ_0002895 | hsa_circ_0031003 |
| hsa_circ_0003479 | hsa_circ_0031004 |
| hsa_circ_0005140 | hsa_circ_0031005 |
| hsa_c | hsa_c |

| | |
|---|---|
| irc_0007725 | irc_0031006 |
| hsa_circ_0048272 | hsa_circ_0031212 |
| hsa_circ_0048273 | hsa_circ_0031213 |
| hsa_circ_0048275 | hsa_circ_0031244 |
| hsa_circ_0048278 | hsa_circ_0031245 |
| hsa_circ_0048279 | hsa_circ_0031246 |
| hsa_circ_0048315 | hsa_circ_0031247 |
| hsa_circ_0048316 | hsa_circ_0031248 |
| hsa_circ_0048387 | hsa_circ_0031249 |
| hsa_circ_0048389 | hsa_circ_002126 |
| hsa_circ_0048390 | hsa_circ_000587 |
| hsa_circ_0048391 | hsa_circ_0031338 |
| hsa_circ_0048515 | hsa_circ_0031349 |
| hsa_circ_0048524 | hsa_circ_0031354 |

| | |
|---|---|
| hsa_circ_0048523 | hsa_circ_0031403 |
| hsa_circ_0048597 | hsa_circ_0031404 |
| hsa_circ_0048598 | hsa_circ_0031412 |
| hsa_circ_0048599 | hsa_circ_0002301 |
| hsa_circ_0048600 | hsa_circ_0031456 |
| hsa_circ_0006321 | hsa_circ_0031457 |
| hsa_circ_0048603 | hsa_circ_0031459 |
| hsa_circ_0048604 | hsa_circ_0031460 |
| hsa_circ_0048605 | hsa_circ_0031461 |
| hsa_circ_0048606 | hsa_circ_0031462 |
| hsa_circ_0048607 | hsa_circ_0031464 |
| hsa_circ_0048608 | hsa_circ_0031467 |
| hsa_circ_0019400 | hsa_circ_0031466 |
| hsa_circ_00487 | hsa_circ_00315 |

| | |
|---|---|
| 16 | 96 |
| hsa_circ_0048717 | hsa_circ_0031597 |
| hsa_circ_0048718 | hsa_circ_0031598 |
| hsa_circ_0048719 | hsa_circ_0031599 |
| hsa_circ_0048720 | hsa_circ_0031600 |
| hsa_circ_0048721 | hsa_circ_0031601 |
| hsa_circ_0048722 | hsa_circ_0031602 |
| hsa_circ_0048723 | hsa_circ_0031603 |
| hsa_circ_0048724 | hsa_circ_0031616 |
| hsa_circ_0048725 | hsa_circ_0031617 |
| hsa_circ_0048726 | hsa_circ_0031618 |
| hsa_circ_0048968 | hsa_circ_0031619 |
| hsa_circ_0048972 | hsa_circ_0031620 |
| hsa_circ_0049097 | hsa_circ_0031621 |
| hsa_c | hsa_c |

| | |
|---|---|
| irc_0049100 | irc_0031622 |
| hsa_circ_0049136 | hsa_circ_0031623 |
| hsa_circ_0005123 | hsa_circ_0031626 |
| hsa_circ_0049366 | hsa_circ_0031625 |
| hsa_circ_0049373 | hsa_circ_0031739 |
| hsa_circ_0049415 | hsa_circ_0031779 |
| hsa_circ_0049416 | hsa_circ_0031780 |
| hsa_circ_0049417 | hsa_circ_0031781 |
| hsa_circ_0049418 | hsa_circ_0031782 |
| hsa_circ_0049419 | hsa_circ_0002819 |
| hsa_circ_0049476 | hsa_circ_0003616 |
| hsa_circ_0049472 | hsa_circ_0003689 |
| hsa_circ_0049488 | hsa_circ_0005215 |
| hsa_circ_0049489 | hsa_circ_0006066 |

| | |
|---|---|
| hsa_circ_004990 | hsa_circ_008088 |
| hsa_circ_004991 | hsa_circ_008845 |
| hsa_circ_004976 2 | hsa_circ_009080 |
| hsa_circ_0049763 | hsa_circ_0031772 |
| hsa_circ_0049764 | hsa_circ_0002098 |
| hsa_circ_0049837 | hsa_circ_0002667 |
| hsa_circ_0049858 | hsa_circ_0004055 |
| hsa_circ_0049897 | hsa_circ_0007220 |
| hsa_circ_0049937 | hsa_circ_0007999 |
| hsa_circ_0049984 | hsa_circ_0008588 |
| hsa_circ_0049985 | hsa_circ_0008862 |
| hsa_circ_0049986 | hsa_circ_0008909 |
| hsa_circ_0049987 | hsa_circ_0006940 |
| hsa_circ_00499 | hsa_circ_00318 |

| | |
|---|---|
| 88 | 18 |
| hsa_circ_0050232 | hsa_circ_0031819 |
| hsa_circ_0050235 | hsa_circ_0031820 |
| hsa_circ_0050237 | hsa_circ_0031821 |
| hsa_circ_0050238 | hsa_circ_0031822 |
| hsa_circ_0013977 | hsa_circ_0031823 |
| hsa_circ_0008347 | hsa_circ_0031824 |
| hsa_circ_0050564 | hsa_circ_0031837 |
| hsa_circ_0050567 | hsa_circ_0031854 |
| hsa_circ_0050582 | hsa_circ_0031855 |
| hsa_circ_0050766 | hsa_circ_0031856 |
| hsa_circ_0050769 | hsa_circ_0031941 |
| hsa_circ_0005081 | hsa_circ_0002202 |
| hsa_circ_0050791 | hsa_circ_0032020 |
| hsa_c | hsa_c |

| | |
|---|---|
| irc_0050792 | irc_0032054 |
| hsa_circ_0050793 | hsa_circ_0032056 |
| hsa_circ_0050794 | hsa_circ_0032085 |
| hsa_circ_0050795 | hsa_circ_0032160 |
| hsa_circ_0050798 | hsa_circ_0032162 |
| hsa_circ_0050799 | hsa_circ_0032216 |
| hsa_circ_0050801 | hsa_circ_0032217 |
| hsa_circ_0050802 | hsa_circ_0032234 |
| hsa_circ_0050803 | hsa_circ_0008468 |
| hsa_circ_0050804 | hsa_circ_0032251 |
| hsa_circ_0050805 | hsa_circ_0032250 |
| hsa_circ_0050806 | hsa_circ_0032284 |
| hsa_circ_0050958 | hsa_circ_001250 |
| hsa_circ_0050976 | hsa_circ_0032370 |

| | |
|---|---|
| hsa_circ_0050977 | hsa_circ_0004008 |
| hsa_circ_0050978 | hsa_circ_0032387 |
| hsa_circ_0050979 | hsa_circ_0032391 |
| hsa_circ_0050991 | hsa_circ_0032392 |
| hsa_circ_0050993 | hsa_circ_0032398 |
| hsa_circ_0051055 | hsa_circ_0032399 |
| hsa_circ_0051159 | hsa_circ_0032400 |
| hsa_circ_0051161 | hsa_circ_0032401 |
| hsa_circ_0051167 | hsa_circ_0032402 |
| hsa_circ_0051170 | hsa_circ_0032403 |
| hsa_circ_0051174 | hsa_circ_00594 |
| hsa_circ_0051175 | hsa_circ_0032511 |
| hsa_circ_0051178 | hsa_circ_0032521 |
| hsa_circ_0511 | hsa_circ_0325 |

| | |
|---|---|
| 80 | 28 |
| hsa_circ_0051181 | hsa_circ_0006218 |
| hsa_circ_0051187 | hsa_circ_0032537 |
| hsa_circ_0051193 | hsa_circ_0032539 |
| hsa_circ_0051194 | hsa_circ_0032540 |
| hsa_circ_0051197 | hsa_circ_0032548 |
| hsa_circ_0051198 | hsa_circ_0032550 |
| hsa_circ_0051201 | hsa_circ_0032623 |
| hsa_circ_0051203 | hsa_circ_0032697 |
| hsa_circ_0051204 | hsa_circ_0032698 |
| hsa_circ_0051205 | hsa_circ_0032701 |
| hsa_circ_0051327 | hsa_circ_0032702 |
| hsa_circ_0051416 | hsa_circ_0032705 |
| hsa_circ_0051504 | hsa_circ_0032706 |
| hsa_c | hsa_c |

| | |
|---|---|
| irc_0051509 | irc_0032707 |
| hsa_circ_0051510 | hsa_circ_0032713 |
| hsa_circ_0051646 | hsa_circ_0032714 |
| hsa_circ_0051687 | hsa_circ_0032715 |
| hsa_circ_0052104 | hsa_circ_0032757 |
| hsa_circ_0052258 | hsa_circ_0032805 |
| hsa_circ_0052259 | hsa_circ_0002499 |
| hsa_circ_001634 | hsa_circ_0032820 |
| hsa_circ_0052300 | hsa_circ_0032823 |
| hsa_circ_0052307 | hsa_circ_0032836 |
| hsa_circ_0052306 | hsa_circ_0032837 |
| hsa_circ_0052305 | hsa_circ_0032838 |
| hsa_circ_001635 | hsa_circ_0032839 |
| hsa_circ_0052309 | hsa_circ_0032844 |

| | |
|---|---|
| hsa_circ_0052332 | hsa_circ_0032845 |
| hsa_circ_0052333 | hsa_circ_0002559 |
| hsa_circ_0059276 | hsa_circ_0032892 |
| hsa_circ_0059277 | hsa_circ_0032893 |
| hsa_circ_0059285 | hsa_circ_0033003 |
| hsa_circ_0059287 | hsa_circ_0033004 |
| hsa_circ_0059294 | hsa_circ_0033077 |
| hsa_circ_0059295 | hsa_circ_0033078 |
| hsa_circ_0059296 | hsa_circ_0033079 |
| hsa_circ_0059297 | hsa_circ_0033080 |
| hsa_circ_0059298 | hsa_circ_0033081 |
| hsa_circ_0059299 | hsa_circ_0033082 |
| hsa_circ_0059300 | hsa_circ_0002398 |
| hsa_circ_00593 | hsa_circ_00079 |

| | |
|---|---|
| 84 | 79 |
| hsa_circ_0059385 | hsa_circ_0033209 |
| hsa_circ_0059386 | hsa_circ_0033211 |
| hsa_circ_0059395 | hsa_circ_0033212 |
| hsa_circ_0059494 | hsa_circ_0033218 |
| hsa_circ_0059534 | hsa_circ_0033221 |
| hsa_circ_0059536 | hsa_circ_0033225 |
| hsa_circ_0059537 | hsa_circ_0033227 |
| hsa_circ_0059538 | hsa_circ_0033230 |
| hsa_circ_0059539 | hsa_circ_0033537 |
| hsa_circ_0059540 | hsa_circ_0033538 |
| hsa_circ_0059541 | hsa_circ_0033606 |
| hsa_circ_0059609 | hsa_circ_0033607 |
| hsa_circ_0059633 | hsa_circ_0034089 |
| hsa_c | hsa_c |

| | |
|---|---|
| irc_0059689 | irc_0034092 |
| hsa_circ_0059778 | hsa_circ_0034327 |
| hsa_circ_0007029 | hsa_circ_0034340 |
| hsa_circ_002158 | hsa_circ_0034339 |
| hsa_circ_0059791 | hsa_circ_0034355 |
| hsa_circ_0059790 | hsa_circ_0034356 |
| hsa_circ_0060043 | hsa_circ_0034396 |
| hsa_circ_0060056 | hsa_circ_0034637 |
| hsa_circ_0060058 | hsa_circ_0034705 |
| hsa_circ_0060187 | hsa_circ_0034706 |
| hsa_circ_0060195 | hsa_circ_0034707 |
| hsa_circ_0060196 | hsa_circ_0034708 |
| hsa_circ_0060197 | hsa_circ_0034709 |
| hsa_circ_0060298 | hsa_circ_0034710 |

| | |
|---|---|
| hsa_circ_006301 | hsa_circ_034711 |
| hsa_circ_006303 | hsa_circ_034726 |
| hsa_circ_006324 | hsa_circ_034728 |
| hsa_circ_006325 | hsa_circ_034732 |
| hsa_circ_006326 | hsa_circ_034733 |
| hsa_circ_006391 | hsa_circ_034736 |
| hsa_circ_006393 | hsa_circ_034737 |
| hsa_circ_006481 | hsa_circ_034738 |
| hsa_circ_002517 | hsa_circ_034739 |
| hsa_circ_006524 | hsa_circ_034740 |
| hsa_circ_006525 | hsa_circ_034741 |
| hsa_circ_006526 | hsa_circ_034742 |
| hsa_circ_006529 | hsa_circ_034743 |

| | |
|---|---|
| hsa_circ_0060530 | hsa_circ_01756 |
| hsa_circ_0060562 | hsa_circ_0034996 |
| hsa_circ_0060666 | hsa_circ_0034997 |
| hsa_circ_0060667 | hsa_circ_0034998 |
| hsa_circ_0060668 | hsa_circ_0035132 |
| hsa_circ_0060669 | hsa_circ_0007487 |
| hsa_circ_0060670 | hsa_circ_0035211 |
| hsa_circ_0060674 | hsa_circ_0035212 |
| hsa_circ_0060675 | hsa_circ_0035223 |
| hsa_circ_0060676 | hsa_circ_0035224 |
| hsa_circ_0060677 | hsa_circ_0035225 |
| hsa_circ_0060678 | hsa_circ_0035229 |
| hsa_circ_0060679 | hsa_circ_0035233 |
| hsa_circ_0060[illegible] | hsa_circ_0035[illegible] |

| | |
|---|---|
| 80 | 34 |
| hsa_circ_0060681 | hsa_circ_0035278 |
| hsa_circ_0060682 | hsa_circ_0035301 |
| hsa_circ_0060683 | hsa_circ_0035304 |
| hsa_circ_0060685 | hsa_circ_0035307 |
| hsa_circ_0060686 | hsa_circ_0035308 |
| hsa_circ_0060687 | hsa_circ_0035310 |
| hsa_circ_0060688 | hsa_circ_0035341 |
| hsa_circ_0060689 | hsa_circ_0035342 |
| hsa_circ_0060690 | hsa_circ_0035343 |
| hsa_circ_0060691 | hsa_circ_0035344 |
| hsa_circ_0060692 | hsa_circ_0035345 |
| hsa_circ_0060693 | hsa_circ_0035389 |
| hsa_circ_0060695 | hsa_circ_0035393 |
| hsa_c | hsa_c |

| | |
|---|---|
| irc_0060696 | irc_0035394 |
| hsa_circ_0060697 | hsa_circ_0035395 |
| hsa_circ_0060698 | hsa_circ_0035398 |
| hsa_circ_0060699 | hsa_circ_0035399 |
| hsa_circ_0060700 | hsa_circ_0035400 |
| hsa_circ_0060701 | hsa_circ_0035401 |
| hsa_circ_0060702 | hsa_circ_0035402 |
| hsa_circ_0060703 | hsa_circ_0035403 |
| hsa_circ_0060706 | hsa_circ_0035404 |
| hsa_circ_0060707 | hsa_circ_0035472 |
| hsa_circ_0060704 | hsa_circ_0035473 |
| hsa_circ_0060788 | hsa_circ_0035474 |
| hsa_circ_0060789 | hsa_circ_0035475 |
| hsa_circ_0060790 | hsa_circ_0035478 |

| | |
|---|---|
| hsa_circ_0060791 | hsa_circ_0035477 |
| hsa_circ_0060792 | hsa_circ_0035496 |
| hsa_circ_0060793 | hsa_circ_0035497 |
| hsa_circ_0060794 | hsa_circ_0035498 |
| hsa_circ_0060795 | hsa_circ_0035499 |
| hsa_circ_0060796 | hsa_circ_0035500 |
| hsa_circ_0060835 | hsa_circ_0035501 |
| hsa_circ_0060836 | hsa_circ_0035506 |
| hsa_circ_0060945 | hsa_circ_0035507 |
| hsa_circ_0060946 | hsa_circ_0035503 |
| hsa_circ_0060947 | hsa_circ_0035504 |
| hsa_circ_0060948 | hsa_circ_0002495 |
| hsa_circ_0060980 | hsa_circ_0035659 |
| hsa_circ_00610 | hsa_circ_00356 |

| | |
|---|---|
| 07 | 60 |
| hsa_circ_0061120 | hsa_circ_0035886 |
| hsa_circ_0061118 | hsa_circ_00601 |
| hsa_circ_0061162 | hsa_circ_0035887 |
| hsa_circ_0061161 | hsa_circ_0035888 |
| hsa_circ_0061185 | hsa_circ_0035924 |
| hsa_circ_0061186 | hsa_circ_0035925 |
| hsa_circ_0061203 | hsa_circ_0035926 |
| hsa_circ_0061204 | hsa_circ_0035927 |
| hsa_circ_0061206 | hsa_circ_0035930 |
| hsa_circ_0061207 | hsa_circ_0035931 |
| hsa_circ_0061208 | hsa_circ_0036007 |
| hsa_circ_0061209 | hsa_circ_0036008 |
| hsa_circ_0061210 | hsa_circ_0036207 |
| hsa_c | hsa_c |

| | |
|---|---|
| irc_0061201 | irc_0036208 |
| hsa_circ_0061202 | hsa_circ_0036225 |
| hsa_circ_0003890 | hsa_circ_0036226 |
| hsa_circ_0061306 | hsa_circ_0036227 |
| hsa_circ_0061364 | hsa_circ_0036233 |
| hsa_circ_0061365 | hsa_circ_0036235 |
| hsa_circ_0006158 | hsa_circ_0036236 |
| hsa_circ_0007207 | hsa_circ_0036261 |
| hsa_circ_0061423 | hsa_circ_0036269 |
| hsa_circ_0061427 | hsa_circ_0036280 |
| hsa_circ_0061430 | hsa_circ_0036305 |
| hsa_circ_0061431 | hsa_circ_0036306 |
| hsa_circ_0061560 | hsa_circ_0036312 |
| hsa_circ_0061563 | hsa_circ_0036323 |

| | |
|---|---|
| hsa_circ_0061566 | hsa_circ_0036338 |
| hsa_circ_0061567 | hsa_circ_0036339 |
| hsa_circ_0061581 | hsa_circ_0036340 |
| hsa_circ_0005076 | hsa_circ_0036341 |
| hsa_circ_0061585 | hsa_circ_0036433 |
| hsa_circ_0061583 | hsa_circ_0036434 |
| hsa_circ_0061584 | hsa_circ_0036435 |
| hsa_circ_0002763 | hsa_circ_0036436 |
| hsa_circ_0061644 | hsa_circ_0002568 |
| hsa_circ_0061645 | hsa_circ_0036567 |
| hsa_circ_0061646 | hsa_circ_0036575 |
| hsa_circ_0061647 | hsa_circ_0036576 |
| hsa_circ_0061648 | hsa_circ_0036670 |
| hsa_circ_00616 | hsa_circ_00366 |

| | |
|---|---|
| 49 | 71 |
| hsa_circ_0061650 | hsa_circ_0036672 |
| hsa_circ_0061652 | hsa_circ_0007837 |
| hsa_circ_0061653 | hsa_circ_0036694 |
| hsa_circ_0061654 | hsa_circ_0036739 |
| hsa_circ_0061655 | hsa_circ_0036740 |
| hsa_circ_0061659 | hsa_circ_0036741 |
| hsa_circ_0061675 | hsa_circ_0036782 |
| hsa_circ_0061680 | hsa_circ_0036783 |
| hsa_circ_0061684 | hsa_circ_0036784 |
| hsa_circ_0061759 | hsa_circ_0036851 |
| hsa_circ_0061760 | hsa_circ_0036855 |
| hsa_circ_0061821 | hsa_circ_0036856 |
| hsa_circ_0061978 | hsa_circ_0036858 |
| hsa_c | hsa_c |

| | |
|---|---|
| irc_0061979 | irc_0036859 |
| hsa_circ_0061980 | hsa_circ_0036863 |
| hsa_circ_0061981 | hsa_circ_0036865 |
| hsa_circ_0061982 | hsa_circ_0036866 |
| hsa_circ_0061983 | hsa_circ_0036948 |
| hsa_circ_0062093 | hsa_circ_0036955 |
| hsa_circ_0062099 | hsa_circ_0005035 |
| hsa_circ_0062127 | hsa_circ_0037019 |
| hsa_circ_0003781 | hsa_circ_0037020 |
| hsa_circ_0062289 | hsa_circ_0037021 |
| hsa_circ_0062386 | hsa_circ_0037022 |
| hsa_circ_0062388 | hsa_circ_0037023 |
| hsa_circ_0062390 | hsa_circ_0037024 |
| hsa_circ_0062391 | hsa_circ_0037025 |

| | |
|---|---|
| hsa_circ_0062392 | hsa_circ_0037048 |
| hsa_circ_0062393 | hsa_circ_0037049 |
| hsa_circ_0062335 | hsa_circ_0037050 |
| hsa_circ_0062351 | hsa_circ_0037051 |
| hsa_circ_0062354 | hsa_circ_0037174 |
| hsa_circ_0062357 | hsa_circ_001482 |
| hsa_circ_0062360 | hsa_circ_0037176 |
| hsa_circ_0062370 | hsa_circ_0037179 |
| hsa_circ_0062371 | hsa_circ_0037180 |
| hsa_circ_0062377 | hsa_circ_0037209 |
| hsa_circ_0062381 | hsa_circ_0037277 |
| hsa_circ_0062383 | hsa_circ_0037280 |
| hsa_circ_0062399 | hsa_circ_0037281 |
| hsa_circ_00624 | hsa_circ_00372 |

| | |
|---|---|
| 10 | 83 |
| hsa_circ_0062442 | hsa_circ_0037294 |
| hsa_circ_0062443 | hsa_circ_0037296 |
| hsa_circ_0062444 | hsa_circ_0037297 |
| hsa_circ_0062445 | hsa_circ_0037319 |
| hsa_circ_0062446 | hsa_circ_0037320 |
| hsa_circ_0062469 | hsa_circ_0037325 |
| hsa_circ_0062465 | hsa_circ_0037408 |
| hsa_circ_0062466 | hsa_circ_0037434 |
| hsa_circ_0062467 | hsa_circ_0037436 |
| hsa_circ_0062520 | hsa_circ_0037437 |
| hsa_circ_0062524 | hsa_circ_0037438 |
| hsa_circ_0062527 | hsa_circ_0037448 |
| hsa_circ_0062528 | hsa_circ_0037439 |
| hsa_c | hsa_c |

| | |
|---|---|
| irc_0062529 | irc_0037440 |
| hsa_circ_0062530 | hsa_circ_0037444 |
| hsa_circ_0062531 | hsa_circ_0037445 |
| hsa_circ_0062532 | hsa_circ_0037446 |
| hsa_circ_0062533 | hsa_circ_0037518 |
| hsa_circ_0062534 | hsa_circ_0037519 |
| hsa_circ_0062535 | hsa_circ_0037520 |
| hsa_circ_0062536 | hsa_circ_0037521 |
| hsa_circ_0062537 | hsa_circ_0037522 |
| hsa_circ_0062538 | hsa_circ_0037523 |
| hsa_circ_0062569 | hsa_circ_0037735 |
| hsa_circ_0062568 | hsa_circ_0037738 |
| hsa_circ_0062573 | hsa_circ_0037739 |
| hsa_circ_0062574 | hsa_circ_0037740 |

| | |
|---|---|
| hsa_circ_0062604 | hsa_circ_0037742 |
| hsa_circ_0062751 | hsa_circ_0037743 |
| hsa_circ_0062752 | hsa_circ_0037744 |
| hsa_circ_0062785 | hsa_circ_0037815 |
| hsa_circ_0062787 | hsa_circ_0037856 |
| hsa_circ_0062791 | hsa_circ_0037860 |
| hsa_circ_0062793 | hsa_circ_0037863 |
| hsa_circ_0062796 | hsa_circ_0037864 |
| hsa_circ_0062797 | hsa_circ_0037865 |
| hsa_circ_0062798 | hsa_circ_0037866 |
| hsa_circ_0062799 | hsa_circ_0037867 |
| hsa_circ_0062800 | hsa_circ_0037868 |
| hsa_circ_0062801 | hsa_circ_0037914 |
| hsa_circ_00628 | hsa_circ_00379 |

| | |
|---|---|
| 02 | 29 |
| hsa_circ_0062805 | hsa_circ_0037930 |
| hsa_circ_0062807 | hsa_circ_0037931 |
| hsa_circ_0062810 | hsa_circ_0003420 |
| hsa_circ_0062817 | hsa_circ_0003427 |
| hsa_circ_0062818 | hsa_circ_0005409 |
| hsa_circ_0062820 | hsa_circ_0007965 |
| hsa_circ_0062821 | hsa_circ_001569 |
| hsa_circ_0062822 | hsa_circ_0038153 |
| hsa_circ_0062823 | hsa_circ_0038155 |
| hsa_circ_0062824 | hsa_circ_0038156 |
| hsa_circ_0062929 | hsa_circ_0038157 |
| hsa_circ_0062930 | hsa_circ_0038158 |
| hsa_circ_0062936 | hsa_circ_0038161 |
| hsa_c | hsa_c |

| | |
|---|---|
| irc_0062937 | irc_0038317 |
| hsa_circ_0062974 | hsa_circ_0038322 |
| hsa_circ_0062975 | hsa_circ_0038404 |
| hsa_circ_0062976 | hsa_circ_0005343 |
| hsa_circ_0008832 | hsa_circ_0009060 |
| hsa_circ_0062978 | hsa_circ_0038445 |
| hsa_circ_0062983 | hsa_circ_0038447 |
| hsa_circ_0062984 | hsa_circ_0038448 |
| hsa_circ_0062985 | hsa_circ_0038486 |
| hsa_circ_0062986 | hsa_circ_0038500 |
| hsa_circ_001284 | hsa_circ_0038632 |
| hsa_circ_0062990 | hsa_circ_0038633 |
| hsa_circ_0062991 | hsa_circ_0038634 |
| hsa_circ_0062992 | hsa_circ_0007807 |

| | |
|---|---|
| hsa_circ_0062993 | hsa_circ_0038771 |
| hsa_circ_0062994 | hsa_circ_0038779 |
| hsa_circ_0063056 | hsa_circ_0038780 |
| hsa_circ_0063059 | hsa_circ_0038782 |
| hsa_circ_0063060 | hsa_circ_0038786 |
| hsa_circ_0063290 | hsa_circ_0038790 |
| hsa_circ_0063291 | hsa_circ_0038797 |
| hsa_circ_0063292 | hsa_circ_0038802 |
| hsa_circ_0063295 | hsa_circ_0038805 |
| hsa_circ_0063296 | hsa_circ_0005602 |
| hsa_circ_0063297 | hsa_circ_0038832 |
| hsa_circ_0063298 | hsa_circ_0038833 |
| hsa_circ_0063299 | hsa_circ_0038834 |
| hsa_circ_00633 | hsa_circ_00388 |

| | |
|---|---|
| 00 | 35 |
| hsa_circ_0063301 | hsa_circ_0038836 |
| hsa_circ_0063302 | hsa_circ_0038837 |
| hsa_circ_0063303 | hsa_circ_0038838 |
| hsa_circ_0063304 | hsa_circ_0038839 |
| hsa_circ_0063308 | hsa_circ_0038845 |
| hsa_circ_0063309 | hsa_circ_0038886 |
| hsa_circ_0063310 | hsa_circ_0003096 |
| hsa_circ_0063311 | hsa_circ_0004082 |
| hsa_circ_0063312 | hsa_circ_0038892 |
| hsa_circ_0063313 | hsa_circ_0038893 |
| hsa_circ_0063314 | hsa_circ_0038894 |
| hsa_circ_0063315 | hsa_circ_0038895 |
| hsa_circ_0063316 | hsa_circ_0038896 |
| hsa_c | hsa_c |

| | |
|---|---|
| irc_0063364 | irc_0038910 |
| hsa_circ_0063393 | hsa_circ_0007742 |
| hsa_circ_0063392 | hsa_circ_0038994 |
| hsa_circ_0063394 | hsa_circ_0038997 |
| hsa_circ_0063416 | hsa_circ_0038998 |
| hsa_circ_0063632 | hsa_circ_0038999 |
| hsa_circ_0063648 | hsa_circ_0039000 |
| hsa_circ_0063649 | hsa_circ_0039004 |
| hsa_circ_0063650 | hsa_circ_0039005 |
| hsa_circ_0063651 | hsa_circ_0039011 |
| hsa_circ_0002246 | hsa_circ_0039012 |
| hsa_circ_0089819 | hsa_circ_0039013 |
| hsa_circ_0089894 | hsa_circ_0039035 |
| hsa_circ_0089893 | hsa_circ_0039039 |

| | |
|---|---|
| hsa_circ_0089908 | hsa_circ_0039056 |
| hsa_circ_0090037 | hsa_circ_0039075 |
| hsa_circ_0090104 | hsa_circ_0039087 |
| hsa_circ_0090105 | hsa_circ_0039088 |
| hsa_circ_0090107 | hsa_circ_0039090 |
| hsa_circ_0090108 | hsa_circ_0039095 |
| hsa_circ_0090109 | hsa_circ_0039097 |
| hsa_circ_0090110 | hsa_circ_0039098 |
| hsa_circ_0090111 | hsa_circ_0039099 |
| hsa_circ_0090163 | hsa_circ_0039105 |
| hsa_circ_0090171 | hsa_circ_0039106 |
| hsa_circ_0090201 | hsa_circ_0039123 |
| hsa_circ_0090202 | hsa_circ_0039128 |
| hsa_circ_00902 | hsa_circ_00391 |

| | |
|---|---|
| 03 | 49 |
| hsa_circ_0090288 | hsa_circ_0039175 |
| hsa_circ_0090289 | hsa_circ_0039176 |
| hsa_circ_0090294 | hsa_circ_0039228 |
| hsa_circ_0090362 | hsa_circ_0039229 |
| hsa_circ_0090386 | hsa_circ_0039230 |
| hsa_circ_0090393 | hsa_circ_0039314 |
| hsa_circ_0090395 | hsa_circ_0039315 |
| hsa_circ_0090396 | hsa_circ_0039316 |
| hsa_circ_0090397 | hsa_circ_0039330 |
| hsa_circ_0090399 | hsa_circ_0039331 |
| hsa_circ_0090405 | hsa_circ_0039332 |
| hsa_circ_0090406 | hsa_circ_0039336 |
| hsa_circ_0090407 | hsa_circ_0039564 |
| hsa_c | hsa_c |

| | |
|---|---|
| irc_0090408 | irc_0039566 |
| hsa_circ_0090410 | hsa_circ_0039569 |
| hsa_circ_0090411 | hsa_circ_0039742 |
| hsa_circ_0090412 | hsa_circ_0039743 |
| hsa_circ_0090413 | hsa_circ_0039744 |
| hsa_circ_0090414 | hsa_circ_0039745 |
| hsa_circ_0090415 | hsa_circ_0039747 |
| hsa_circ_0090496 | hsa_circ_0039748 |
| hsa_circ_0090531 | hsa_circ_0039750 |
| hsa_circ_0090536 | hsa_circ_0039762 |
| hsa_circ_0090537 | hsa_circ_0039796 |
| hsa_circ_0090538 | hsa_circ_0039797 |
| hsa_circ_0090539 | hsa_circ_0039805 |
| hsa_circ_0090540 | hsa_circ_0039806 |

| | |
|---|---|
| hsa_circ_0090541 | hsa_circ_0039807 |
| hsa_circ_0090542 | hsa_circ_0039808 |
| hsa_circ_0090546 | hsa_circ_0039809 |
| hsa_circ_0090547 | hsa_circ_0039810 |
| hsa_circ_0090548 | hsa_circ_0039811 |
| hsa_circ_0090551 | hsa_circ_0039812 |
| hsa_circ_0090552 | hsa_circ_0039813 |
| hsa_circ_0090553 | hsa_circ_0039832 |
| hsa_circ_0090575 | hsa_circ_0039835 |
| hsa_circ_0090639 | hsa_circ_0039926 |
| hsa_circ_0090835 | hsa_circ_0039937 |
| hsa_circ_0090836 | hsa_circ_0039939 |
| hsa_circ_0090854 | hsa_circ_0006437 |
| hsa_circ_00908 | hsa_circ_00399 |

| | |
|---|---|
| 55 | 48 |
| hsa_circ_0091027 | hsa_circ_0039949 |
| hsa_circ_0091029 | hsa_circ_0039950 |
| hsa_circ_0091030 | hsa_circ_0039951 |
| hsa_circ_0091179 | hsa_circ_0039959 |
| hsa_circ_0091180 | hsa_circ_0040059 |
| hsa_circ_0091210 | hsa_circ_0040070 |
| hsa_circ_0091372 | hsa_circ_0040071 |
| hsa_circ_0091378 | hsa_circ_0040072 |
| hsa_circ_0091380 | hsa_circ_0040073 |
| hsa_circ_0091384 | hsa_circ_0040074 |
| hsa_circ_0091385 | hsa_circ_0040125 |
| hsa_circ_0091387 | hsa_circ_0040126 |
| hsa_circ_0005213 | hsa_circ_0040127 |
| hsa_c | hsa_c |

| | |
|---|---|
| irc_0006727 | irc_0040128 |
| hsa_circ_0091408 | hsa_circ_0040130 |
| hsa_circ_0091437 | hsa_circ_0040131 |
| hsa_circ_0091438 | hsa_circ_0040132 |
| hsa_circ_0091584 | hsa_circ_0040133 |
| hsa_circ_0091583 | hsa_circ_0040134 |
| hsa_circ_0091729 | hsa_circ_0040135 |
| hsa_circ_0091730 | hsa_circ_0040208 |
| hsa_circ_0091726 | hsa_circ_0040338 |
| hsa_circ_0091732 | hsa_circ_0040348 |
| hsa_circ_0091733 | hsa_circ_0040349 |
| hsa_circ_0091755 | hsa_circ_0040351 |
| hsa_circ_0091756 | hsa_circ_0040352 |
| hsa_circ_0091806 | hsa_circ_0040354 |

| | |
|---|---|
| hsa_circ_0091807 | hsa_circ_0040355 |
| hsa_circ_0091808 | hsa_circ_0040357 |
| hsa_circ_0091832 | hsa_circ_0040358 |
| hsa_circ_0091833 | hsa_circ_0040359 |
| hsa_circ_0091834 | hsa_circ_001388 |
| hsa_circ_0091835 | hsa_circ_0040396 |
| hsa_circ_0091836 | hsa_circ_0040397 |
| hsa_circ_0091837 | hsa_circ_0040398 |
| hsa_circ_0091838 | hsa_circ_0040399 |
| hsa_circ_0091839 | hsa_circ_0040400 |
| hsa_circ_0091840 | hsa_circ_0040401 |
| hsa_circ_0091841 | hsa_circ_0040402 |
| hsa_circ_0091842 | hsa_circ_0040403 |
| hsa_circ_00918 | hsa_circ_00404 |

| | |
|---|---|
| 43 | 04 |
| hsa_circ_091844 | hsa_circ_040423 |
| hsa_circ_091845 | hsa_circ_040424 |
| hsa_circ_091846 | hsa_circ_040425 |
| hsa_circ_091847 | hsa_circ_040474 |
| hsa_circ_091848 | hsa_circ_040475 |
| hsa_circ_091849 | hsa_circ_040476 |
| hsa_circ_091850 | hsa_circ_040477 |
| hsa_circ_091851 | hsa_circ_040478 |
| hsa_circ_091852 | hsa_circ_040479 |
| hsa_circ_091853 | hsa_circ_005258 |
| hsa_circ_091854 | hsa_circ_006691 |
| hsa_circ_091855 | hsa_circ_040504 |
| hsa_circ_091856 | hsa_circ_040511 |
| hsa_c | hsa_c |

| | |
|---|---|
| irc_0091857 | irc_0040512 |
| hsa_circ_0091858 | hsa_circ_0040514 |
| hsa_circ_0091860 | hsa_circ_0040515 |
| hsa_circ_0091861 | hsa_circ_0040517 |
| hsa_circ_0091862 | hsa_circ_0040518 |
| hsa_circ_0091863 | hsa_circ_0040519 |
| hsa_circ_0091864 | hsa_circ_0040594 |
| hsa_circ_0091865 | hsa_circ_0040680 |
| hsa_circ_0091866 | hsa_circ_0040681 |
| hsa_circ_0091867 | hsa_circ_0040700 |
| hsa_circ_0091868 | hsa_circ_0040706 |
| hsa_circ_0091869 | hsa_circ_0005687 |
| hsa_circ_0091870 | hsa_circ_0040709 |
| hsa_circ_0091871 | hsa_circ_0040742 |

| | |
|---|---|
| hsa_circ_0091872 | hsa_circ_0003098 |
| hsa_circ_0091873 | hsa_circ_0040811 |
| hsa_circ_0091874 | hsa_circ_0040812 |
| hsa_circ_0091875 | hsa_circ_0040817 |
| hsa_circ_0091876 | hsa_circ_0040819 |
| hsa_circ_0092101 | hsa_circ_001574 |
| hsa_circ_0092102 | hsa_circ_0040958 |
| hsa_circ_0092103 | hsa_circ_0041137 |
| hsa_circ_0092104 | hsa_circ_0041143 |
| hsa_circ_0092105 | hsa_circ_0041192 |
| hsa_circ_0092106 | hsa_circ_0041373 |
| hsa_circ_0092107 | hsa_circ_0004192 |
| hsa_circ_0092108 | hsa_circ_0041426 |
| hsa_circ_00921 | hsa_circ_00414 |

| | | |
|---|---|---|
| | 09 | 27<br>hsa_circ_004142 9<br>hsa_circ_004143 0<br>hsa_circ_004145 7<br>hsa_circ_004145 8<br>hsa_circ_004147 8<br>hsa_circ_002899<br>hsa_circ_004148 3<br>hsa_circ_004148 4<br>hsa_circ_004152 4<br>hsa_circ_004160 5<br>hsa_circ_004160 7<br>hsa_circ_004160 9<br>hsa_circ_004161 0<br>hsa_c |

| | | |
|---|---|---|
| | irc_0041734 | |
| | hsa_circ_0041841 | |
| | hsa_circ_0041842 | |
| | hsa_circ_0041848 | |
| | hsa_circ_0041849 | |
| | hsa_circ_0041850 | |
| | hsa_circ_0041851 | |
| | hsa_circ_0041864 | |
| | hsa_circ_0041876 | |
| | hsa_circ_0041881 | |
| | hsa_circ_0041887 | |
| | hsa_circ_0041888 | |
| | hsa_circ_0041891 | |
| | hsa_circ_0041890 | |

| | | |
|---|---|---|
| | hsa_circ_004195 1 | |
| | hsa_circ_004195 2 | |
| | hsa_circ_004195 4 | |
| | hsa_circ_004197 7 | |
| | hsa_circ_004198 2 | |
| | hsa_circ_004198 3 | |
| | hsa_circ_004205 9 | |
| | hsa_circ_004206 1 | |
| | hsa_circ_004207 5 | |
| | hsa_circ_004207 6 | |
| | hsa_circ_004207 8 | |
| | hsa_circ_004208 7 | |
| | hsa_circ_004208 8 | |
| | hsa_circ_0045 | |

| | | |
|---|---|---|
| | 32<br>hsa_circ_0005603<br>hsa_circ_0007456<br>hsa_circ_0042092<br>hsa_circ_0042093<br>hsa_circ_0042094<br>hsa_circ_0042096<br>hsa_circ_0042097<br>hsa_circ_0042099<br>hsa_circ_0042098<br>hsa_circ_0042118<br>hsa_circ_0042130<br>hsa_circ_0042131<br>hsa_circ_0042176<br>hsa_c | |

| | | |
|---|---|---|
| | irc_0<br>042177<br>hsa_c<br>irc_0<br>042178<br>hsa_c<br>irc_0<br>042194<br>hsa_c<br>irc_0<br>042195<br>hsa_c<br>irc_0<br>042196<br>hsa_c<br>irc_0<br>042197<br>hsa_c<br>irc_0<br>042244<br>hsa_c<br>irc_0<br>042245<br>hsa_c<br>irc_0<br>042366<br>hsa_c<br>irc_0<br>042522<br>hsa_c<br>irc_0<br>042524<br>hsa_c<br>irc_0<br>042543<br>hsa_c<br>irc_0<br>042544<br>hsa_c<br>irc_0<br>042551 | |

| | | |
|---|---|---|
| | hsa_circ_0042552<br>hsa_circ_0042553<br>hsa_circ_0042554<br>hsa_circ_0042555<br>hsa_circ_0042703<br>hsa_circ_0042748<br>hsa_circ_0042771<br>hsa_circ_0042774<br>hsa_circ_0042778<br>hsa_circ_0042780<br>hsa_circ_0042781<br>hsa_circ_0042782<br>hsa_circ_0042791<br>hsa_circ_00427 | |

| | | |
|---|---|---|
| | 92<br>hsa_circ_004279 3<br>hsa_circ_004279 4<br>hsa_circ_004279 5<br>hsa_circ_000342 2<br>hsa_circ_000719 4<br>hsa_circ_000465 9<br>hsa_circ_004287 8<br>hsa_circ_004289 3<br>hsa_circ_004289 6<br>hsa_circ_004289 8<br>hsa_circ_004290 0<br>hsa_circ_004290 7<br>hsa_circ_004290 8<br>hsa_c | |

| | | |
|---|---|---|
| | irc_0<br>042912<br>hsa_c<br>irc_0<br>042910<br>hsa_c<br>irc_0<br>042911<br>hsa_c<br>irc_0<br>042936<br>hsa_c<br>irc_0<br>043091<br>hsa_c<br>irc_0<br>043144<br>hsa_c<br>irc_0<br>043145<br>hsa_c<br>irc_0<br>001960<br>hsa_c<br>irc_0<br>043154<br>hsa_c<br>irc_0<br>043155<br>hsa_c<br>irc_0<br>043160<br>hsa_c<br>irc_0<br>043161<br>hsa_c<br>irc_0<br>043162<br>hsa_c<br>irc_0<br>043166 | |

| | | |
|---|---|---|
| | hsa_circ_0043171 | |
| | hsa_circ_0043172 | |
| | hsa_circ_0043173 | |
| | hsa_circ_0043270 | |
| | hsa_circ_0043387 | |
| | hsa_circ_0043390 | |
| | hsa_circ_0043449 | |
| | hsa_circ_0043452 | |
| | hsa_circ_0043453 | |
| | hsa_circ_0007990 | |
| | hsa_circ_0043458 | |
| | hsa_circ_0043662 | |
| | hsa_circ_0043698 | |
| | hsa_circ_00437 | |

| | | |
|---|---|---|
| | 06<br>hsa_circ_0043715<br>hsa_circ_0043716<br>hsa_circ_0043719<br>hsa_circ_0043726<br>hsa_circ_0043732<br>hsa_circ_0043733<br>hsa_circ_0043739<br>hsa_circ_0043735<br>hsa_circ_0043740<br>hsa_circ_0043766<br>hsa_circ_0043767<br>hsa_circ_0043771<br>hsa_circ_0043772<br>hsa_c | |

| | | |
|---|---|---|
| | irc_0<br>0438<br>00<br>hsa_c<br>irc_0<br>0438<br>01<br>hsa_c<br>irc_0<br>0438<br>02<br>hsa_c<br>irc_0<br>0438<br>03<br>hsa_c<br>irc_0<br>0067<br>10<br>hsa_c<br>irc_0<br>0438<br>57<br>hsa_c<br>irc_0<br>0438<br>58<br>hsa_c<br>irc_0<br>0438<br>59<br>hsa_c<br>irc_0<br>0438<br>60<br>hsa_c<br>irc_0<br>0438<br>61<br>hsa_c<br>irc_0<br>0185<br>1<br>hsa_c<br>irc_0<br>0091<br>23<br>hsa_c<br>irc_0<br>0438<br>92<br>hsa_c<br>irc_0<br>0438<br>97 | |

| | | |
|---|---|---|
| | hsa_circ_004398<br>hsa_circ_0016 31<br>hsa_circ_004390 2<br>hsa_circ_004390 3<br>hsa_circ_004390 6<br>hsa_circ_004390 7<br>hsa_circ_004390 8<br>hsa_circ_004390 9<br>hsa_circ_004397 7<br>hsa_circ_004397 8<br>hsa_circ_004400 3<br>hsa_circ_004400 4<br>hsa_circ_004400 5<br>hsa_circ_0 0440 | |

| | | |
|---|---|---|
| | 10<br>hsa_circ_0003699<br>hsa_circ_0007378<br>hsa_circ_0044029<br>hsa_circ_0044075<br>hsa_circ_0044094<br>hsa_circ_0044145<br>hsa_circ_0044146<br>hsa_circ_0044147<br>hsa_circ_0044149<br>hsa_circ_0044151<br>hsa_circ_0044153<br>hsa_circ_0044154<br>hsa_circ_0044155<br>hsa_c | |

| | | |
|---|---|---|
| | irc_0<br>044156<br>hsa_c<br>irc_0<br>044157<br>hsa_c<br>irc_0<br>003434<br>hsa_c<br>irc_0<br>008067<br>hsa_c<br>irc_0<br>044175<br>hsa_c<br>irc_0<br>044176<br>hsa_c<br>irc_0<br>044177<br>hsa_c<br>irc_0<br>002960<br>hsa_c<br>irc_0<br>005639<br>hsa_c<br>irc_0<br>044331<br>hsa_c<br>irc_0<br>044356<br>hsa_c<br>irc_0<br>044362<br>hsa_c<br>irc_0<br>044398<br>hsa_c<br>irc_0<br>044401 | |

| | | |
|---|---|---|
| | hsa_circ_000444 03 hsa_circ_000444 04 hsa_circ_000444 11 hsa_circ_000445 02 hsa_circ_000445 03 hsa_circ_000445 04 hsa_circ_000445 05 hsa_circ_000445 06 hsa_circ_000445 07 hsa_circ_000445 08 hsa_circ_000445 09 hsa_circ_000445 10 hsa_circ_000445 11 hsa_circ_00445 | |

| | |
|---|---|
| | 71<br>hsa_circ_0044573<br>hsa_circ_0044710<br>hsa_circ_0044712<br>hsa_circ_0044714<br>hsa_circ_0044723<br>hsa_circ_0044768<br>hsa_circ_0044769<br>hsa_circ_0044828<br>hsa_circ_0008625<br>hsa_circ_0044899<br>hsa_circ_0044900<br>hsa_circ_0044901<br>hsa_circ_0044902<br>hsa_c |

| | | |
|---|---|---|
| | irc_0<br>0449<br>08<br>hsa_c<br>irc_0<br>0450<br>27<br>hsa_c<br>irc_0<br>0450<br>28<br>hsa_c<br>irc_0<br>0450<br>29<br>hsa_c<br>irc_0<br>0450<br>63<br>hsa_c<br>irc_0<br>0450<br>66<br>hsa_c<br>irc_0<br>0450<br>67<br>hsa_c<br>irc_0<br>0450<br>68<br>hsa_c<br>irc_0<br>0450<br>69<br>hsa_c<br>irc_0<br>0450<br>70<br>hsa_c<br>irc_0<br>0450<br>71<br>hsa_c<br>irc_0<br>0450<br>72<br>hsa_c<br>irc_0<br>0450<br>73<br>hsa_c<br>irc_0<br>0450<br>74 | |

| | | |
|---|---|---|
| | hsa_circ_004515 5<br>hsa_circ_004515 9<br>hsa_circ_004516 2<br>hsa_circ_004516 3<br>hsa_circ_004516 4<br>hsa_circ_000315 6<br>hsa_circ_004533 5<br>hsa_circ_000708 1<br>hsa_circ_004539 2<br>hsa_circ_004543 3<br>hsa_circ_004543 4<br>hsa_circ_004543 5<br>hsa_circ_004543 6<br>hsa_circ_0 0454 | |

37
hsa_c
irc_0
0454
38
hsa_c
irc_0
0454
39
hsa_c
irc_0
0454
40
hsa_c
irc_0
0454
41
hsa_c
irc_0
0454
42
hsa_c
irc_0
0173
0
hsa_c
irc_0
0454
74
hsa_c
irc_0
0454
78
hsa_c
irc_0
0454
82
hsa_c
irc_0
0454
83
hsa_c
irc_0
0454
84
hsa_c
irc_0
0454
87
hsa_c
irc_0
0454
94
hsa_c

| | | |
|---|---|---|
| | irc_0<br>0454<br>96<br>hsa_c<br>irc_0<br>0454<br>97<br>hsa_c<br>irc_0<br>0454<br>98<br>hsa_c<br>irc_0<br>0454<br>99<br>hsa_c<br>irc_0<br>0455<br>00<br>hsa_c<br>irc_0<br>0455<br>01<br>hsa_c<br>irc_0<br>0455<br>02<br>hsa_c<br>irc_0<br>0455<br>03<br>hsa_c<br>irc_0<br>0455<br>04<br>hsa_c<br>irc_0<br>0455<br>37<br>hsa_c<br>irc_0<br>0455<br>38<br>hsa_c<br>irc_0<br>0063<br>1<br>hsa_c<br>irc_0<br>0455<br>58<br>hsa_c<br>irc_0<br>0455<br>61 | |

| | | |
|---|---|---|
| | hsa_circ_004559 1 hsa_circ_004559 2 hsa_circ_004560 0 hsa_circ_004562 6 hsa_circ_004562 7 hsa_circ_004571 1 hsa_circ_004571 0 hsa_circ_004573 2 hsa_circ_004573 3 hsa_circ_004573 4 hsa_circ_004573 5 hsa_circ_004573 6 hsa_circ_002115 6 hsa_circ_0021 | |

| | | |
|---|---|---|
| | 57<br>hsa_circ_0045745<br>hsa_circ_0045747<br>hsa_circ_0014900<br>hsa_circ_0045795<br>hsa_circ_0045796<br>hsa_circ_0045797<br>hsa_circ_0045798<br>hsa_circ_0045799<br>hsa_circ_0045800<br>hsa_circ_0045801<br>hsa_circ_0045802<br>hsa_circ_0045803<br>hsa_circ_0045804<br>hsa_c | |

| | | |
|---|---|---|
| | irc_0<br>0458<br>18<br>hsa_c<br>irc_0<br>0458<br>19<br>hsa_c<br>irc_0<br>0458<br>22<br>hsa_c<br>irc_0<br>0458<br>21<br>hsa_c<br>irc_0<br>0458<br>45<br>hsa_c<br>irc_0<br>0458<br>46<br>hsa_c<br>irc_0<br>0458<br>61<br>hsa_c<br>irc_0<br>0458<br>63<br>hsa_c<br>irc_0<br>0458<br>64<br>hsa_c<br>irc_0<br>0458<br>67<br>hsa_c<br>irc_0<br>0088<br>86<br>hsa_c<br>irc_0<br>0458<br>90<br>hsa_c<br>irc_0<br>0458<br>91<br>hsa_c<br>irc_0<br>0458<br>93 | |

| | | |
|---|---|---|
| | hsa_circ_0045894 | |
| | hsa_circ_0045898 | |
| | hsa_circ_0045899 | |
| | hsa_circ_0045901 | |
| | hsa_circ_0045902 | |
| | hsa_circ_0045903 | |
| | hsa_circ_0045918 | |
| | hsa_circ_0045919 | |
| | hsa_circ_0045951 | |
| | hsa_circ_0045952 | |
| | hsa_circ_00240 | |
| | hsa_circ_0045955 | |
| | hsa_circ_0045956 | |
| | hsa_circ_00459 | |

| | | |
|---|---|---|
| | 59<br>hsa_circ_0003070<br>hsa_circ_0046003<br>hsa_circ_0046004<br>hsa_circ_0046007<br>hsa_circ_0046008<br>hsa_circ_0046009<br>hsa_circ_0046010<br>hsa_circ_0046014<br>hsa_circ_0046015<br>hsa_circ_0046016<br>hsa_circ_0046141<br>hsa_circ_0046142<br>hsa_circ_0046143<br>hsa_c | |

irc_0
0461
48
hsa_c
irc_0
0461
49
hsa_c
irc_0
0461
50
hsa_c
irc_0
0461
51
hsa_c
irc_0
0461
52
hsa_c
irc_0
0462
40
hsa_c
irc_0
0462
74
hsa_c
irc_0
0462
75
hsa_c
irc_0
0463
79
hsa_c
irc_0
0065
16
hsa_c
irc_0
0463
89
hsa_c
irc_0
0463
90
hsa_c
irc_0
0463
92
hsa_c
irc_0
0463
93

| | |
|---|---|
| | hsa_circ_0046394 |
| | hsa_circ_0046395 |
| | hsa_circ_0046822 |
| | hsa_circ_0046825 |
| | hsa_circ_0046826 |
| | hsa_circ_0046827 |
| | hsa_circ_0046829 |
| | hsa_circ_0046830 |
| | hsa_circ_0008281 |
| | hsa_circ_0046912 |
| | hsa_circ_0046913 |
| | hsa_circ_0046914 |
| | hsa_circ_0047000 |
| | hsa_circ_00470 |

| | |
|---|---|
| | 01<br>hsa_circ_004700 2<br>hsa_circ_004700 3<br>hsa_circ_004700 4<br>hsa_circ_004700 5<br>hsa_circ_004700 6<br>hsa_circ_004700 7<br>hsa_circ_004701 1<br>hsa_circ_004701 2<br>hsa_circ_004704 1<br>hsa_circ_004704 5<br>hsa_circ_004704 6<br>hsa_circ_004704 8<br>hsa_circ_000774 9<br>hsa_c |

| | | |
|---|---|---|
| | irc_0<br>047128<br>hsa_c<br>irc_0<br>047130<br>hsa_c<br>irc_0<br>047135<br>hsa_c<br>irc_0<br>047136<br>hsa_c<br>irc_0<br>047137<br>hsa_c<br>irc_0<br>01633<br>hsa_c<br>irc_0<br>047152<br>hsa_c<br>irc_0<br>047155<br>hsa_c<br>irc_0<br>047156<br>hsa_c<br>irc_0<br>003495<br>hsa_c<br>irc_0<br>004017<br>hsa_c<br>irc_0<br>047353<br>hsa_c<br>irc_0<br>047354<br>hsa_c<br>irc_0<br>047358 | |

| | | |
|---|---|---|
| | hsa_circ_004736 0 | |
| | hsa_circ_004736 1 | |
| | hsa_circ_004736 3 | |
| | hsa_circ_004738 4 | |
| | hsa_circ_004738 5 | |
| | hsa_circ_004738 7 | |
| | hsa_circ_004738 8 | |
| | hsa_circ_004739 1 | |
| | hsa_circ_004745 9 | |
| | hsa_circ_004746 1 | |
| | hsa_circ_004748 1 | |
| | hsa_circ_004748 2 | |
| | hsa_circ_004748 3 | |
| | hsa_circ_0474 | |

| | | |
|---|---|---|
| | 84 | |
| | hsa_circ_004786 | |
| | hsa_circ_004787 | |
| | hsa_circ_004788 | |
| | hsa_circ_004789 | |
| | hsa_circ_004790 | |
| | hsa_circ_004791 | |
| | hsa_circ_004752 3 | |
| | hsa_circ_0047524 | |
| | hsa_circ_0047532 | |
| | hsa_circ_0047533 | |
| | hsa_circ_0047537 | |
| | hsa_circ_0047538 | |
| | hsa_circ_0047539 | |
| | hsa_c | |

| | | |
|---|---|---|
| | irc_0<br>047540<br>hsa_c<br>irc_0<br>047541<br>hsa_c<br>irc_0<br>047542<br>hsa_c<br>irc_0<br>047603<br>hsa_c<br>irc_0<br>047666<br>hsa_c<br>irc_0<br>047667<br>hsa_c<br>irc_0<br>047681<br>hsa_c<br>irc_0<br>047683<br>hsa_c<br>irc_0<br>047689<br>hsa_c<br>irc_0<br>047690<br>hsa_c<br>irc_0<br>047691<br>hsa_c<br>irc_0<br>047692<br>hsa_c<br>irc_0<br>047693<br>hsa_c<br>irc_0<br>01953 | |

| | | |
|---|---|---|
| | hsa_circ_004780<br>hsa_circ_004781<br>hsa_circ_004785<br>hsa_circ_004787<br>hsa_circ_004790<br>hsa_circ_004958<br>hsa_circ_004959<br>hsa_circ_004802<br>hsa_circ_004805<br>hsa_circ_004806<br>hsa_circ_004807<br>hsa_circ_004812<br>hsa_circ_004813<br>hsa_circ_00481 | |

| | | |
|---|---|---|
| | 84<br>hsa_circ_0048185<br>hsa_circ_0048186<br>hsa_circ_0048187<br>hsa_circ_0048188<br>hsa_circ_0048189<br>hsa_circ_0048191<br>hsa_circ_0048211<br>hsa_circ_0048283<br>hsa_circ_0048284<br>hsa_circ_0048285<br>hsa_circ_0048286<br>hsa_circ_0048316<br>hsa_circ_0048354<br>hsa_c | |

irc_0
0483
55
hsa_c
irc_0
0483
56
hsa_c
irc_0
0483
57
hsa_c
irc_0
0483
58
hsa_c
irc_0
0483
59
hsa_c
irc_0
0483
60
hsa_c
irc_0
0483
61
hsa_c
irc_0
0483
62
hsa_c
irc_0
0483
66
hsa_c
irc_0
0484
43
hsa_c
irc_0
0484
44
hsa_c
irc_0
0484
45
hsa_c
irc_0
0484
46
hsa_c
irc_0
0484
47

| | | |
|---|---|---|
| | hsa_circ_004846<br>1<br>hsa_circ_004846<br>3<br>hsa_circ_004847<br>5<br>hsa_circ_004847<br>8<br>hsa_circ_004863<br>4<br>hsa_circ_004871<br>7<br>hsa_circ_004871<br>6<br>hsa_circ_004885<br>0<br>hsa_circ_004885<br>1<br>hsa_circ_004885<br>4<br>hsa_circ_004885<br>5<br>hsa_circ_004885<br>6<br>hsa_circ_004885<br>7<br>hsa_circ_0488 | |

| | | |
|---|---|---|
| | 58<br>hsa_circ_004885 9<br>hsa_circ_004886 0<br>hsa_circ_004886 1<br>hsa_circ_004886 2<br>hsa_circ_004886 3<br>hsa_circ_004892 0<br>hsa_circ_004892 3<br>hsa_circ_004894 0<br>hsa_circ_004894 1<br>hsa_circ_004899 1<br>hsa_circ_004901 0<br>hsa_circ_004903 9<br>hsa_circ_004904 0<br>hsa_c | |

| | | |
|---|---|---|
| | irc_0<br>049041<br>hsa_c<br>irc_0<br>049042<br>hsa_c<br>irc_0<br>049043<br>hsa_c<br>irc_0<br>049044<br>hsa_c<br>irc_0<br>049045<br>hsa_c<br>irc_0<br>049046<br>hsa_c<br>irc_0<br>049079<br>hsa_c<br>irc_0<br>049080<br>hsa_c<br>irc_0<br>049081<br>hsa_c<br>irc_0<br>049100<br>hsa_c<br>irc_0<br>049158<br>hsa_c<br>irc_0<br>049159<br>hsa_c<br>irc_0<br>049178<br>hsa_c<br>irc_0<br>049234 | |

| | | |
|---|---|---|
| | hsa_circ_004923 5 hsa_circ_004923 6 hsa_circ_004923 7 hsa_circ_004923 8 hsa_circ_004923 9 hsa_circ_004924 0 hsa_circ_004928 3 hsa_circ_004934 7 hsa_circ_004934 8 hsa_circ_004934 9 hsa_circ_004935 0 hsa_circ_004935 2 hsa_circ_004935 3 hsa_circ_0 0493 | |

| | | |
|---|---|---|
| | 54<br>hsa_circ_0049355<br>hsa_circ_0049356<br>hsa_circ_0049365<br>hsa_circ_0002579<br>hsa_circ_0049387<br>hsa_circ_0049389<br>hsa_circ_0049401<br>hsa_circ_0049402<br>hsa_circ_0049403<br>hsa_circ_0049404<br>hsa_circ_0049405<br>hsa_circ_0049499<br>hsa_circ_0049502<br>hsa_c | |

| | | |
|---|---|---|
| | irc_0<br>0495<br>03<br>hsa_c<br>irc_0<br>0495<br>57<br>hsa_c<br>irc_0<br>0495<br>59<br>hsa_c<br>irc_0<br>0495<br>60<br>hsa_c<br>irc_0<br>0495<br>61<br>hsa_c<br>irc_0<br>0495<br>62<br>hsa_c<br>irc_0<br>0495<br>63<br>hsa_c<br>irc_0<br>0495<br>64<br>hsa_c<br>irc_0<br>0495<br>65<br>hsa_c<br>irc_0<br>0495<br>66<br>hsa_c<br>irc_0<br>0495<br>67<br>hsa_c<br>irc_0<br>0495<br>94<br>hsa_c<br>irc_0<br>0495<br>95<br>hsa_c<br>irc_0<br>0495<br>96 | |

| | | |
|---|---|---|
| | hsa_circ_004959 7 | |
| | hsa_circ_004966 5 | |
| | hsa_circ_004971 6 | |
| | hsa_circ_004975 3 | |
| | hsa_circ_004975 4 | |
| | hsa_circ_004975 5 | |
| | hsa_circ_004975 6 | |
| | hsa_circ_004975 7 | |
| | hsa_circ_004975 8 | |
| | hsa_circ_004987 5 | |
| | hsa_circ_004987 7 | |
| | hsa_circ_004987 8 | |
| | hsa_circ_004987 9 | |
| | hsa_circ_00498 | |

| | | |
|---|---|---|
| | 81<br>hsa_c<br>irc_0<br>0498<br>82<br>hsa_c<br>irc_0<br>0498<br>83<br>hsa_c<br>irc_0<br>0062<br>49<br>hsa_c<br>irc_0<br>0071<br>71<br>hsa_c<br>irc_0<br>0499<br>13<br>hsa_c<br>irc_0<br>0499<br>14<br>hsa_c<br>irc_0<br>0499<br>15<br>hsa_c<br>irc_0<br>0499<br>16<br>hsa_c<br>irc_0<br>0499<br>17<br>hsa_c<br>irc_0<br>0499<br>18<br>hsa_c<br>irc_0<br>0499<br>19<br>hsa_c<br>irc_0<br>0499<br>20<br>hsa_c<br>irc_0<br>0499<br>80<br>hsa_c | |

irc_0
0501
12
hsa_c
irc_0
0501
13
hsa_c
irc_0
0501
14
hsa_c
irc_0
0501
15
hsa_c
irc_0
0501
69
hsa_c
irc_0
0501
72
hsa_c
irc_0
0501
73
hsa_c
irc_0
0501
96
hsa_c
irc_0
0501
97
hsa_c
irc_0
0501
98
hsa_c
irc_0
0501
99
hsa_c
irc_0
0502
33
hsa_c
irc_0
0502
48
hsa_c
irc_0
0505
67

| | | |
|---|---|---|
| | hsa_circ_005068<br>hsa_circ_005069<br>hsa_circ_005070<br>hsa_circ_005071<br>hsa_circ_005607<br>hsa_circ_005637<br>hsa_circ_005639<br>hsa_circ_005640<br>hsa_circ_005641<br>hsa_circ_005678<br>hsa_circ_005679<br>hsa_circ_005680<br>hsa_circ_005682<br>hsa_circ_00506 | |

| | | |
|---|---|---|
| | 85<br>hsa_circ_0050686<br>hsa_circ_0050687<br>hsa_circ_0050688<br>hsa_circ_0050689<br>hsa_circ_0050932<br>hsa_circ_0050953<br>hsa_circ_0051033<br>hsa_circ_0051034<br>hsa_circ_0051035<br>hsa_circ_0051036<br>hsa_circ_0051037<br>hsa_circ_0051038<br>hsa_circ_0051041<br>hsa_c | |

| | | |
|---|---|---|
| | irc_0<br>0510<br>43<br>hsa_c<br>irc_0<br>0510<br>44<br>hsa_c<br>irc_0<br>0510<br>90<br>hsa_c<br>irc_0<br>0510<br>91<br>hsa_c<br>irc_0<br>0510<br>96<br>hsa_c<br>irc_0<br>0510<br>97<br>hsa_c<br>irc_0<br>0511<br>00<br>hsa_c<br>irc_0<br>0511<br>01<br>hsa_c<br>irc_0<br>0511<br>02<br>hsa_c<br>irc_0<br>0511<br>04<br>hsa_c<br>irc_0<br>0511<br>05<br>hsa_c<br>irc_0<br>0511<br>06<br>hsa_c<br>irc_0<br>0511<br>07<br>hsa_c<br>irc_0<br>0511<br>08 | |

| | | |
|---|---|---|
| | hsa_circ_00028 1 | |
| | hsa_circ_051131 | |
| | hsa_circ_051135 | |
| | hsa_circ_051136 | |
| | hsa_circ_051137 | |
| | hsa_circ_051138 | |
| | hsa_circ_051265 | |
| | hsa_circ_051266 | |
| | hsa_circ_051267 | |
| | hsa_circ_003971 | |
| | hsa_circ_051327 | |
| | hsa_circ_051342 | |
| | hsa_circ_051414 | |
| | hsa_circ_0514 | |

| | | |
|---|---|---|
| | 47<br>hsa_circ_00514<br>48<br>hsa_circ_00514<br>49<br>hsa_circ_00514<br>50<br>hsa_circ_00514<br>51<br>hsa_circ_00514<br>52<br>hsa_circ_00514<br>53<br>hsa_circ_00514<br>54<br>hsa_circ_00514<br>55<br>hsa_circ_00514<br>56<br>hsa_circ_00514<br>66<br>hsa_circ_00514<br>68<br>hsa_circ_00514<br>73<br>hsa_circ_00514<br>74<br>hsa_c | |

| | | |
|---|---|---|
| | irc_0<br>051665<br>hsa_c<br>irc_0<br>051666<br>hsa_c<br>irc_0<br>051671<br>hsa_c<br>irc_0<br>051672<br>hsa_c<br>irc_0<br>051673<br>hsa_c<br>irc_0<br>051674<br>hsa_c<br>irc_0<br>051675<br>hsa_c<br>irc_0<br>051714<br>hsa_c<br>irc_0<br>051715<br>hsa_c<br>irc_0<br>051716<br>hsa_c<br>irc_0<br>051717<br>hsa_c<br>irc_0<br>051718<br>hsa_c<br>irc_0<br>051719<br>hsa_c<br>irc_0<br>051720 | |

| | | |
|---|---|---|
| | hsa_circ_0051721<br>hsa_circ_0051722<br>hsa_circ_0051723<br>hsa_circ_0051743<br>hsa_circ_0051744<br>hsa_circ_0051745<br>hsa_circ_0051746<br>hsa_circ_0051747<br>hsa_circ_0051802<br>hsa_circ_0051803<br>hsa_circ_0051804<br>hsa_circ_0051955<br>hsa_circ_0051956<br>hsa_circ_0520 | |

| | | |
|---|---|---|
| | 03<br>hsa_circ_0052005<br>hsa_circ_0052006<br>hsa_circ_0052007<br>hsa_circ_0052028<br>hsa_circ_0052115<br>hsa_circ_0052129<br>hsa_circ_0052136<br>hsa_circ_0052161<br>hsa_circ_0052162<br>hsa_circ_0052167<br>hsa_circ_0052168<br>hsa_circ_0052169<br>hsa_circ_0052201<br>hsa_c | |

| | | |
|---|---|---|
| | irc_0<br>052202<br>hsa_c<br>irc_0<br>052203<br>hsa_c<br>irc_0<br>052204<br>hsa_c<br>irc_0<br>052205<br>hsa_c<br>irc_0<br>052206<br>hsa_c<br>irc_0<br>052230<br>hsa_c<br>irc_0<br>052231<br>hsa_c<br>irc_0<br>052232<br>hsa_c<br>irc_0<br>052233<br>hsa_c<br>irc_0<br>052246<br>hsa_c<br>irc_0<br>052247<br>hsa_c<br>irc_0<br>052248<br>hsa_c<br>irc_0<br>052250<br>hsa_c<br>irc_0<br>052251 | |

| | | |
|---|---|---|
| | hsa_circ_0052252 | |
| | hsa_circ_0052253 | |
| | hsa_circ_0052254 | |
| | hsa_circ_0052255 | |
| | hsa_circ_0052256 | |
| | hsa_circ_0052257 | |
| | hsa_circ_0052258 | |
| | hsa_circ_0016334 | |
| | hsa_circ_0052300 | |
| | hsa_circ_0052307 | |
| | hsa_circ_0052309 | |
| | hsa_circ_0052329 | |
| | hsa_circ_0052333 | |
| | hsa_circ_00045 | |

| | | |
|---|---|---|
| | 25<br>hsa_circ_0059151<br>hsa_circ_0059255<br>hsa_circ_0059257<br>hsa_circ_0059327<br>hsa_circ_0059330<br>hsa_circ_0059420<br>hsa_circ_0059421<br>hsa_circ_0059495<br>hsa_circ_0009173<br>hsa_circ_0002176<br>hsa_circ_0059530<br>hsa_circ_0059531<br>hsa_circ_0059532<br>hsa_c | |

| | | |
|---|---|---|
| | irc_0<br>0595<br>49<br>hsa_c<br>irc_0<br>0595<br>63<br>hsa_c<br>irc_0<br>0595<br>80<br>hsa_c<br>irc_0<br>0595<br>81<br>hsa_c<br>irc_0<br>0595<br>82<br>hsa_c<br>irc_0<br>0595<br>87<br>hsa_c<br>irc_0<br>0595<br>88<br>hsa_c<br>irc_0<br>0595<br>92<br>hsa_c<br>irc_0<br>0595<br>93<br>hsa_c<br>irc_0<br>0595<br>96<br>hsa_c<br>irc_0<br>0595<br>97<br>hsa_c<br>irc_0<br>0595<br>98<br>hsa_c<br>irc_0<br>0596<br>14<br>hsa_c<br>irc_0<br>0596<br>86 | |

| | | |
|---|---|---|
| | hsa_circ_0059682<br>hsa_circ_0059683<br>hsa_circ_0059684<br>hsa_circ_0059685<br>hsa_circ_0059692<br>hsa_circ_0059694<br>hsa_circ_0007029<br>hsa_circ_0059790<br>hsa_circ_0059791<br>hsa_circ_0059852<br>hsa_circ_0059856<br>hsa_circ_0059879<br>hsa_circ_0059901<br>hsa_circ_0599 | |

| | | |
|---|---|---|
| | 02<br>hsa_c<br>irc_0<br>0599<br>03<br>hsa_c<br>irc_0<br>0599<br>04<br>hsa_c<br>irc_0<br>0599<br>05<br>hsa_c<br>irc_0<br>0599<br>27<br>hsa_c<br>irc_0<br>0599<br>31<br>hsa_c<br>irc_0<br>0599<br>32<br>hsa_c<br>irc_0<br>0599<br>39<br>hsa_c<br>irc_0<br>0599<br>41<br>hsa_c<br>irc_0<br>0599<br>42<br>hsa_c<br>irc_0<br>0599<br>44<br>hsa_c<br>irc_0<br>0599<br>45<br>hsa_c<br>irc_0<br>0599<br>46<br>hsa_c<br>irc_0<br>0599<br>47<br>hsa_c | |

| | | |
|---|---|---|
| | irc_001763 | |
| | hsa_circ_0059970 | |
| | hsa_circ_0059971 | |
| | hsa_circ_0059972 | |
| | hsa_circ_0059973 | |
| | hsa_circ_0059974 | |
| | hsa_circ_0059975 | |
| | hsa_circ_0018882 | |
| | hsa_circ_0060001 | |
| | hsa_circ_0060002 | |
| | hsa_circ_0060125 | |
| | hsa_circ_0060126 | |
| | hsa_circ_0060181 | |
| | hsa_circ_0060250 | |

hsa_c
irc_0
0603
05
hsa_c
irc_0
0603
07
hsa_c
irc_0
0603
09
hsa_c
irc_0
0603
10
hsa_c
irc_0
0603
11
hsa_c
irc_0
0603
12
hsa_c
irc_0
0603
13
hsa_c
irc_0
0603
19
hsa_c
irc_0
0603
20
hsa_c
irc_0
0603
21
hsa_c
irc_0
0603
22
hsa_c
irc_0
0603
36
hsa_c
irc_0
0603
45
hsa_c
irc_0
0603

| | | |
|---|---|---|
| | 46<br>hsa_c<br>irc_0<br>0603<br>47<br>hsa_c<br>irc_0<br>0603<br>97<br>hsa_c<br>irc_0<br>0604<br>77<br>hsa_c<br>irc_0<br>0604<br>98<br>hsa_c<br>irc_0<br>0604<br>99<br>hsa_c<br>irc_0<br>0605<br>00<br>hsa_c<br>irc_0<br>0605<br>01<br>hsa_c<br>irc_0<br>0605<br>02<br>hsa_c<br>irc_0<br>0605<br>03<br>hsa_c<br>irc_0<br>0605<br>04<br>hsa_c<br>irc_0<br>0605<br>05<br>hsa_c<br>irc_0<br>0605<br>06<br>hsa_c<br>irc_0<br>0605<br>56<br>hsa_c | |

| | | |
|---|---|---|
| | irc_0<br>060576<br>hsa_c<br>irc_0<br>060577<br>hsa_c<br>irc_0<br>060588<br>hsa_c<br>irc_0<br>060589<br>hsa_c<br>irc_0<br>060590<br>hsa_c<br>irc_0<br>060591<br>hsa_c<br>irc_0<br>004212<br>hsa_c<br>irc_0<br>007026<br>hsa_c<br>irc_0<br>060598<br>hsa_c<br>irc_0<br>060599<br>hsa_c<br>irc_0<br>060603<br>hsa_c<br>irc_0<br>060604<br>hsa_c<br>irc_0<br>060607<br>hsa_c<br>irc_0<br>060608 | |

| | | |
|---|---|---|
| | hsa_circ_006069 | |
| | hsa_circ_006061 1 | |
| | hsa_circ_00904 | |
| | hsa_circ_060642 | |
| | hsa_circ_060643 | |
| | hsa_circ_060819 | |
| | hsa_circ_060820 | |
| | hsa_circ_060823 | |
| | hsa_circ_060825 | |
| | hsa_circ_060838 | |
| | hsa_circ_060909 | |
| | hsa_circ_060946 | |
| | hsa_circ_060947 | |
| | hsa_circ_0609 | |

| | |
|---|---|
| | 48<br>hsa_circ_00110 7<br>hsa_circ_0060980<br>hsa_circ_0003128<br>hsa_circ_0060988<br>hsa_circ_0060989<br>hsa_circ_0061080<br>hsa_circ_0061083<br>hsa_circ_0061087<br>hsa_circ_0061088<br>hsa_circ_0061090<br>hsa_circ_0061091<br>hsa_circ_0061092<br>hsa_circ_0007609<br>hsa_c |

| | | |
|---|---|---|
| | irc_0<br>0611<br>83<br>hsa_c<br>irc_0<br>0611<br>84<br>hsa_c<br>irc_0<br>0611<br>85<br>hsa_c<br>irc_0<br>0038<br>90<br>hsa_c<br>irc_0<br>0613<br>02<br>hsa_c<br>irc_0<br>0061<br>58<br>hsa_c<br>irc_0<br>0613<br>92<br>hsa_c<br>irc_0<br>0613<br>97<br>hsa_c<br>irc_0<br>0615<br>10<br>hsa_c<br>irc_0<br>0043<br>39<br>hsa_c<br>irc_0<br>0615<br>44<br>hsa_c<br>irc_0<br>0615<br>45<br>hsa_c<br>irc_0<br>0615<br>46<br>hsa_c<br>irc_0<br>0615<br>47 | |

| | | |
|---|---|---|
| | hsa_circ_006154 8 | |
| | hsa_circ_006154 9 | |
| | hsa_circ_006155 0 | |
| | hsa_circ_006159 5 | |
| | hsa_circ_006159 6 | |
| | hsa_circ_006159 8 | |
| | hsa_circ_006160 0 | |
| | hsa_circ_006160 1 | |
| | hsa_circ_006160 2 | |
| | hsa_circ_006160 3 | |
| | hsa_circ_008116 0 | |
| | hsa_circ_006171 3 | |
| | hsa_circ_006188 0 | |
| | hsa_circ_00618 | |

| | | |
|---|---|---|
| | 89<br>hsa_circ_00185 4<br>hsa_circ_0061924<br>hsa_circ_0061928<br>hsa_circ_0061941<br>hsa_circ_0061944<br>hsa_circ_0061945<br>hsa_circ_0061947<br>hsa_circ_0061948<br>hsa_circ_0062020<br>hsa_circ_0062022<br>hsa_circ_0062024<br>hsa_circ_0062025<br>hsa_circ_0062026<br>hsa_c | |

| | | |
|---|---|---|
| | irc_0<br>0621<br>83 | |
| | hsa_c<br>irc_0<br>0622<br>37 | |
| | hsa_c<br>irc_0<br>0622<br>38 | |
| | hsa_c<br>irc_0<br>0622<br>39 | |
| | hsa_c<br>irc_0<br>0622<br>40 | |
| | hsa_c<br>irc_0<br>0622<br>41 | |
| | hsa_c<br>irc_0<br>0622<br>59 | |
| | hsa_c<br>irc_0<br>0622<br>60 | |
| | hsa_c<br>irc_0<br>0622<br>82 | |
| | hsa_c<br>irc_0<br>0623<br>34 | |
| | hsa_c<br>irc_0<br>0623<br>35 | |
| | hsa_c<br>irc_0<br>0623<br>39 | |
| | hsa_c<br>irc_0<br>0623<br>41 | |
| | hsa_c<br>irc_0<br>0623<br>46 | |

| | | |
|---|---|---|
| | hsa_circ_0062348 | |
| | hsa_circ_0062349 | |
| | hsa_circ_0062350 | |
| | hsa_circ_0062351 | |
| | hsa_circ_0062354 | |
| | hsa_circ_0062410 | |
| | hsa_circ_0062478 | |
| | hsa_circ_0062479 | |
| | hsa_circ_0004208 | |
| | hsa_circ_0008241 | |
| | hsa_circ_0062559 | |
| | hsa_circ_0062579 | |
| | hsa_circ_0062583 | |
| | hsa_circ_00625 | |

| | | |
|---|---|---|
| | 85<br>hsa_circ_0062589<br>hsa_circ_0062590<br>hsa_circ_0062692<br>hsa_circ_0062693<br>hsa_circ_0062694<br>hsa_circ_0062695<br>hsa_circ_0062696<br>hsa_circ_0062697<br>hsa_circ_0062698<br>hsa_circ_0062770<br>hsa_circ_0062772<br>hsa_circ_0062773<br>hsa_circ_0062818<br>hsa_c | |

| | | |
|---|---|---|
| | irc_0<br>062822<br>hsa_c<br>irc_0<br>062846<br>hsa_c<br>irc_0<br>062907<br>hsa_c<br>irc_0<br>008323<br>hsa_c<br>irc_0<br>062924<br>hsa_c<br>irc_0<br>062925<br>hsa_c<br>irc_0<br>062926<br>hsa_c<br>irc_0<br>062940<br>hsa_c<br>irc_0<br>062941<br>hsa_c<br>irc_0<br>062942<br>hsa_c<br>irc_0<br>062943<br>hsa_c<br>irc_0<br>062946<br>hsa_c<br>irc_0<br>063065<br>hsa_c<br>irc_0<br>063162 | |

| | | |
|---|---|---|
| | hsa_circ_0063163 | |
| | hsa_circ_0063180 | |
| | hsa_circ_0063181 | |
| | hsa_circ_0063182 | |
| | hsa_circ_0063183 | |
| | hsa_circ_0063193 | |
| | hsa_circ_0063238 | |
| | hsa_circ_0063299 | |
| | hsa_circ_0063302 | |
| | hsa_circ_0063347 | |
| | hsa_circ_0063386 | |
| | hsa_circ_0063387 | |
| | hsa_circ_0063388 | |
| | hsa_circ_00633 | |

92
hsa_c
irc_0
0633
93
hsa_c
irc_0
0634
68
hsa_c
irc_0
0634
69
hsa_c
irc_0
0634
74
hsa_c
irc_0
0634
75
hsa_c
irc_0
0085
61
hsa_c
irc_0
0164
1
hsa_c
irc_0
0213
2
hsa_c
irc_0
0636
47
hsa_c
irc_0
0217
5
hsa_c
irc_0
0636
55
hsa_c
irc_0
0636
56
hsa_c
irc_0
0636
57
hsa_c

| | | |
|---|---|---|
| | irc_0063719 | |
| | hsa_circ_0063728 | |
| | hsa_circ_0063735 | |
| | hsa_circ_0063739 | |
| | hsa_circ_0063741 | |
| | hsa_circ_0063742 | |
| | hsa_circ_0063778 | |
| | hsa_circ_0063798 | |
| | hsa_circ_0063799 | |
| | hsa_circ_0063800 | |
| | hsa_circ_0063801 | |
| | hsa_circ_0063802 | |
| | hsa_circ_0063803 | |
| | hsa_circ_0063882 | |

| | | |
|---|---|---|
| | hsa_circ_006389<br>hsa_circ_006391<br>hsa_circ_006396<br>hsa_circ_006397<br>hsa_circ_006398<br>hsa_circ_006399<br>hsa_circ_006395<br>hsa_circ_006395<br>hsa_circ_006395<br>hsa_circ_006395<br>hsa_circ_006395<br>hsa_circ_006395<br>hsa_circ_006396<br>hsa_circ_0639 | |

| | | |
|---|---|---|
| | 61<br>hsa_circ_0089772<br>hsa_circ_0089844<br>hsa_circ_0008943<br>hsa_circ_0089867<br>hsa_circ_0089869<br>hsa_circ_0089870<br>hsa_circ_0089934<br>hsa_circ_0089935<br>hsa_circ_0089936<br>hsa_circ_0089937<br>hsa_circ_0089938<br>hsa_circ_0090039<br>hsa_circ_0090048<br>hsa_c | |

| | | |
|---|---|---|
| | irc_0<br>0900<br>50<br>hsa_c<br>irc_0<br>0900<br>53<br>hsa_c<br>irc_0<br>0900<br>54<br>hsa_c<br>irc_0<br>0901<br>43<br>hsa_c<br>irc_0<br>0901<br>44<br>hsa_c<br>irc_0<br>0901<br>46<br>hsa_c<br>irc_0<br>0901<br>47<br>hsa_c<br>irc_0<br>0901<br>48<br>hsa_c<br>irc_0<br>0901<br>50<br>hsa_c<br>irc_0<br>0901<br>52<br>hsa_c<br>irc_0<br>0901<br>54<br>hsa_c<br>irc_0<br>0901<br>56<br>hsa_c<br>irc_0<br>0901<br>57<br>hsa_c<br>irc_0<br>0901<br>77 | |

| | |
|---|---|
| | hsa_circ_0090184 hsa_circ_0090185 hsa_circ_0090201 hsa_circ_0090203 hsa_circ_0090204 hsa_circ_0090205 hsa_circ_0090206 hsa_circ_0090227 hsa_circ_0090235 hsa_circ_0090243 hsa_circ_0090247 hsa_circ_0090248 hsa_circ_0090251 hsa_circ_0902 |

| | | |
|---|---|---|
| | 53<br>hsa_circ_0090254<br>hsa_circ_0090255<br>hsa_circ_0090257<br>hsa_circ_0090291<br>hsa_circ_0090292<br>hsa_circ_0090294<br>hsa_circ_00773<br>hsa_circ_0090327<br>hsa_circ_0090374<br>hsa_circ_0090375<br>hsa_circ_0090376<br>hsa_circ_0090377<br>hsa_circ_0090378<br>hsa_c | |

| | | |
|---|---|---|
| | irc_0<br>090379<br>hsa_c<br>irc_0<br>090380<br>hsa_c<br>irc_0<br>090426<br>hsa_c<br>irc_0<br>090427<br>hsa_c<br>irc_0<br>090430<br>hsa_c<br>irc_0<br>090431<br>hsa_c<br>irc_0<br>090432<br>hsa_c<br>irc_0<br>090462<br>hsa_c<br>irc_0<br>090463<br>hsa_c<br>irc_0<br>090464<br>hsa_c<br>irc_0<br>090465<br>hsa_c<br>irc_0<br>090466<br>hsa_c<br>irc_0<br>090467<br>hsa_c<br>irc_0<br>090468 | |

| | | |
|---|---|---|
| | hsa_circ_0090469 hsa_circ_0090470 hsa_circ_0090471 hsa_circ_0090496 hsa_circ_0090522 hsa_circ_0090523 hsa_circ_0090524 hsa_circ_0090530 hsa_circ_0090612 hsa_circ_0090614 hsa_circ_0090615 hsa_circ_0090622 hsa_circ_0090623 hsa_circ_0906 | |

| | | |
|---|---|---|
| | 24<br>hsa_circ_0090625<br>hsa_circ_0090626<br>hsa_circ_0090655<br>hsa_circ_0090657<br>hsa_circ_0090662<br>hsa_circ_0090666<br>hsa_circ_0090671<br>hsa_circ_0090672<br>hsa_circ_0090673<br>hsa_circ_0090678<br>hsa_circ_0090679<br>hsa_circ_0090685<br>hsa_circ_0090686<br>hsa_c | |

| | | |
|---|---|---|
| | irc_0<br>090688<br>hsa_c<br>irc_0<br>090691<br>hsa_c<br>irc_0<br>090693<br>hsa_c<br>irc_0<br>090696<br>hsa_c<br>irc_0<br>090697<br>hsa_c<br>irc_0<br>090698<br>hsa_c<br>irc_0<br>090699<br>hsa_c<br>irc_0<br>090704<br>hsa_c<br>irc_0<br>090705<br>hsa_c<br>irc_0<br>090706<br>hsa_c<br>irc_0<br>090707<br>hsa_c<br>irc_0<br>090713<br>hsa_c<br>irc_0<br>090714<br>hsa_c<br>irc_0<br>090715 | |

| | | |
|---|---|---|
| | hsa_circ_0090716 | |
| | hsa_circ_0090717 | |
| | hsa_circ_0090719 | |
| | hsa_circ_0090722 | |
| | hsa_circ_0090723 | |
| | hsa_circ_0090724 | |
| | hsa_circ_0090725 | |
| | hsa_circ_0090730 | |
| | hsa_circ_0090735 | |
| | hsa_circ_0090736 | |
| | hsa_circ_0090737 | |
| | hsa_circ_0090741 | |
| | hsa_circ_0090742 | |
| | hsa_circ_00907 | |

| | |
|---|---|
| | 43<br>hsa_c<br>irc_0<br>0907<br>44<br>hsa_c<br>irc_0<br>0907<br>45<br>hsa_c<br>irc_0<br>0907<br>46<br>hsa_c<br>irc_0<br>0907<br>48<br>hsa_c<br>irc_0<br>0907<br>49<br>hsa_c<br>irc_0<br>0907<br>52<br>hsa_c<br>irc_0<br>0908<br>03<br>hsa_c<br>irc_0<br>0908<br>83<br>hsa_c<br>irc_0<br>0908<br>93<br>hsa_c<br>irc_0<br>0908<br>95<br>hsa_c<br>irc_0<br>0908<br>99<br>hsa_c<br>irc_0<br>0909<br>00<br>hsa_c<br>irc_0<br>0909<br>02<br>hsa_c |

| | | |
|---|---|---|
| | irc_0<br>0909<br>03<br>hsa_c<br>irc_0<br>0909<br>04<br>hsa_c<br>irc_0<br>0909<br>28<br>hsa_c<br>irc_0<br>0909<br>30<br>hsa_c<br>irc_0<br>0909<br>31<br>hsa_c<br>irc_0<br>0909<br>32<br>hsa_c<br>irc_0<br>0910<br>27<br>hsa_c<br>irc_0<br>0910<br>70<br>hsa_c<br>irc_0<br>0910<br>73<br>hsa_c<br>irc_0<br>0910<br>81<br>hsa_c<br>irc_0<br>0911<br>06<br>hsa_c<br>irc_0<br>0911<br>07<br>hsa_c<br>irc_0<br>0911<br>08<br>hsa_c<br>irc_0<br>0911<br>09 | |

| | | |
|---|---|---|
| | hsa_c<br>irc_0<br>0911<br>61<br>hsa_c<br>irc_0<br>0911<br>62<br>hsa_c<br>irc_0<br>0911<br>63<br>hsa_c<br>irc_0<br>0911<br>80<br>hsa_c<br>irc_0<br>0912<br>13<br>hsa_c<br>irc_0<br>0913<br>50<br>hsa_c<br>irc_0<br>0913<br>56<br>hsa_c<br>irc_0<br>0913<br>57<br>hsa_c<br>irc_0<br>0913<br>58<br>hsa_c<br>irc_0<br>0914<br>04<br>hsa_c<br>irc_0<br>0914<br>05<br>hsa_c<br>irc_0<br>0914<br>06<br>hsa_c<br>irc_0<br>0914<br>33<br>hsa_c<br>irc_0<br>0914 | |

| | | |
|---|---|---|
| | 96<br>hsa_circ_009149<br>8<br>hsa_circ_009149<br>9<br>hsa_circ_009150<br>0<br>hsa_circ_009150<br>1<br>hsa_circ_009150<br>3<br>hsa_circ_009150<br>5<br>hsa_circ_009151<br>1<br>hsa_circ_009151<br>2<br>hsa_circ_009167<br>7<br>hsa_circ_009173<br>6<br>hsa_circ_009173<br>7<br>hsa_circ_009173<br>8<br>hsa_circ_009175<br>1<br>hsa_c | |

| | | |
|---|---|---|
| | irc_0<br>091772<br>hsa_c<br>irc_0<br>091773<br>hsa_c<br>irc_0<br>091803<br>hsa_c<br>irc_0<br>091804<br>hsa_c<br>irc_0<br>091805<br>hsa_c<br>irc_0<br>091806<br>hsa_c<br>irc_0<br>091813<br>hsa_c<br>irc_0<br>091807<br>hsa_c<br>irc_0<br>091808<br>hsa_c<br>irc_0<br>091809<br>hsa_c<br>irc_0<br>091810<br>hsa_c<br>irc_0<br>091811<br>hsa_c<br>irc_0<br>091831<br>hsa_c<br>irc_0<br>091832 | |

hsa_c
irc_0
0918
33
hsa_c
irc_0
0918
34
hsa_c
irc_0
0918
35
hsa_c
irc_0
0918
36
hsa_c
irc_0
0918
37
hsa_c
irc_0
0918
38
hsa_c
irc_0
0918
39
hsa_c
irc_0
0918
40
hsa_c
irc_0
0918
41
hsa_c
irc_0
0918
42
hsa_c
irc_0
0918
43
hsa_c
irc_0
0918
44
hsa_c
irc_0
0918
45
hsa_c
irc_0
0918

| | | |
|---|---|---|
| | 46<br>hsa_circ_0091847<br>hsa_circ_0091848<br>hsa_circ_0091849<br>hsa_circ_0091850<br>hsa_circ_0091851<br>hsa_circ_0091852<br>hsa_circ_0091853<br>hsa_circ_0091854<br>hsa_circ_0091855<br>hsa_circ_0091856<br>hsa_circ_0091857<br>hsa_circ_0091858<br>hsa_circ_0091859<br>hsa_c | |

| | | |
|---|---|---|
| | irc_0091860 | |
| | hsa_circ_0091861 | |
| | hsa_circ_0091862 | |
| | hsa_circ_0091863 | |
| | hsa_circ_0091864 | |
| | hsa_circ_0091865 | |
| | hsa_circ_0091866 | |
| | hsa_circ_0091867 | |
| | hsa_circ_0005133 | |
| | hsa_circ_0092076 | |
| | hsa_circ_0092079 | |
| | hsa_circ_0092080 | |
| | hsa_circ_0092082 | |
| | hsa_circ_0092083 | |

| |
|---|
| hsa_circ_0092084 |
| hsa_circ_0092085 |
| hsa_circ_0092086 |
| hsa_circ_0092087 |
| hsa_circ_0092088 |
| hsa_circ_0092089 |
| hsa_circ_0092158 |
| hsa_circ_0092179 |
| hsa_circ_0007873 |
| hsa_circ_0092263 |
